# Whole-genome resequencing reveals genetic diversity and selection characteristics of dairy goat

**DOI:** 10.3389/fgene.2022.1044017

**Published:** 2023-01-06

**Authors:** Jinke Xiong, Jingjing Bao, Wenping Hu, Mingyu Shang, Li Zhang

**Affiliations:** Institute of Animal Science, Chinese Academy of Agricultural Sciences, Beijing, China

**Keywords:** dairy goat, genetic diversity, population structure, selective signal, milk production traits

## Abstract

The dairy goat is one of the earliest dairy livestock species, which plays an important role in the economic development, especially for developing countries. With the development of agricultural civilization, dairy goats have been widely distributed across the world. However, few studies have been conducted on the specific characteristics of dairy goat. In this study, we collected the whole-genome data of 89 goat individuals by sequencing 48 goats and employing 41 publicly available goats, including five dairy goat breeds (Saanen, Nubian, Alpine, Toggenburg, and Guanzhong dairy goat; *n* = 24, 15, 11, 6, 6), and three goat breeds (Guishan goat, Longlin goat, Yunshang Black goat; *n* = 6, 15, 6). Through compared the genomes of dairy goat and non-dairy goat to analyze genetic diversity and selection characteristics of dairy goat. The results show that the eight goats could be divided into three subgroups of European, African, and Chinese indigenous goat populations, and we also found that Australian Nubian, Toggenburg, and Australian Alpine had the highest linkage disequilibrium, the lowest level of nucleotide diversity, and a higher inbreeding coefficient, indicating that they were strongly artificially selected. In addition, we identified several candidate genes related to the specificity of dairy goat, particularly genes associated with milk production traits (*GHR*, *DGAT2*, *ELF5*, *GLYCAM1*, *ACSBG2*, *ACSS2*), reproduction traits (*TSHR*, *TSHB*, *PTGS2*, *ESR2*), immunity traits (*JAK1*, *POU2F2*, *LRRC66*). Our results provide not only insights into the evolutionary history and breed characteristics of dairy goat, but also valuable information for the implementation and improvement of dairy goat cross breeding program.

## 1 Introduction

The domestication of animals and plants facilitated the emergence of Neolithic agriculture. The goat is one of the oldest livestock species, which was domesticated about 10,000 years ago in the Zagros Mountains of the Fertile Crescent (Zeder and Hesse, 2000). Originally, goats were raised for meat. However, around 5,000 years ago, humans began to obtain secondary products from goats, including wool, skin, and milk (Chessa et al., 2009). With the development of agricultural civilization, humans began to select goat breeds according to the products they needed, such as milk goat, meat goat, and wool goat, and represents the earliest known instance of the breeding of goats (Alberto et al., 2018). The dairy goat is one of the most important dairy livestock species, not only providing humans with nutritious milk, but also meat, wool, and animal hide, as well as playing a vital role in animal husbandry. With the migrations of humans, there are 220 million individuals of over 200 dairy goat breeds are found across the world (FAOSTAT, 2020). After long-term artificial selection, the milk production characteristics of the modern dairy goat has become more significant. Saanen, Alpine, and Toggenburg are famous for high milk yield, with an average milk yield of 900 kg in 305 days, while Indian Beetal milk production in 190 days is 200 kg (Devendra and Haenlein, 2016).

Goats are considered the “cows of the poor.” As early as the Neolithic period, humans began to raise small groups of goats to obtain milk, producing milk much earlier than cow (Zervas and Tsiplakou, 2011). Compared with cow milk, goat milk has a higher content of growth active factors and is more similar to human milk, with the characteristics of small milk fat particles, easy digestion, and hypoallergenic properties (Clark and Mora García, 2017; Gallier et al., 2020). Furthermore, goat milk has a lower concentration of αs1-casein and a higher concentration of *β*-Casein, and can be used to produces cheese that is softer and higher in moisture than cow’s milk. Thus, goat milk is favored by consumers as a high-quality milk. However, the price of this milk is much higher than cow’s milk because of its high nutritional value and low yield. Furthermore, as the main food source of newborn goats, goat milk plays an important role in the growth and development of offspring. However, the prolific nanny goat is usually unable to feed multiple offspring, which leads to the decline of the survival rate and restricts the development of industry. Therefore, there is a need important to identify the genes related to milk production traits in order to cultivate goat breeds with high milk yields.

Natural and artificial selection have left footprints on the domestic animal genome, and genome-wide selection signal detection has become an important method to explore breed specificity and selection signatures in domestic animals (Qanbari and Simianer, 2014). Milk production traits are important economic traits and the most significant selection characteristics of dairy goat. At present, studies on candidate genes for milk production traits have been carried out in goat and sheep, such as Assaf sheep (Marina et al., 2020), Churra sheep (García-Gámez et al., 2012), Valle del Belice sheep (Di Gerlando et al., 2019), Alpine and Nubian (Scholtens et al., 2020), and some genes related to milk production have been revealed (*LALBA*, *SPP1*, *ABCG2*). However, previous studies have mainly used SNP arrays, which resulted in limited SNP sites and coverage depth, as well as potential deviations in the chip design process. In addition, these studies have used goat and sheep reference genomes rather than dairy goat or sheep reference genomes, which would result in incomplete variation detection. Thus, many selection signals may have remained undiscovered.

In this study, we used high-quality dairy goat reference genome (Saanen_v1) (Li et al., 2021b) and the whole genome re-sequencing data of 89 goats to conduct the comparative genomic analysis of dairy goats and non-dairy goats. We not only investigated the genetic relationships and population structure among dairy goat breeds, but also revealed the genetic diversity of dairy goat breeds. In addition, we applied two different statistics approach, the ratio of nucleotide diversity (θπ_non-dairy/dairy_) and the fixation index value (*F*_ST_), to detect selection signature for populations of dairy goats. We further identified genes related to milk production traits and provided a potential theoretical basis for dairy goat breeding.

## 2 Materials and methods

### 2.1 Sample collection, DNA extraction, and sequencing

**FIGURE 1:**
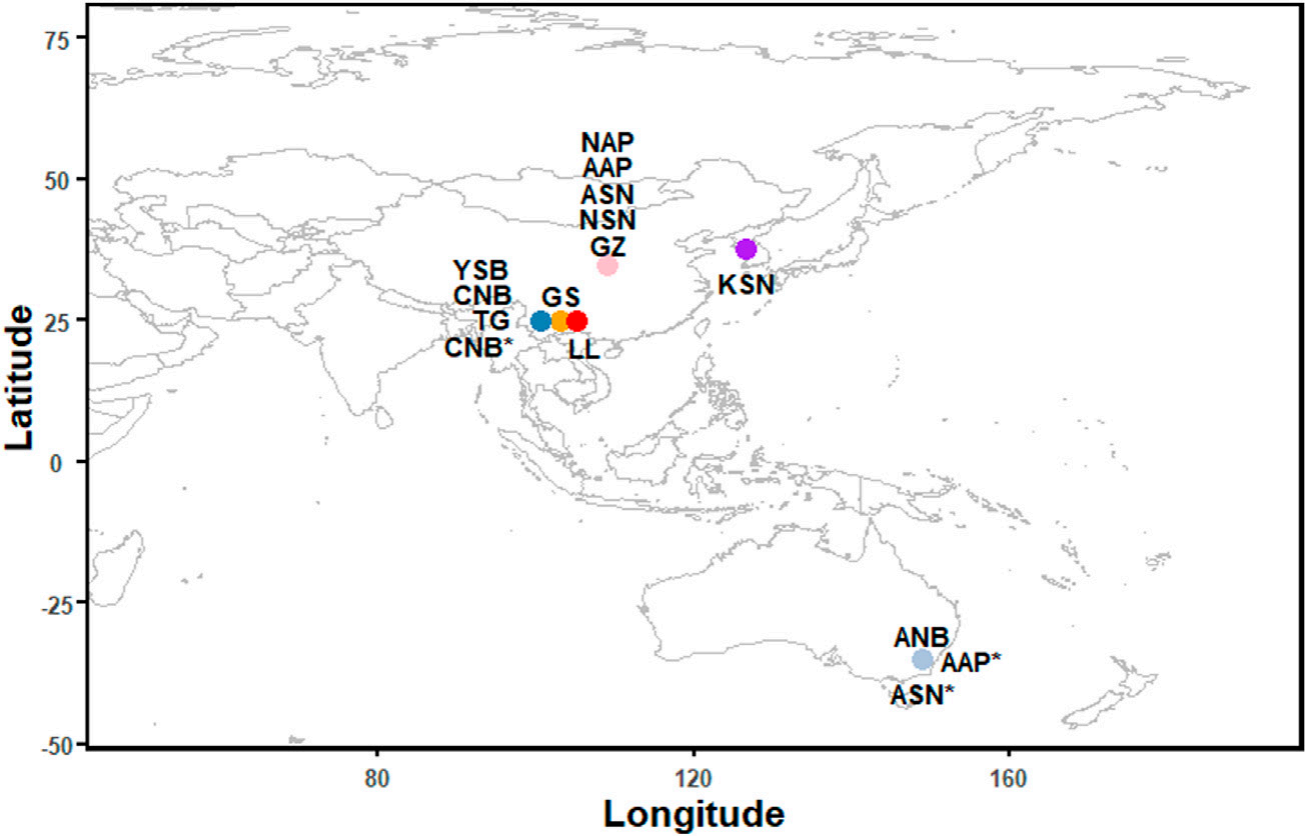
Sample information and geographic distribution of the 89 goats included in this study. [Guishan goat (GS, *n* = 6), Yunshang black goat (YSB, *n* = 6), Toggenburg (TG, *n* = 6), and Chinese Nubian (CNB, *n* = 6) were collected from Yunnan Province, Guanzhong dairy goat (GZ, *n* = 6), Australian Saanen (ASN, *n* = 6), New Zealand Saanen (NSN, *n* = 6), Australian Alpine (AAP, *n* = 2) and New Zealand Alpine (NAP, *n* = 4) were collected from Shanxi Province. Australian Alpine* (AAP*, *n* = 5), Longlin goat (LL, *n* = 15), Australian Nubian (ANB, *n* = 5), Chinese Nubian* (CNB*, *n* = 4), Korean Saanen (KSN, *n* = 10), Australian Saanen* (ASN*, *n* = 2) were downloaded from NCBI].

Eight goat populations were collected from Yunnan and Shanxi provinces as follows: Guishan goat (GS, *n* = 6), Yunshang Black goat (YSB, *n* = 6), Toggenburg (TG, *n* = 6), and Nubian (CNB, *n* = 6) were collected from Yunnan Province; Guanzhong dairy goat (GZ, *n* = 6), Australian Saanen (ASN, *n* = 6), New Zealand Saanen (NSN, *n* = 6), New Zealand Alpine (NAP, *n* = 4) and Australian Alpine (AAP, *n* = 2) were collected from Shanxi Province (Figure 1). We collected 5 ml blood from the jugular vein of each goat. The genomic DNA was extracted from the blood using the standard phenol-chloroform method. The qualified genomic DNA was used to construct a paired-end library with an insert size of 350 bp, which was then sequenced on the Illumina NovaSeq PE150 platform at the Novogene Bioinformatics Technology Company (Beijing, China). In addition, to make the selection analysis between dairy and non-dairy goat more reliable, we also downloaded 41 publicly-available genomes of six populations (Australian Alpine (AAP*, *n* = 5), Longlin goat (LL, *n* = 15), Australian Nubian (ANB, *n* = 5), Chinese Nubian (CNB*, *n* = 4), Korean Saanen (KSN, *n* = 10), and Australian Saanen (ASN*, *n* = 2) from NCBI to increase the sample size.

### 2.2 Sequence read mapping and variant annotation

The paired-end reads of the 89 samples were mapped onto the goat reference genome Saanen_v1 (https://www.ncbi.nlm.nih.gov/assembly/GCA_015443085.1) by the Burrows-Wheeler Aligner v.0.7.8 (Li and Durbin, 2009) using the parameters (Parameters: mem-t 4-k 32 -M). The mapped BAM files were sorted SAMtools v.1.3.1 (Li et al., 2009), and duplicate reads were removed using Picard toolkit (http://broadinstitute.github.io/picard). Then SAMtools (mpileup-m 2-F 0.002-d 10000000) and Genome Analysis Toolkit (GATK v.3.7) Unified Genotyper (McKenna et al., 2010) were used to extract the variant, and GATK Variant Filtration was used to detect the SNP of 89 samples to screen for high-quality SNPs. The filter settings were as follows: Quality by Depth (QD) <2.0, SNPs with missing rates (Miss) ≥.1, and minor allele frequencies (MAF) <0.05. All sample sets of filtered variant calls were used for imputation and phasing using Beagle v5.2 software (Browning et al., 2021). All SNPs were annotated using the ANNOVAR (Wang et al., 2010) based on the GFF file of the goat reference genome Saanen_v1.

### 2.3 Population genetic analysis

We used MEGA X to construct a neighbor-joining (NJ) tree for all samples based on the pairwise Hamming genetic distances matrix supplied by PLINK v1.9 (Purcell et al., 2007). The resulting NJ tree was visualized using Evolview (Subramanian et al., 2019). Principal component analysis (PCA) was performed using PLINK v1.9. In addition, the population structure was assessed using ADMIXTURE v1.3.0 (Alexander et al., 2009) with default setting. The number of assumed ancestry population K ranged from 2 to 8.

### 2.4 General genomic characteristics

Linkage disequilibrium (LD) coefficients between all pairwise SNPs in each population were calculated using PopLDdecay v3.40 (Zhang et al., 2019). Nucleotide diversity (π) was calculated for each population using a 25-kb sliding window with a 50-kb window size using VCFtools (Danecek et al., 2011). The runs of homozygosity (ROH) of each individual was detected by PLINKv1.9, and the genomic inbreeding coefficient of each individual based on ROH (*F*_ROH_) was calculated. Then, one-way ANOVA (analysis of variance) was used to test the significance between nucleotide diversity and inbreeding coefficient of each population. To understand the evolutionary history of the population, SNePv1.1 (Barbato et al., 2015) was used to estimate the effective population size (*Ne*) of each population before 1,000 generations.

### 2.5 Detection of selective sweeps

According to goat breed specificity, we divided the 89 goats into dairy goats and non-dairy goats. Then, we compared the genomes of dairy goats to those of the non-dairy goats. Selective signal sweep regions were detected according to the combinations of two parameters: the ratio of nucleotide diversity (θπ_non-dairy/dairy_) and fixation index value (*F*_ST_), which were estimated using VCFtools with a 50 kb sliding window and a 25 kb sliding step. In addition, we also calculated Tajima’s D statistic using VCFtools with a 25-kb window size to confirm the selective signals at chromosome 13. Candidate genes were annotated by the sweep regions detected from the intersection of the two parameters with a top 5% threshold, Bedtools (Quinlan and Hall, 2010) was used to annotate the selected regions for subsequent analysis.

### 2.6 Functional enrichment analysis

Enrichment analysis was performed using Gene Ontology (GO) and Kyoto Encyclopedia of Genes and Genomes (KEGG) pathway analysis to identify functional clusters of candidate genes. GO and KEGG enrichment analyses were performed using the OmicShare tool (http://www.omicshare.com/tools) and the number of significant genes for each term was determined using *p* ≤ 0.05 as the significance threshold.

## 3 Results

### 3.1 Genome sequence mapping and SNPs

The whole-genome data for 48 goats was generated and publicly available whole genome data for an additional 41 goats was collected. The whole-genome data of the 89 individual goats comprised that of eight goat breeds Guishan goat (GS), Yunshang Black goat (YSB), Longlin goat (LL), Toggenburg (TG), Guanzhong dairy goat (GZ), Nubian, Saanen, Alpine. The Nubian and Alpine breeds consist of three sub-populations [Australia Nubian (ANB), Chinese Nubian (CNB), Chinese Nubian (CNB*), and Australia Alpine (AAP), New Zealand Alpine (NAP), Australia Alpine (AAP*), respectively], while the Saanen breed consists of four sub-populations [New Zealand Saanen (NSN), Korean Saanen (KSN), Australia Saanen (ASN), Australia Saanen (ASN*)]. We generated a total of 4857G of clean data with an average depth of 31.15× for 48 samples using the whole genome sequencing method. The average sequence depth was 12.45× for 41 publicly-available genomes of six goat populations. We aligned the 89 goats 6382.6G of data with the Saanen reference genome (Saanen_v1). The average alignment rate was 99.7% and covered 98% of the reference genome. SNP is the most common genetic variation, after mapping and SNP calling, a total of 1,87,96,804 SNPs were identified from the 89 goat samples. The transitions/transversions ratio was 2.338, which is similar to the results obtained for others goats (Guan et al., 2016). The majority of the SNPs were found in the intergenic (66.05%) and intronic (29.86%) regions, while only a small number of the SNPs (0.407%) with 29,550 non-synonymous SNPs and 46,749 synonymous SNPs were found in the exonic region (Table 1).

**TABLE 1:** Summary and annotation of SNPs.

Category		Number of SNPs	Percentage of total (%)
Upstream		1,17,141	0.62
Exonic	Stop gain	236	0.0013
	Stop loss	40	0.00021
	Synonymous	46,749	0.25
	Non-synonymous	29,550	0.16
Intronic		56,12,654	29.86
Splicing		290	0.0015
Downstream		1,23,076	0.66
Upstream/Downstream		3,906	0.02
Intergenic		1,24,14,709	66.05
ts		1,31,65,437	
tv		56,30,367	
ts/tv		2.338	
Total		1,87,95,804	

### 3.2 Population structure and relationships

**FIGURE 2:**
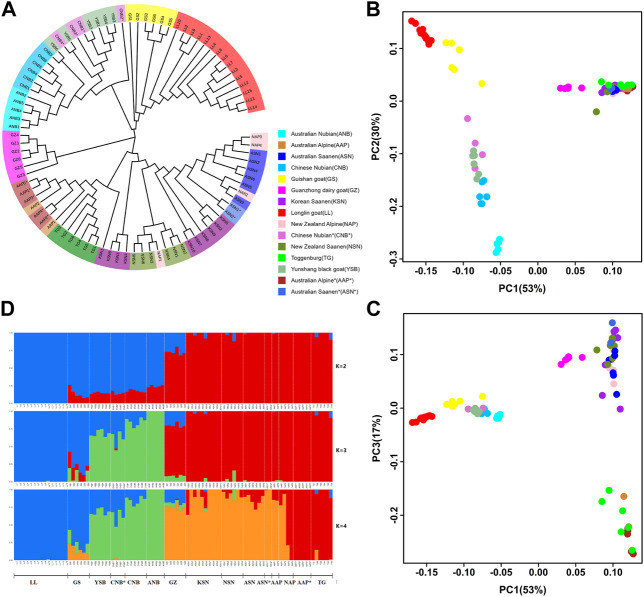
**(A)** Neighbor-joining (NJ) tree of the 89 individuals based on the matrix of Hamming genetic distance, different colors represent different populations. **(B)** Plots of the first and the second principal components for the 89 individuals. **(C)** Plots of the first and the third principal components for the 89 individuals. **(D)** Ancestry proportions of each sample using k = 2–4.

To identify the genetic and evolutionary relationship among the 15 goat populations, neighbor-joining (NJ) tree, principal component analysis (PCA) and population structure analysis were used. Based on autosomal SNPs, we constructed an NJ tree for the 89 individuals. The results showed that the 89 goats were divided into four groups, and the same goats breed from different regions were grouped (Figure 2A). First of all, Saanen (ASN, NSN, KSN, and ASN*), Alpine (AAP, NAP, and AAP*), TG were clustered together, while GZ was closely related to them. Then, Nubian (ANB, CNB, and CNB*) and YSB were clustered. Finally, GS and LL were clustered. To further explore the genetic relationship between goat breeds, we performed principal component analysis (PCA) (Figures 2B, C). The first eigenvector (PC1) explained 53% of the total genetic variation, from which the goat populations were divided into the Asian-African and European goat populations. The second eigenvector (PC2) explained 30% of the total genetic variation and separated the goats into Asian (LL, GS) and African (YSB, ANB, CNB, CNB*) goat populations. The third eigenvector (PC3) explained 17% of the total genetic variation, clearly distinguishing the Saanen (ASN, NSN, KSN, ASN*) and GZ from the European population. In the structure analysis calculated ranging from K = 2 to K = 8, we obtained the most reasonable biological interpretation and minimum crossover error at K = 4. At K = 2, LL, GS, YSB, CNB*, CNB, and ANB were separated with having a common genomic composition (blue color), indicating that the Asian-African and European populations were separated. At K = 4, Saanen (ASN, NSN, KSN, and ASN*) and GZ were further separated, and GZ was found to have a highly mixed genomic composition, which is consistent with the history of breed development (Figure 2D). Based on the results of the population genetic relationship analysis, the same breeds from the same regions were combined to increase the sample size, such as Australian Saanen [ASN (ASN and ASN*)] and Australian Alpine [AAP (AAP and AAP*)].

### 3.3 Population genetic diversity genomic characteristics

**FIGURE 3:**
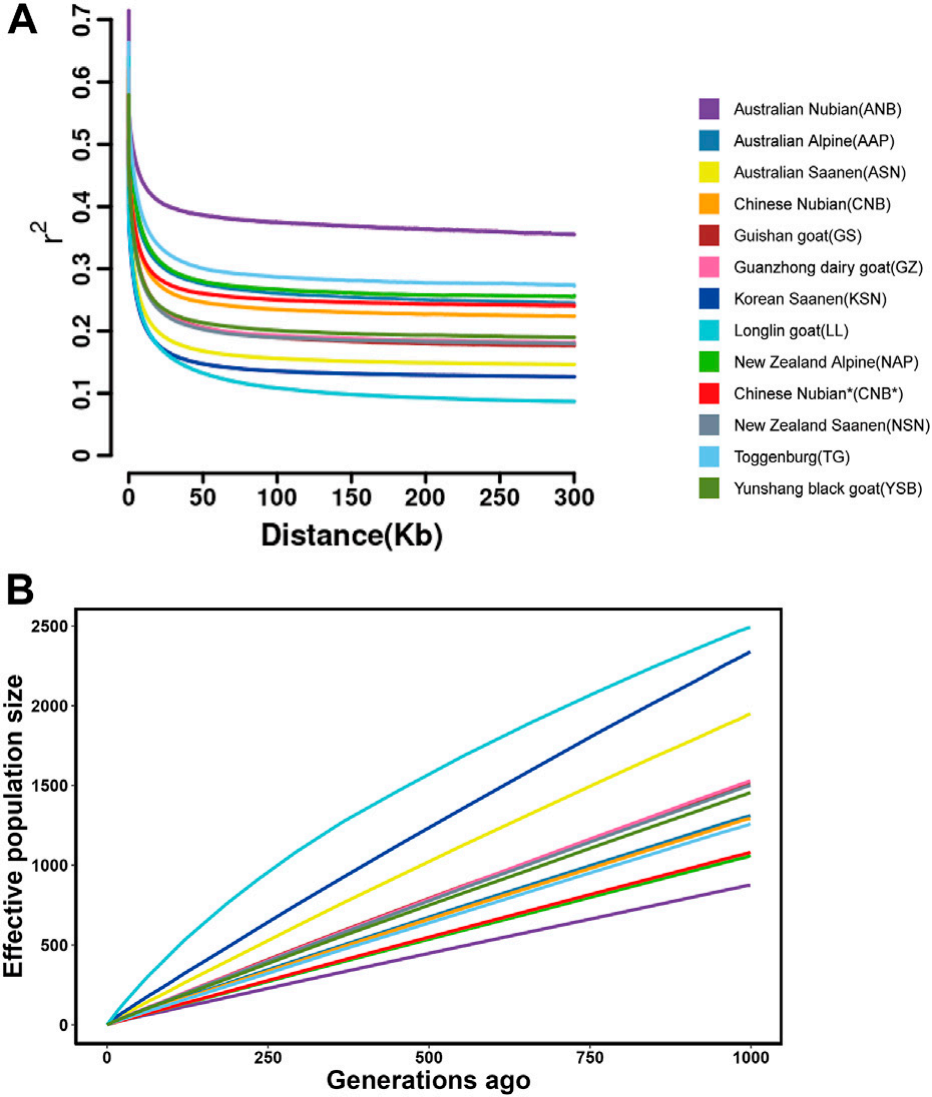
**(A)** Genome-wide average linkage disequilibrium decay in each population. **(B)** The effective population size of each population in recent 1,000 generations ago.

To evaluate the genetic diversity and genomic characteristics of the studied goat populations, we calculated the linkage disequilibrium decay (LD), nucleotide diversity (π), inbreeding coefficient, and effective population size (*Ne*) of the different goat populations. LD analysis showed that the average LD (r2) of all populations decreased rapidly at 0–50 kb and reached a plateau at around 200 kb. With distance markers of 300 kb, the average LD (r2) was highest in ANB, followed by TG, NAP, and AAP. However, the average LD (r2) of the LL was the lowest and decayed significantly faster than other populations (Figure 3A). Nucleotide diversity analysis showed that the average nucleotide diversity of ANB was the lowest and was extremely significantly lower than that of other goat populations (*p* < 0.01). However, the average nucleotide diversity of NSN was the highest, which was significantly higher than all goat populations except KSN and NAP (*p* < 0.01) (Table 2). In addition, we also calculated the average inbreeding coefficient (*F*_ROH_) of each goat population based on ROH (run of homozygosity). The results showed that the average inbreeding coefficient of AAP was the highest, which was significantly higher than that of all goat populations except TG and ANB (*p* < 0.01). The effective population size of 13 goat populations was predicted, and the results showed that the effective population size of LL was significantly higher than other goat populations, while ANB was significantly lower than the other goat populations (Figure 3B). At 1,000 generations ago, LL (2,491) was the highest, followed by the KSN (2,338) and ASN (1,950), and the ANB (874) the lowest.

**TABLE 2:** The summary of nucleotide diversity and inbreeding coefficient for 13 goat populations.

Population	Abbreviation	Nucleotide diversity	Inbreeding coefficient (*F*_ROH_)
		Mean ± SE^1^	Mean ± SE^1^
Australian Alpine	AAP	0.00142 ± 0.00000274^I^	0.204,257 ± 0.018853^A^
Toggenburg	TG	0.001449 ± 0.00000275^H^	0.16849 ± 0.014867^AB^
Australian Nubian	ANB	0.001203 ± 0.00000265^J^	0.16062 ± 0.032968^AB^
Guanzhong dairy goat	GZ	0.001692 ± 0.00000287^E^	0.136,827 ± 0.022484^BC^
Chinese Nubian	CNB	0.001676 ± 0.00000285^F^	0.131,692 ± 0.014115^BC^
Yunshang Black goat	YSB	0.001749 ± 0.00000291^C^	0.113,076 ± 0.011341B^CD^
New Zealand Alpine	NAP	0.001783 ± 0.00000304^AB^	0.100,103 ± 0.00728^CDE^
New Zealand Saanen	NSN	0.001785 ± 0.00000296^A^	0.093629 ± 0.007288^CDE^
Australian Saanen	ASN	0.001773 ± 0.00000293^B^	0.092642 ± 0.01102^CDE^
Korean Saanen	KSN	0.001779 ± 0.00000294^AB^	0.089476 ± 0.009852^CDE^
Longlin goat	LL	0.001545 ± 0.00000265^G^	0.084156 ± 0.005976^CDE^
Guishan goat	GS	0.001715 ± 0.00000289^D^	0.066048 ± 0.005825^DE^
Chinese Nubian*	CNB*	0.001752 ± 0.00000288^C^	0.041668 ± 0.008752^E^

Note: SE is the abbreviation of standard error. Different capital letters in the same column indicate significant differences between populations (*p* < 0.01), while the same letters indicate no significant differences.

*represents the data downloaded from NCBI. This is to distinguish it from our sequencing data.

### 3.4 Selective signals in dairy goat

**FIGURE 4:**
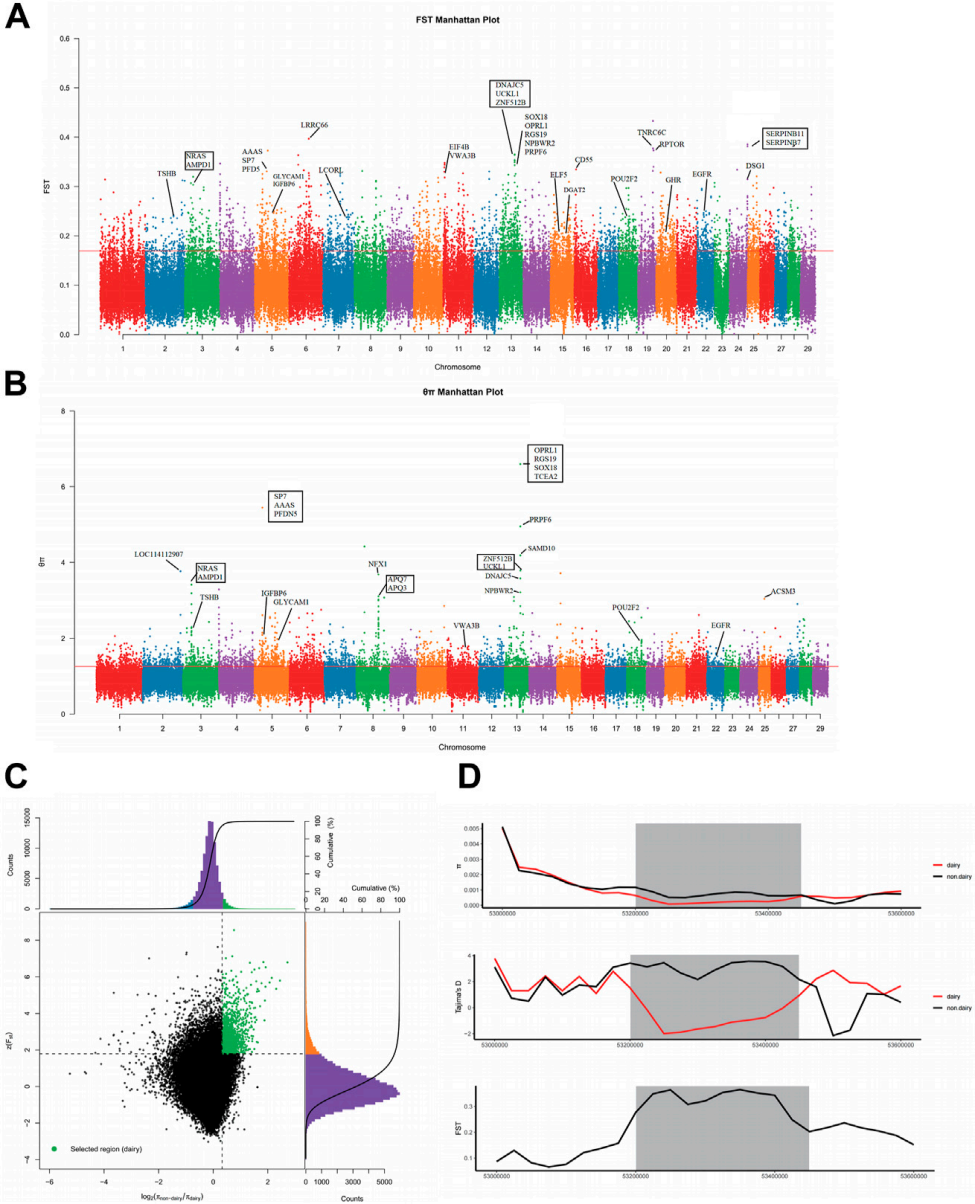
**(A)** Identification of selective signals (*F*_ST_ Manhattan plot). **(B)** Identification of selective signals (the θπ ratio Manhattan plot). **(C)** Distribution of log2 (θπ ratios) and *F*_ST_ values calculated in 50-kb sliding windows between dairy goat and non-dairy goat. **(D)** Selective sweep on chromosome 13 (53.2–53.4 Mb) (gray area).

To identify the specificity of dairy goat, we compared the genomes of dairy goats with those of non-dairy goats by calculating the ratio of nucleotide diversity (θπ_non-dairy/dairy_) and pairwise genetic differentiation (*F*_ST_) with a 50 kb sliding window and a 25 kb sliding step. The top 5% threshold outlier windows of the two methods were combined, which was considered to be the specific region of dairy goats under positive selection (*F*_ST_ = 0.17, θ_π_ ratio = 1.25) (Figures 4A, B). As a result, we identified a total of 1,315 genomic regions under selective sweep covering or being near to 876 candidate genes that were associated with dairy goat specificity traits (Figure 4C). Furthermore, we found that the region of 53.2–53.4 Mb in chromosome 13 showed an extreme *F*_ST_, a higher value of the ratio of θπ, and a negative value of Tajima’s D (Figure 4D). This suggests that this region may be associated with the breed-specificity of dairy goat. In this region, 16 candidate genes have been annotated, which are related to reproduction, immunity, growth and development traits.

The functional enrichment analysis of 876 selected candidate genes showed that 40 GO entries were significantly enriched (*p* < 0.05), such as protein binding, desmosome, regulation of the cellular metabolic process, regulation of the biological process, and protein catabolic process. In addition, 15 KEGG pathways were found to be significantly enriched (*p* < 0.05), including GnRH secretion, protein export, and the Hedgehog signaling pathway.

Through selection feature analysis, we found that some regions were strongly selected by positive selection. The candidate genes annotated in these regions included *LRRC66, PRPF6, EIF5B, NPBWR2, ZNF512B, AAAS, NRAS, SOX18, RGS19.* Based on gene function enrichment and references, we found that some candidate genes were associated with milk production traits, reproductive traits, and immune traits. In addition, we also found that some candidate genes were located on quantitative trait locus (QTLs) related to milk production traits in cattle (Table 3).

**TABLE 3:** Candidate genes located on the QTLs of bovine milk production traits.

Candidate gene	Gene description	Traits	References
*GHR*	Growth Hormone Receptor	Milk protein, milk yield, milk fat	Jiang et al. (2019b)
*GLYCAM1*	Glycosylation Dependent Cell Adhesion Molecule 1	Milk protein, milk yield, milk fat	Jiang et al. (2019b)
*GSK3A*	Glycogen Synthase Kinase 3	Milk protein, milk fat	Cole et al. (2011)
*PITRM1*	Pitrilysin Metallopeptidase 1	Milk protein, milk fat	Cole et al. (2011)
*APBB2*	Amyloid Beta Precursor Protein Binding Family B Member 2	Milk protein, milk fat	Cole et al. (2011)
*ARHGAP22*	Rho GTPase Activating Protein 22	Milk protein, milk yield, milk fat	Cole et al. (2011)
*LARP4B*	La Ribonucleoprotein 4B	Milk protein	Zhou et al. (2019)
*NCKAP1L*	NCK Associated Protein 1 Like	Milk protein, milk fat	Jiang et al. (2019b)
*TANK*	TRAF Family Member Associated NFKB Activator	Milk protein, milk yield, milk fat	Jiang et al. (2019b)
*AAAS*	Aladin WD Repeat Nucleoporin	Milk protein, milk fat	Jiang et al. (2019b)
*PRKDC*	Protein Kinase, DNA-Activated, Catalytic Subunit	Milk protein, milk yield	Jiang et al. (2019b)
*SPIDR*	Scaffold Protein Involved In DNA Repair	Milk protein	Jiang et al. (2019b)
*PDE1B*	Phosphodiesterase 1B	Milk protein	Jiang et al. (2019b)
*DGKG*	Diacylglycerol Kinase Gamma	Milk yield	Viale et al. (2017)
*TPD52L2*	TPD52 Like 2	Milk yield	Jiang et al. (2019b)
*RASAL1*	RAS Protein Activator Like 1	Milk yield	Do et al. (2020)
*SUPT3H*	SPT3 Homolog, SAGA And STAGA Complex Component	Milk yield	Jiang et al. (2019b)
*DNAJC5*	DnaJ Heat Shock Protein Family (Hsp40) Member C5	Milk yield	Jiang et al. (2019b)
*PRPF6*	Pre-MRNA Processing Factor 6	Milk yield	Jiang et al. (2019b)
*SPIDR*	Scaffold Protein Involved In DNA Repair	Milk yield	Jiang et al. (2019b)
*ABTB2*	Ankyrin Repeat And BTB Domain Containing 2	Milk fat	Jiang et al. (2019b)
*ACSBG2*	Acyl-CoA Synthetase Bubblegum Family Member 2	Milk fat	Jiang et al. (2019a)
*DNAJC24*	DnaJ Heat Shock Protein Family (Hsp40) Member C24	Milk fat	Jiang et al. (2019b)
*ATF7*	Activating Transcription Factor 7	Milk fat	Jiang et al. (2019b)
*FA2H*	Fatty Acid 2-Hydroxylase	Milk fat	Cole et al. (2011)
*NFX1*	Nuclear Transcription Factor, X-Box Binding 1	Milk fat	Jiang et al. (2019a)
*RFX4*	Regulatory Factor X4	Milk fat	Cole et al. (2011)
*STK3*	Serine/Threonine Kinase 3	Milk fat	Cai et al. (2019)
*UBE2R2*	Ubiquitin Conjugating Enzyme E2 R2	Milk fat	Jiang et al. (2019b)
*CUL3*	Cullin 3	Milk fat	Cole et al. (2011)
*RUNX3*	RUNX Family Transcription Factor 3	Milk fat	Jiang et al. (2019b)

## 4 Discussion

With human migration and the development of agricultural trade, dairy goats have been widely distributed all over the world, particularly in Europe and developing countries. They can adapt to harsh climates and living conditions, while contributing to the livelihood of low and middle-income farmers. Europe contains over 187 goat breeds, most of them are high-yield dairy goat breeds (Saanen, Alpine, Toggenburg, Nubian) (Devendra and Haenlein, 2016). However, in developing countries, the milk yield of some dairy goats is very low, which seriously restricts the economic development of developing countries. Therefore, exploring the genetic relationship of dairy goat breeds, revealing the specificity of dairy goat, and identifying candidate genes related to milk production traits will provide theoretical references for improving dairy goat and the implementation of effective crossbreeding programs. In this study, we investigated the genetic relationships and diversity and the signatures of selection in dairy goat populations based on the whole genome re-sequencing data of 89 goats (62 dairy goats and 27 non-dairy goats).

### 4.1 Population genetic structure and genetic diversity

Genetic diversity in livestock not only outlines the genomic characteristics of a population, but also indirectly reflects various evolutionary events. GS and LL are native to southwest China, and their geographical isolation is relatively weak. In fact, the analysis of, population structure shows that they have close genetic relationship. Due to the geographical location and living environment of LL, it has been in a relatively closed and natural mating state for a long time, with a resulting low intensity of artificial selection. From these results, we also found that LL had the fastest decay in LD, the effective population size was the largest, which was basically consistent with the results of previous studies (Chen et al., 2021). GZ and YSB are crossbred breeds with short breeding times, whose fathers are Saanen and Nubian, respectively. Thus, we found that YSB and Nubian, the GZ and Saanen had a close genetic relationship and similar genetic backgrounds which is consistent with the history of the formation of these breeds. Saanen, Alpine and Toggenburg belong to European dairy goat breeds, which are clustered into a large branch on the NJ tree, and show a similar genetic background.

In this study, we found that the nucleotide diversity of ANB, AAP, and TG was low, which was significantly lower than that of other populations, and the decline of LD was slow, indicating that these populations may have been strong artificial selection. And the results of LD decay of each population were largely consistent with the results of nucleotide diversity and inbreeding coefficient. In addition, genetic diversity showed that there were differences in nucleotide diversity, linkage disequilibrium, effective population size and inbreeding coefficient among Saanen, Alpine, and Nubian populations living in different countries. Kim et al. reported similar results in their study of the genomic characteristics of 14 goat populations, including Saanen (Korea, France, Switzerland, Australia) and Boer goat (Korea, Switzerland, Australia), wherein goats from different countries had differences in genomic characteristics (Kim et al., 2019). These differences in genetic diversity indicate that sub-populations may have been influenced by artificial or environmental selection in each region, thus resulting in different genomic characteristics. The results of this study are of great significance for exploring the genetic relationship among dairy goat breeds and provide a valuable reference for the improvement and cross-breeding of dairy goat breeds.

### 4.2 Selective sweep analysis

During long-term selection and domestication, humans have bred goat breeds with various production characteristics depending on their production needs. These characteristics have provided an important genetic basis for improving goat breeds and the design of breeding programs. Therefore, to identify these production characteristics, extensive studies on selection signatures have been carried out among various goat breeds: Bore goat (meat) (Yang et al., 2021a), large-tailed Han sheep (tail type) (Yuan et al., 2017), Lacaune sheep (milk) (Yuan et al., 2019), Laoshan dairy goat (reproductive) (Lai et al., 2016), and Inner Mongolia and Liaoning cashmere goats (cashmere) (Li et al., 2017). In the present study, we conducted comparative genomic analysis of five dairy goat breeds and three non-dairy goat breeds to reveal specific selection signatures for dairy goat.

In this study, we used the reference genome of dairy goat (Saanen_v1), with a genome alignment rate of over 99%. However, we found that the stronger selection sweeps regions had no annotated genes, and 202 annotated genes were found that could not be identified (no known orthologs, gene identifier starting with “LOC”). This may be due to the incomplete annotation of the caprine genome. Nevertheless, we still annotated candidate genes related to the specificity of dairy goat, such as milk production, reproduction, and immunity.

#### 4.2.1 Candidate genes associated with milk production traits

Milk production traits are not only the most important economic traits of dairy goat, but also their most significant characteristics. Milk production traits belong to quantitative traits, which are controlled by minor polygenes. The main indicators of these traits include milk yield, milk protein and fat yield, milk protein and fat content.

##### 4.2.1.1 Candidate genes associated with milk proteins

Milk proteins mainly include whey protein and casein, whose synthesis is regulated by a variety of hormones and signaling pathways, including insulin, and the JAK-STAT and mTOR signaling pathways. We identified several genes potentially involved in milk protein synthesis. Among these, *EIF4B* is key component of eukaryotic translation initiation and plays an important role in the mTOR signaling pathway, accelerating mRNA translation and promoting cell growth and proliferation (Cho et al., 2021). *ELF5* is an important regulator of mammary acinar development and milk protein expression, and its expression seems to enhance the activity of *STAT5* protein, which may be a major player in activating protein synthesis in the bovine mammary gland (Choi et al., 2009; Guan et al., 2020). IGFs (insulin-like growth factors) are essential for mammalian growth and development, and IGFBPs (IGF-binding proteins) are key regulators of IGF action (Bach et al., 2013). Previous studies have reported that *IGF2R* can affect milk quality and is an important genetic marker for milk production traits, while *IGFBP6* inhibits cell proliferation by specifically binding to *IGF2* (Dux et al., 2018; Nishihara et al., 2019).

##### 4.2.1.2 Candidate genes associated with milk fat

Milk fat is a high-quality natural fat that is an important component of milk, 99% of which consists of milk triglycerides (Bionaz and Loor, 2008). Milk triglycerides are mainly derived from mammary epithelial cells (MECs) and blood, and their synthesis and transfer involve several pathways, including the *de novo* synthesis of fatty acids, the formation of fat droplets, and the uptake and transport of fatty acids. Several genes have been reported to be involved in ruminant milk fat synthesis, including *EGFR*, *ACSS2*, *ACSL4*, *PANK3*, *DGAT2*, and *SP1*. Among these, *EGFR* plays an important role in the *de novo* synthesis of fatty acids in mammary epithelial cells by phosphorylating PLC-γ1 and Akt signaling pathways, inducing adipogenesis-related gene expression and increasing intracellular TG content (Huang et al., 2020). *ACSS2* (acyl-CoA synthase short-chain family member 2) performs lipogenesis by synthesizing acetyl-CoA from acetate, which is directly regulated by *SREBP1* and plays an important role in ruminant milk fat synthesis (Mu et al., 2021). *PANK3* is a key enzyme for fatty acid synthesis, which promotes *de novo* fatty acids synthesis by regulating CoA synthesis (Lin et al., 2013; Subramanian et al., 2016). *ACSL4* is a member of the acyl-CoA synthetase long-chain family, and plays a key role in lipid biosynthesis and fatty acid degradation by converting free long-chain fatty acids to fatty acyl-CoA (Mashek et al., 2007; Ren et al., 2022). *DGAT2* (diacylglycerol O-Acyltransferase 2) plays a key role in catalyzing the final step of triacylglycerol (TAG) biosynthesis in the Kennedy pathway and can significantly affect milk production and fat content in goats (Wakimoto et al., 2003; An et al., 2011). *SP1* (specificity protein 1) is one of the major transcription activators of FASN and may be involved in milk fat metabolism *via* the control of PPARγ and LXRα (Zhu et al., 2015).

Milk fat globular membrane (MFGM) is an important component of milk fat. *CHMP1B* belongs to MFGM protein and is highly or uniquely expressed in donkey mature milk (Chang et al., 2019; Li et al., 2019). *GlyCAM1* (glycosylation-dependent cell adhesion molecule 1) is one of the mucins produced in the mammary gland, which is a component of the milk fat globule membrane, and is highly and specifically expressed in the mammary glands of lactating bovines (Groenen et al., 1995; Le Provost et al., 2003).

Lipid droplets play an important role in triglyceride storage, lipid metabolism, and fatty acid transport. *PLIN4* (Perilipin4) and *PLIN5* (Perilipin5) are members of the Perilipin family of lipoproteins (Chen et al., 2013). *PLIN4* is involved in the formation of lipid droplets in adipocytes, and the binding of *PLIN5* to adipose triglyceride fatty acid enzyme (ATGL) plays a bidirectional regulatory role in lipid metabolism (Najt et al., 2020; Zhang et al., 2022). *ACSBG2* belongs to the acyl-CoA synthetase family, which plays a pivotal role in lipid metabolism and lipid droplet formation (Adams et al., 2016). *RPTOR* is a protein related to mTOR, which interacts with *S6K1* to regulate cell growth and plays a role in lipid droplet metabolism (Kim et al., 2002; Zhang et al., 2020).

##### 4.2.1.3 Candidate genes associated with milk production

In addition to this, we also identified several genes related to milk production. Mammary gland development is also an important factor affecting the lactation ability of livestock. Growth hormone plays an important role in mammary gland development (Tucker, 1981; Sejrsen et al., 1986), and *GHR* (growth hormone receptor) activates mammary gland cell activity, which can significantly affect milk production in buffaloes (El-Komy et al., 2020), dairy cows (Cobanoglu et al., 2021), and sheep (Dettori et al., 2018). *LCORL* plays an important role in regulating the body size and height of livestock, and previous studies have found that *LCORL* is associated with milk yield in Latxa sheep, which may affect breast development (Ruiz-Larrañaga et al., 2018; Saif et al., 2020). *CPXM2* may play an important role in mammary gland development and involution (Suárez-Vega et al., 2015). Mammary epithelial cells have the function of synthesizing and secreting milk. As such, the genes involved in cell proliferation and differentiation may indirectly affect milk production. *CCR6* (chemokine receptor 6) activates multiple signaling kinases and participates in CCL20-induced breast cell proliferation and migration (Marsigliante et al., 2013). *NRAS* plays an important role in cell proliferation, cell migration, and angiogenesis by activating the PI3K/AKT, MAPK/ERK, and NF-kB signaling pathways (Song et al., 2018). *SP7* is a zinc finger transcription factor, which promotes the proliferation and differentiation of osteoblasts and regulates the expression of several osteoblast genes, such as *SPP1* and *BMP2* (Barbuto and Mitchell, 2013; Hojo and Ohba, 2022). *AMPD1* plays an important role in cellular energy metabolism, which is a candidate gene for bovine production traits and porcine growth and carcass traits (Wang et al., 2008; Wei et al., 2015).

#### 4.2.2 Candidate genes associated with reproduction traits

Dairy goat are seasonal breeding livestock within which the hypothalamic-pituitary-thyroid (HPT) axis plays a crucial role in sexual maturation and reproduction (Müller-Fielitz et al., 2017). *TSHB* is an important component of thyroid stimulating hormone (TSH), deciding the biological specificity and synthesis rate of TSH, and is closely related to the photoperiod of seasonal reproduction in mammals (Huang et al., 2013). *TSHR* is a thyroid-stimulating hormone receptor that plays an important role in seasonal estrus and reproduction in goats (Yurchenko et al., 2019; Li et al., 2021a). *PTGS2*, *ESR2*, and *SERPINB11* are relevant to estrogen and follicular growth (Xu et al., 2018). *PTGS2* plays a critical role in ovulation by stimulating LH (luteinizing hormone) signaling (Tang et al., 2017) Estrogen receptor 2 (*ESR2*) plays a critical role in folliculogenesis and ovulation by regulating granulosa cells (Khristi et al., 2018). *SERPINB11* is a novel estrogen-inducible gene during chicken fallopian tube development that may be involved in spermatogenesis and apoptosis (Lim et al., 2012; Yang et al., 2015). *SPATA22* (spermatogenesis associated 22) was identified as a candidate meiosis-specific gene and plays a crucial role in sperm and oocyte meiosis (Lawson et al., 2011; La Salle et al., 2012). *CHMP5* is essential for embryogenesis, and the lack of this gene leads to early embryonic lethality in mice (Shim et al., 2006). Furthermore, previous studies have shown that *MSRA* is related to productivity traits of Romanov sheep, while *AAAS* is associated with reproductive traits in Iranian sheep (Xu et al., 2018; Manzari et al., 2019).

#### 4.2.3 Candidate genes related to immunity

Intramammary infections and mastitis lead to reduced milk production and quality in dairy goats. In this study, we identified several immune-related genes. *JAK1* is a key effector of proinflammatory cytokine signaling, plays an important role in immune function, and can promote the self-renewal of hematopoietic stem cells (Kleppe et al., 2017; Witalisz-Siepracka et al., 2018). *BPIFA1* and *BPIFA3* belong to the BPI fold-containing family, which have antibacterial, surfactant, and immunomodulatory properties that prevent bacterial biofilm formation (Britto and Cohn, 2015; Guan et al., 2020). *POU2F2* regulates B cell proliferation and differentiation genes by binding to immune proteins to participate in immune responses (Yang et al., 2021c). *LRRC66* is a key gene for insulin and plays an important role in the development of innate immunity and the nervous system, which is involved in diverse biological processes, including cell adhesion, cellular trafficking, and hormone-receptor interactions (Ng et al., 2011; Lien et al., 2020). *MAP3K1* participates in the MAPK signaling pathway and can inhibit the proliferation of breast cancer cells, playing a vital role in maintaining good breast function (Cuevas et al., 2006; Glubb et al., 2015).

#### 4.2.4 Candidate genes associated with the strongest selection signal

In addition, we found that the 53.2–53.4 Mb region on chromosome 13 was strongly positively selected, and the enriched genes therein were mainly related to reproduction, immunity, growth and development (Table 4). *NPBWR2*, *PRPF6*, *OPRL1*, and *SOX18* play essential roles in the germ cells of livestock, while *DNAJC5* and *ZNF512B* play important roles in growth and development, which may be related to the development of mammary glands. In addition, *PRPF6* and *DNAJC5* are associated with milk production in cows (Jiang et al., 2019b).

**TABLE 4:** Genes annotated in the 53.2–53.4 Mb region of chromosome 13.

Gene	CHROM	BIN_START	BIN_END	*F*_ST_	θπ
*NPBWR2*	13	53225001	53275000	0.349	2.848
*OPRL1*	13	53250001	53300000	0.364	6.597
*LKAAEAR1*	13	53250001	53300000	0.364	6.597
*RGS19*	13	53250001	53300000	0.364	6.597
*TCEA2*	13	53250001	53300000	0.364	6.597
*SOX18*	13	53250001	53300000	0.364	6.597
*C13H20orf204*	13	53300001	53350000	0.321	4.189
*PRPF6*	13	53300001	53350000	0.321	4.189
*LOC114117706*	13	53300001	53350000	0.321	4.189
*SAMD10*	13	53300001	53350000	0.321	4.189
*LOC114117706*	13	53325001	53375000	0.354	3.788
*ZNF512B*	13	53325001	53375000	0.354	3.788
*LOC114117598*	13	53325001	53375000	0.354	3.788
*UCKL1*	13	53325001	53375000	0.354	3.788
*LOC114117599*	13	53350001	53400000	0.365	3.577
*DNAJC5*	13	53350001	53400000	0.365	3.577

*NPBWR2* regulates the expression of PRL and GH by mediating neuropeptide W and may play a role in the reproductive activity of pigs (Scanes, 2016; Yang et al., 2018). *PRPF6* is a key regulator of androgen receptor (AR), interacts with the N-terminus of AR, and enhances AR-mediated transactivation (Liu et al., 2021). *OPRL1* plays an important role in spermatogenesis and meiosis through follicle-stimulating hormone (FSH) (Eto, 2015). *SOX18* is an important regulator of vascular development, which is essential in angiogenesis and vascular endothelial cell differentiation (Fontijn et al., 2014). It is also reported to be a specific marker gene for sheep germ cells (Yang et al., 2021b). Puberty is an important period for the growth and development of the body. In this context, *DNAJC5* and *ZNF512B* are developmental regulators and play a role in the puberty development of goats (Yan et al., 2022) and cattle (Nguyen et al., 2017). *RGS19* promotes cell proliferation and differentiation, and plays a role in immune processes by regulating the survival of macrophages through the Notch signaling pathway (Sangphech et al., 2014). *UCKL1* is a uridine-cytidine kinase that plays a role in cell proliferation and survival and is highly expressed in breast cancer (Ambrose and Kornbluth, 2009; Kovalevska et al., 2021).

## 5 Conclusion

In this study, the whole-genome resequencing data was used to analyze the population structure and genetic diversity of dairy goats. As a result, the genetic relationships of different dairy goat were revealed. Our results confirmed that differences exist in the genomic characteristics of the Nubian, Alpine, and Saanen subpopulations in different regions. In addition, we identified dairy goat-specific traits and candidate genes associated with milk production, reproductive, and immune traits. Furthemore, we found that chromosome 13 was strongly selected at 53.2–53.4 Mb, which may be a region-specific to dairy goat. We need more studies to verify. To summarized, our findings provide insights into the genetic diversity, population evolution, and important economic traits of dairy goat, as well as a valuable reference for the improvement and development of cross-breeding programs for dairy goat breeds.

## Data Availability

The raw sequence data reported in this paper have been deposited in the Genome Sequence Archive (Genomics, Proteomics and Bioinformatics 2021) in National Genomics Data Center (Nucleic Acids Res 2022), China National Center for Bioinformation/Beijing Institute of Genomics, Chinese Academy of Sciences (GSA: CRA008399) that are publicly accessible at https://ngdc.cncb.ac.cn/gsa.
