# Supplementary material for: Whole-genome resequencing reveals genetic diversity and selection characteristics of dairy goat
Source: Front Genet. 2023 Jan 6;13:1044017. doi: 10.3389/fgene.2022.1044017 (PMC9852865; doi:10.3389/fgene.2022.1044017)
Supplement: Supplementary file 1 [file DataSheet1.docx]

***Supplementary Material***

**Whole-genome resequencing reveals genetic diversity and selection characteristics of dairy goat**

**Article type:** ORIGINAL RESEARCH

**Supplementary information includes:**

**Table S1.** The data statistic for 48 re-sequenced goat samples.

**Table S2.** The data statistic for 41 publicly available goats.

**Table S3.** The summary of nucleotide diversity and inbreeding coefficient for 13 goat populations

**Table S4.** The summary of inbreeding coefficient for 89 goats

**Table S5.** Candidate regions and genes under positive selection in dairy goat.

**Table S6.** Significantly enriched GO entries (*P*< 0.05)

**Table S7.** Significantly enriched KEGG pathways (*P*< 0.05)

**Figure 1** Functional annotation of the identified SNPs in goat breeds

**Figure 2** The Cross-validation plot for admixture analysis.

**Figure 3** (A) The boxplot indicates the distribution of nucleotide diversity of each breed in 50-Kb windows with 25-Kb steps. (B) Inbreeding coefficient for each breed

**Figure 4** Gene Ontology (GO) entries

**Figure 5** KEGG pathway

**Figure 6** Significantly enriched KEGG pathways

**Table S1.** The data statistic for 48 re-sequenced goat samples.

| **Population** | **Abbreviation** | **Sample ID** | **BioSample** | **Clean_**  **reads** | **mapped_**  **reads** | **Alignment**  **Rate (%)** | | **Read**  **Depth** | | **Genome**  **Coverage** | **Longitude** | | **Latitude** |
| --- | --- | --- | --- | --- | --- | --- | --- | --- | --- | --- | --- | --- | --- |
| Guishan goat | GS | 1 | SAMC911838 | 651707534 | 649586834 | | 0.9967 | | 29.92 | 0.993 | | 103.29 | 24.77 |
| Guishan goat | GS | 2 | SAMC911839 | 686085582 | 683623424 | | 0.9964 | | 30.9 | 0.9929 | | 103.29 | 24.77 |
| Guishan goat | GS | 3 | SAMC911840 | 686584016 | 684316067 | | 0.9967 | | 30.8 | 0.9924 | | 103.29 | 24.77 |
| Guishan goat | GS | 4 | SAMC911841 | 608538584 | 606402616 | | 0.9965 | | 28.83 | 0.9891 | | 103.29 | 24.77 |
| Guishan goat | GS | 5 | SAMC911842 | 638418960 | 636491968 | | 0.997 | | 30.43 | 0.9893 | | 103.29 | 24.77 |
| Guishan goat | GS | 6 | SAMC911843 | 674095188 | 671849296 | | 0.9967 | | 30.56 | 0.9886 | | 103.29 | 24.77 |
| Yunshang black goat | YSB | 7 | SAMC911844 | 779604662 | 777192015 | | 0.9969 | | 35.55 | 0.9888 | | 102.82 | 24.89 |
| Yunshang black goat | YSB | 8 | SAMC911845 | 633151102 | 631452326 | | 0.9973 | | 28.99 | 0.9888 | | 102.82 | 24.89 |
| Yunshang black goat | YSB | 9 | SAMC911846 | 771662664 | 769493773 | | 0.9972 | | 34.81 | 0.9888 | | 102.82 | 24.89 |
| Yunshang black goat | YSB | 10 | SAMC911847 | 773909674 | 771754592 | | 0.9972 | | 35.74 | 0.9921 | | 102.82 | 24.89 |
| Yunshang black goat | YSB | 11 | SAMC911848 | 728660614 | 726313992 | | 0.9968 | | 33.85 | 0.9922 | | 102.82 | 24.89 |
| Yunshang black goat | YSB | 12 | SAMC911849 | 640123852 | 636910953 | | 0.995 | | 30.27 | 0.9923 | | 102.82 | 24.89 |
| Chinese Nubian | CNB | 13 | SAMC911850 | 714164912 | 711997057 | | 0.997 | | 33.36 | 0.9889 | | 102.82 | 24.89 |
| Chinese Nubian | CNB | 14 | SAMC911851 | 741243510 | 738916745 | | 0.9969 | | 35.07 | 0.989 | | 102.82 | 24.89 |
| Chinese Nubian | CNB | 15 | SAMC911852 | 840393170 | 837405326 | | 0.9964 | | 38.64 | 0.9894 | | 102.82 | 24.89 |
| Chinese Nubian | CNB | 16 | SAMC911853 | 579796994 | 577883911 | | 0.9967 | | 28.05 | 0.9919 | | 102.82 | 24.89 |
| Chinese Nubian | CNB | 17 | SAMC911854 | 731934988 | 729508067 | | 0.9967 | | 33.62 | 0.9919 | | 102.82 | 24.89 |
| Chinese Nubian | CNB | 18 | SAMC911855 | 663739722 | 661261213 | | 0.9963 | | 30.68 | 0.9923 | | 102.82 | 24.89 |
| Toggenburg | TG | 19 | SAMC911856 | 697854392 | 695774260 | | 0.997 | | 31.56 | 0.9938 | | 102.82 | 24.89 |
| Toggenburg | TG | 20 | SAMC911857 | 656112640 | 654227021 | | 0.9971 | | 29.76 | 0.9933 | | 102.82 | 24.89 |
| Toggenburg | TG | 21 | SAMC911858 | 700705246 | 698673765 | | 0.9971 | | 32.22 | 0.9936 | | 102.82 | 24.89 |
| Toggenburg | TG | 22 | SAMC911859 | 645438600 | 643540456 | | 0.9971 | | 29.88 | 0.9897 | | 102.82 | 24.89 |
| Toggenburg | TG | 23 | SAMC911860 | 655806330 | 653954702 | | 0.9972 | | 30.43 | 0.9899 | | 102.82 | 24.89 |
| Toggenburg | TG | 24 | SAMC911861 | 599516836 | 597767454 | | 0.9971 | | 27.78 | 0.9898 | | 102.82 | 24.89 |
| New Zealand Saanen | NSN | 205 | SAMC911869 | 640782686 | 638228273 | | 0.996 | | 30.03 | 0.9932 | | 109.59 | 34.62 |
| New Zealand Alpine | NAP | 427 | SAMC911863 | 600480946 | 598776207 | | 0.9972 | | 27.85 | 0.9931 | | 109.59 | 34.62 |
| New Zealand Saanen | NSN | 456 | SAMC911868 | 625682222 | 623710073 | | 0.9968 | | 28.67 | 0.9927 | | 109.59 | 34.62 |
| New Zealand Saanen | NSN | 516 | SAMC911870 | 594761468 | 592366460 | | 0.996 | | 28.46 | 0.9933 | | 109.59 | 34.62 |
| New Zealand Alpine | NAP | 520 | SAMC911866 | 596977446 | 595356690 | | 0.9973 | | 28.01 | 0.9894 | | 109.59 | 34.62 |

**Table S1.** The data statistic for 48 re-sequenced goat samples.

| **Population** | **Abbreviation** | **Sample**  **ID** | **BioSample** | **Clean_reads** | **mapped_**  **reads** | **Alignment**  **Rate (%)** | | **Read**  **Depth** | **Genome**  **Coverage** | | **Longitude** | | **Latitude** | |
| --- | --- | --- | --- | --- | --- | --- | --- | --- | --- | --- | --- | --- | --- | --- |
| Australian Saanen | ASN | 539 | SAMC911878 | 598182424 | 596101188 | | 0.9965 | 28.78 | | 0.9898 | | 109.59 | | 34.62 |
| New Zealand Alpine | NAP | 622 | SAMC911867 | 717072066 | 715013058 | | 0.9971 | 33.06 | | 0.9898 | | 109.59 | | 34.62 |
| New Zealand Alpine | NAP | 645 | SAMC911865 | 832887550 | 830277061 | | 0.9969 | 36.29 | | 0.9901 | | 109.59 | | 34.62 |
| Australian Saanen | ASN | 1402 | SAMC911876 | 652843654 | 650361471 | | 0.9962 | 31.08 | | 0.9936 | | 109.59 | | 34.62 |
| New Zealand Saanen | NSN | 1639 | SAMC911873 | 734110110 | 732085382 | | 0.9972 | 32.72 | | 0.989 | | 109.59 | | 34.62 |
| New Zealand Saanen | NSN | 1872 | SAMC911871 | 692489488 | 690297136 | | 0.9968 | 32.09 | | 0.9898 | | 109.59 | | 34.62 |
| New Zealand Saanen | NSN | 1943 | SAMC911872 | 717150236 | 714699158 | | 0.9966 | 32.94 | | 0.99 | | 109.59 | | 34.62 |
| Australian Saanen | ASN | 3218 | SAMC911877 | 642359650 | 639922499 | | 0.9962 | 29.62 | | 0.9897 | | 109.59 | | 34.62 |
| Australian Saanen | ASN | 3284 | SAMC911879 | 639721568 | 637771822 | | 0.997 | 30.04 | | 0.9897 | | 109.59 | | 34.62 |
| Australian Saanen | ASN | 3681 | SAMC911875 | 645947504 | 643648698 | | 0.9964 | 30.56 | | 0.9936 | | 109.59 | | 34.62 |
| Australian Saanen | ASN | 3690 | SAMC911874 | 681358468 | 678980513 | | 0.9965 | 31 | | 0.9932 | | 109.59 | | 34.62 |
| Guanzhong dairy goat | GZ | 17096 | SAMC911884 | 716440944 | 714179948 | | 0.9968 | 32.81 | | 0.9898 | | 109.59 | | 34.62 |
| Guanzhong dairy goat | GZ | 17108 | SAMC911883 | 702847866 | 700685613 | | 0.9969 | 32.21 | | 0.9896 | | 109.59 | | 34.62 |
| Guanzhong dairy goat | GZ | 156811 | SAMC911885 | 582145470 | 580166429 | | 0.9966 | 28.16 | | 0.9891 | | 109.59 | | 34.62 |
| Guanzhong dairy goat | GZ | 156833 | SAMC911880 | 634991102 | 632844632 | | 0.9966 | 30.11 | | 0.9925 | | 109.59 | | 34.62 |
| Guanzhong dairy goat | GZ | 156840 | SAMC911881 | 713028306 | 710823476 | | 0.9969 | 32.22 | | 0.9933 | | 109.59 | | 34.62 |
| Guanzhong dairy goat | GZ | 156843 | SAMC911882 | 626934858 | 625082431 | | 0.997 | 28.78 | | 0.9935 | | 109.59 | | 34.62 |
| Australian Alpine | AAP | B716 | SAMC911862 | 683513428 | 681245650 | | 0.9967 | 30.8 | | 0.9928 | | 109.59 | | 34.62 |
| Australian Alpine | AAP | B717 | SAMC911864 | 609210712 | 607464199 | | 0.9971 | 27.25 | | 0.9927 | | 109.59 | | 34.62 |

Note: The raw sequence data reported in this paper have been deposited in the Genome Sequence Archive (Genomics, Proteomics & Bioinformatics 2021) in National Genomics Data Center (Nucleic Acids Res 2022), China National Center for Bioinformation / Beijing Institute of Genomics, Chinese Academy of Sciences (GSA: CRA008399) that are publicly accessible at <https://ngdc.cncb.ac.cn/gsa>.

**Table S2.** The data statistic for 41 publicly available goats.

| **Population** | **Abbreviation** | **Sample ID** | **SRA Sample ID** | **Clean_reads** | **mapped_reads** | **Alignment**  **Rate (%)** | **Read**  **Depth** | **Genome**  **Coverage (%)** | **Longitude** | **Latitude** |
| --- | --- | --- | --- | --- | --- | --- | --- | --- | --- | --- |
| LongLin goat | LL | L1 | SRS6618860 | 253926126 | 253049858 | 0.9965 | 11.53 | 0.9868 | 105.34 | 24.77 |
| LongLin goat | LL | L2 | SRS6618861 | 238262558 | 236988975 | 0.9947 | 11.14 | 0.9871 | 105.34 | 24.77 |
| LongLin goat | LL | L3 | SRS6618867 | 287021252 | 285368630 | 0.9942 | 14.68 | 0.9805 | 105.34 | 24.77 |
| LongLin goat | LL | L4 | SRS6618868 | 250569126 | 249640869 | 0.9963 | 11.31 | 0.9873 | 105.34 | 24.77 |
| LongLin goat | LL | L5 | SRS6618869 | 264234242 | 263319083 | 0.9965 | 12.26 | 0.9875 | 105.34 | 24.77 |
| LongLin goat | LL | L606 | SRS6618870 | 325541488 | 324330834 | 0.9963 | 14.41 | 0.9875 | 105.34 | 24.77 |
| LongLin goat | LL | L607 | SRS6618871 | 231858114 | 230894756 | 0.9958 | 11.36 | 0.9847 | 105.34 | 24.77 |
| LongLin goat | LL | L608 | SRS6618872 | 226619068 | 225474337 | 0.9949 | 10.93 | 0.9889 | 105.34 | 24.77 |
| LongLin goat | LL | L609 | SRS6618873 | 289873482 | 288772277 | 0.9962 | 13.41 | 0.9894 | 105.34 | 24.77 |
| LongLin goat | LL | L610 | SRS6618874 | 255371364 | 253700461 | 0.9935 | 11.92 | 0.9909 | 105.34 | 24.77 |
| LongLin goat | LL | L720 | SRS6618862 | 359053544 | 357749076 | 0.9964 | 16.68 | 0.9917 | 105.34 | 24.77 |
| LongLin goat | LL | L721 | SRS6618863 | 281321312 | 279948457 | 0.9951 | 12.77 | 0.9901 | 105.34 | 24.77 |
| LongLin goat | LL | L722 | SRS6618864 | 226044420 | 224771983 | 0.9944 | 11.75 | 0.9851 | 105.34 | 24.77 |
| LongLin goat | LL | L723 | SRS6618865 | 210541132 | 209935164 | 0.9971 | 10.01 | 0.9897 | 105.34 | 24.77 |
| LongLin goat | LL | L724 | SRS6618866 | 210085664 | 209527004 | 0.9973 | 10.06 | 0.9896 | 105.34 | 24.77 |
| Korean Saanen | KSN | H230 | ERS2212977 | 411192814 | 410861885 | 0.9992 | 15.08 | 0.9871 | 125.58 | 37.33 |
| Korean Saanen | KSN | H231 | ERS2212978 | 389812144 | 389488252 | 0.9992 | 14.49 | 0.9873 | 125.58 | 37.33 |
| Korean Saanen | KSN | H232 | ERS2212979 | 370899944 | 370631703 | 0.9993 | 13.63 | 0.9863 | 125.58 | 37.33 |
| Korean Saanen | KSN | H233 | ERS2212980 | 406222436 | 405900803 | 0.9992 | 14.8 | 0.9903 | 125.58 | 37.33 |
| Korean Saanen | KSN | H234 | ERS2212981 | 358333458 | 358044288 | 0.9992 | 12.81 | 0.9895 | 125.58 | 37.33 |
| Korean Saanen | KSN | H235 | ERS2212982 | 375127514 | 374835397 | 0.9992 | 13.89 | 0.9839 | 125.58 | 37.33 |
| Korean Saanen | KSN | H236 | ERS2212983 | 369598450 | 369296851 | 0.9992 | 13.73 | 0.9843 | 125.58 | 37.33 |
| Korean Saanen | KSN | H237 | ERS2212984 | 367221542 | 366974703 | 0.9993 | 13.69 | 0.9874 | 125.58 | 37.33 |
| Korean Saanen | KSN | H238 | ERS2212985 | 353239862 | 352992808 | 0.9993 | 13.18 | 0.9887 | 125.58 | 37.33 |
| Korean Saanen | KSN | H239 | ERS2212986 | 387753376 | 387311120 | 0.9989 | 14.15 | 0.9902 | 125.58 | 37.33 |
| Australian Nubian | ANB | N1 | ERS2212970 | 311773628 | 310354445 | 0.9954 | 11.81 | 0.988 | 148.08 | -35.17 |
| Australian Nubian | ANB | N2 | ERS2212969 | 330067570 | 328833587 | 0.9963 | 12.18 | 0.9885 | 148.08 | -35.17 |
| Australian Nubian | ANB | N3 | ERS2212968 | 352816224 | 351214118 | 0.9955 | 13.06 | 0.9919 | 148.08 | -35.17 |
| Australian Nubian | ANB | N4 | ERS2212967 | 342100564 | 340565310 | 0.9955 | 12.72 | 0.9891 | 148.08 | -35.17 |
| Australian Nubian | ANB | N5 | ERS2212966 | 346857164 | 345658957 | 0.9965 | 12.78 | 0.9891 | 148.08 | -35.17 |

**Table S2.** The data statistic for 41 publicly available goats.

| **Population** | **Abbreviation** | **Sample**  **ID** | **SRA Sample**  **ID** | **Clean_reads** | **mapped_reads** | **Alignment**  **Rate (%)** | **Read**  **Depth** | **Genome**  **Coverage (%)** | **Longitude** | **Latitude** |
| --- | --- | --- | --- | --- | --- | --- | --- | --- | --- | --- |
| Australian Alpine* | AAP* | A1 | ERS2212961 | 306775410 | 305995198 | 0.9975 | 11.33 | 0.9922 | 148.08 | -35.17 |
| Australian Alpine* | AAP* | A2 | ERS2212962 | 324278440 | 323556024 | 0.9978 | 11.91 | 0.989 | 148.08 | -35.17 |
| Australian Alpine* | AAP* | A3 | ERS2212963 | 316798520 | 313530001 | 0.9897 | 11.83 | 0.9921 | 148.08 | -35.17 |
| Australian Alpine* | AAP* | A4 | ERS2212964 | 326258404 | 324837295 | 0.9956 | 12.09 | 0.9893 | 148.08 | -35.17 |
| Australian Alpine* | AAP* | A5 | ERS2212965 | 358168208 | 356643330 | 0.9957 | 13.16 | 0.9901 | 148.08 | -35.17 |
| Australian Saanen* | ASN* | B1 | ERS2212959 | 323048276 | 321789964 | 0.9961 | 11.98 | 0.9897 | 148.08 | -35.17 |
| Australian Saanen* | ASN* | B2 | ERS2212960 | 310361184 | 305995588 | 0.9859 | 11.45 | 0.9922 | 148.08 | -35.17 |
| Chinese Nubian* | CNB* | N10 | SRS4068201 | 216516378 | 215988076 | 0.9976 | 8.3 | 0.9791 | 148.08 | -35.17 |
| Chinese Nubian* | CNB* | N11 | SRS4068200 | 243525966 | 242785716 | 0.997 | 9.94 | 0.9813 | 148.08 | -35.17 |
| Chinese Nubian* | CNB* | N12 | SRS4068203 | 287843970 | 286539243 | 0.9955 | 11.44 | 0.9855 | 148.08 | -35.17 |
| Chinese Nubian* | CNB* | N13 | SRS4068204 | 293922714 | 293083858 | 0.9971 | 10.79 | 0.9854 | 148.08 | -35.17 |

**Table S3.** The summary of nucleotide diversity and inbreeding coefficient for 13 goat populations

| **Group** | **Abbreviation** | | **Nucleotide diversity** | | ***F*_ROH_** | |
| --- | --- | --- | --- | --- | --- | --- |
|  |  |  | **Mean** | **Median** | **Mean** | **Median** |
| Australian Nubian | | ANB | 0.001203 | 0.001044 | 0.16062 | 0.185681 |
| Australian Alpine | | AAP | 0.00142 | 0.001254 | 0.204257 | 0.204686 |
| Toggenburg | | TG | 0.001449 | 0.001278 | 0.16849 | 0.161685 |
| Longlin goat | | LL | 0.001545 | 0.001391 | 0.084156 | 0.078566 |
| Chinese Nubian | | CNB | 0.001676 | 0.001503 | 0.131692 | 0.13068 |
| Guanzhong dairy goat | | GZ | 0.001692 | 0.001508 | 0.136827 | 0.136496 |
| Guishan goat | | GS | 0.001715 | 0.001542 | 0.066048 | 0.068586 |
| Yunshang black goat | | YSB | 0.001749 | 0.001573 | 0.113076 | 0.114168 |
| Chinese Nubian* | | CNB* | 0.001752 | 0.001583 | 0.041668 | 0.041481 |
| Australian Saanen | | ASN | 0.001773 | 0.001593 | 0.092642 | 0.088001 |
| New Zealand Alpine | | NAP | 0.001783 | 0.001596 | 0.100103 | 0.100693 |
| Korean Saanen | | KSN | 0.001779 | 0.001598 | 0.089476 | 0.082892 |
| New Zealand Saanen | | NSN | 0.001785 | 0.001602 | 0.093629 | 0.091186 |

**Table S4.** The summary of inbreeding coefficient for 89 goats

| **Breed** | **Sample ID** | **ROH(KB)** | ***F*_ROH_** |
| --- | --- | --- | --- |
| Australian Alpine | A1 | 508779 | 0.204686 |
| Australian Alpine | A2 | 626998 | 0.252246 |
| Australian Alpine | A3 | 316580 | 0.127363 |
| Australian Alpine | A4 | 397611 | 0.159962 |
| Australian Alpine | A5 | 459488 | 0.184856 |
| Australian Alpine | B716 | 642061 | 0.258306 |
| Australian Alpine | B717 | 602477 | 0.242381 |
| Australian Nubian | N1 | 152001 | 0.0611512 |
| Australian Nubian | N2 | 585617 | 0.235598 |
| Australian Nubian | N3 | 461539 | 0.185681 |
| Australian Nubian | N4 | 267089 | 0.107452 |
| Australian Nubian | N5 | 529990 | 0.213219 |
| Australian Saanen | 1402 | 233242 | 0.0938351 |
| Australian Saanen | 3218 | 266825 | 0.107346 |
| Australian Saanen | 3284 | 224978 | 0.0905104 |
| Australian Saanen | 3681 | 212504 | 0.085492 |
| Australian Saanen | 3690 | 183139 | 0.0736783 |
| Australian Saanen | 539 | 394148 | 0.158569 |
| Australian Saanen | B1 | 129147 | 0.0519569 |
| Australian Saanen | B2 | 198224 | 0.0797471 |
| Chinese Nubian | 13 | 477843 | 0.19224 |
| Chinese Nubian | 14 | 312063 | 0.125545 |
| Chinese Nubian | 15 | 222172 | 0.0893816 |
| Chinese Nubian | 16 | 275032 | 0.110648 |
| Chinese Nubian | 17 | 339346 | 0.136522 |
| Chinese Nubian | 18 | 337590 | 0.135815 |
| Guishan goat | 1 | 115315 | 0.0463921 |
| Guishan goat | 2 | 214393 | 0.086252 |
| Guishan goat | 3 | 165926 | 0.0667533 |
| Guishan goat | 4 | 175036 | 0.0704184 |
| Guishan goat | 5 | 181273 | 0.0729276 |
| Guishan goat | 6 | 133093 | 0.0535444 |
| Guanzhong dairy goat | 156811 | 582004 | 0.234145 |
| Guanzhong dairy goat | 156833 | 347507 | 0.139805 |
| Guanzhong dairy goat | 156840 | 359554 | 0.144651 |
| Guanzhong dairy goat | 156843 | 188432 | 0.0758077 |
| Guanzhong dairy goat | 17096 | 232081 | 0.093368 |
| Guanzhong dairy goat | 17108 | 331055 | 0.133186 |
| Korean Saanen | H230 | 120150 | 0.0483373 |
| Korean Saanen | H231 | 216677 | 0.0871709 |
| Korean Saanen | H232 | 416404 | 0.167523 |
| Korean Saanen | H233 | 195375 | 0.0786009 |
| Korean Saanen | H234 | 188422 | 0.0758037 |
| Korean Saanen | H235 | 179862 | 0.0723599 |
| Korean Saanen | H236 | 201876 | 0.0812163 |
| Korean Saanen | H237 | 210205 | 0.0845671 |
| Korean Saanen | H238 | 234982 | 0.0945351 |
| Korean Saanen | H239 | 260122 | 0.104649 |
| Longlin goat | L1 | 194711 | 0.0783338 |
| Longlin goat | L2 | 244481 | 0.0983566 |
| Longlin goat | L3 | 259776 | 0.10451 |
| Longlin goat | L4 | 266809 | 0.107339 |
| Longlin goat | L5 | 243418 | 0.097929 |
| Longlin goat | L606 | 328070 | 0.131985 |
| Longlin goat | L607 | 195287 | 0.0785655 |
| Longlin goat | L608 | 160091 | 0.0644059 |
| Longlin goat | L609 | 165004 | 0.0663824 |
| Longlin goat | L610 | 161872 | 0.0651224 |
| Longlin goat | L720 | 256832 | 0.103326 |
| Longlin goat | L721 | 187674 | 0.0755027 |
| Longlin goat | L722 | 93905.5 | 0.0377789 |
| Longlin goat | L723 | 198220 | 0.0797455 |
| Longlin goat | L724 | 181605 | 0.0730611 |
| New Zealand Alpine | 427 | 208138 | 0.0837356 |
| New Zealand Alpine | 520 | 270833 | 0.108958 |
| New Zealand Alpine | 622 | 286577 | 0.115292 |
| New Zealand Alpine | 645 | 229743 | 0.0924274 |
| Chinese Nubian * | N10 | 51601.7 | 0.0207598 |
| Chinese Nubian * | N11 | 93658.6 | 0.0376796 |
| Chinese Nubian * | N12 | 156476 | 0.0629515 |
| Chinese Nubian * | N13 | 112557 | 0.0452826 |
| New Zealand Saanen | 1639 | 226792 | 0.0912402 |
| New Zealand Saanen | 1872 | 221096 | 0.0889487 |
| New Zealand Saanen | 1943 | 284685 | 0.114531 |
| New Zealand Saanen | 205 | 275819 | 0.110964 |
| New Zealand Saanen | 456 | 226520 | 0.0911308 |
| New Zealand Saanen | 516 | 161461 | 0.064957 |
| Toggenburg | 19 | 394414 | 0.158676 |
| Toggenburg | 20 | 304978 | 0.122695 |
| Toggenburg | 21 | 409371 | 0.164693 |
| Toggenburg | 22 | 384831 | 0.154821 |
| Toggenburg | 23 | 579179 | 0.233008 |
| Toggenburg | 24 | 440081 | 0.177048 |
| Yunshang black goat | 10 | 330976 | 0.133154 |
| Yunshang black goat | 11 | 252478 | 0.101574 |
| Yunshang black goat | 12 | 311141 | 0.125174 |
| Yunshang black goat | 7 | 364303 | 0.146562 |
| Yunshang black goat | 8 | 171096 | 0.0688333 |
| Yunshang black goat | 9 | 256423 | 0.103161 |

**Table S5.** Candidate regions and genes under positive selection in dairy goat.

| **Chr** | **BIN_START** | **BIN_END** | **Chr** | |  | **Symbol** | | **Start** | **End** | **Gene ID** |
| --- | --- | --- | --- | --- | --- | --- | --- | --- | --- | --- |
| 10 | 11750001 | 11800000 | 10 | Liftoff | | gene | 11743536 | | 11755398 | PTGER2 |
| 10 | 11750001 | 11800000 | 10 | Liftoff | | gene | 11765156 | | 11808522 | ANKDD1A |
| 10 | 11825001 | 11875000 | 10 | Liftoff | | gene | 11819778 | | 11845566 | SPG21 |
| 10 | 11825001 | 11875000 | 10 | Liftoff | | gene | 11855841 | | 11880020 | MTFMT |
| 10 | 21200001 | 21250000 | 10 | Liftoff | | gene | 21216068 | | 21237846 | LOC442995 |
| 10 | 21200001 | 21250000 | 10 | Liftoff | | gene | 21242979 | | 21269425 | LOC101120580 |
| 10 | 36375001 | 36425000 | 10 | Liftoff | | gene | 36369796 | | 36386426 | RPAP1 |
| 10 | 36375001 | 36425000 | 10 | Liftoff | | gene | 36392567 | | 36411340 | TYRO3 |
| 10 | 36400001 | 36450000 | 10 | Liftoff | | gene | 36392567 | | 36411340 | TYRO3 |
| 10 | 4075001 | 4125000 | 10 | Liftoff | | gene | 4031481 | | 4090044 | PGGT1B |
| 10 | 4075001 | 4125000 | 10 | Liftoff | | gene | 4094225 | | 4102367 | LOC105612984 |
| 10 | 4075001 | 4125000 | 10 | Liftoff | | gene | 4094296 | | 4125372 | CCDC112 |
| 10 | 4100001 | 4150000 | 10 | Liftoff | | gene | 4094225 | | 4102367 | LOC105612984 |
| 10 | 4100001 | 4150000 | 10 | Liftoff | | gene | 4094296 | | 4125372 | CCDC112 |
| 10 | 42225001 | 42275000 | 10 | Liftoff | | gene | 42223609 | | 42277875 | NEMF |
| 10 | 42250001 | 42300000 | 10 | Liftoff | | gene | 42223609 | | 42277875 | NEMF |
| 10 | 42250001 | 42300000 | 10 | Liftoff | | gene | 42292651 | | 42292762 | LOC114115902 |
| 10 | 43925001 | 43975000 | 10 | Liftoff | | gene | 43837563 | | 44013255 | FRMD6 |
| 10 | 43925001 | 43975000 | 10 | Liftoff | | gene | 43928698 | | 43929459 | LOC114115851 |
| 10 | 43950001 | 44000000 | 10 | Liftoff | | gene | 43837563 | | 44013255 | FRMD6 |
| 10 | 46225001 | 46275000 | 10 | Liftoff | | gene | 46165211 | | 46233831 | RAB8B |
| 10 | 46225001 | 46275000 | 10 | Liftoff | | gene | 46272595 | | 46276044 | RPS27L |
| 10 | 47300001 | 47350000 | 10 | Liftoff | | gene | 47327806 | | 47327941 | LOC114115938 |
| 10 | 47325001 | 47375000 | 10 | Liftoff | | gene | 47327806 | | 47327941 | LOC114115938 |
| 10 | 47350001 | 47400000 | 10 | Liftoff | | gene | 47398870 | | 47401198 | LOC101102735 |
| 10 | 47375001 | 47425000 | 10 | Liftoff | | gene | 47398870 | | 47401198 | LOC101102735 |
| 10 | 47400001 | 47450000 | 10 | Liftoff | | gene | 47398870 | | 47401198 | LOC101102735 |
| 10 | 47400001 | 47450000 | 10 | Liftoff | | gene | 47414720 | | 47596080 | VPS13C |
| 10 | 64750001 | 64800000 | 10 | Liftoff | | gene | 64766834 | | 64773389 | LOC101115542 |
| 10 | 68200001 | 68250000 | 10 | Liftoff | | gene | 68225687 | | 68296501 | TMEM260 |
| 10 | 68225001 | 68275000 | 10 | Liftoff | | gene | 68225687 | | 68296501 | TMEM260 |
| 10 | 72050001 | 72100000 | 10 | Liftoff | | gene | 72067189 | | 72070026 | SIX1 |
| 10 | 72050001 | 72100000 | 10 | Liftoff | | gene | 72077131 | | 72079768 | LOC106991287 |
| 10 | 75550001 | 75600000 | 10 | Liftoff | | gene | 75333125 | | 75657542 | SYNE2 |
| 10 | 75550001 | 75600000 | 10 | Liftoff | | gene | 75542164 | | 75556199 | LOC105607030 |
| 10 | 75575001 | 75625000 | 10 | Liftoff | | gene | 75333125 | | 75657542 | SYNE2 |
| 10 | 75600001 | 75650000 | 10 | Liftoff | | gene | 75333125 | | 75657542 | SYNE2 |
| 10 | 75625001 | 75675000 | 10 | Liftoff | | gene | 75333125 | | 75657542 | SYNE2 |
| 10 | 75625001 | 75675000 | 10 | Liftoff | | gene | 75665804 | | 75711025 | ESR2 |
| 10 | 76350001 | 76400000 | 10 | Liftoff | | gene | 76328852 | | 76353109 | RAB15 |
| 10 | 76350001 | 76400000 | 10 | Liftoff | | gene | 76363846 | | 76442005 | FNTB |
| 10 | 8150001 | 8200000 | 10 | Liftoff | | gene | 8160093 | | 8204512 | AGGF1 |
| 10 | 8175001 | 8225000 | 10 | Liftoff | | gene | 8160093 | | 8204512 | AGGF1 |
| 10 | 8175001 | 8225000 | 10 | Liftoff | | gene | 8214217 | | 8233063 | ZBED3 |
| 10 | 84625001 | 84675000 | 10 | Liftoff | | gene | 84613714 | | 84645230 | ENTPD5 |
| 10 | 84625001 | 84675000 | 10 | Liftoff | | gene | 84645340 | | 84679049 | BBOF1 |
| 10 | 84650001 | 84700000 | 10 | Liftoff | | gene | 84645340 | | 84679049 | BBOF1 |
| 10 | 84650001 | 84700000 | 10 | Liftoff | | gene | 84674861 | | 84695262 | ALDH6A1 |
| 10 | 85200001 | 85250000 | 10 | Liftoff | | gene | 85198258 | | 85243583 | AREL1 |
| 10 | 85200001 | 85250000 | 10 | Liftoff | | gene | 85243618 | | 85253852 | FCF1 |
| 10 | 85225001 | 85275000 | 10 | Liftoff | | gene | 85198258 | | 85243583 | AREL1 |
| 10 | 85225001 | 85275000 | 10 | Liftoff | | gene | 85243618 | | 85253852 | FCF1 |
| 10 | 85250001 | 85300000 | 10 | Liftoff | | gene | 85243618 | | 85253852 | FCF1 |
| 10 | 85250001 | 85300000 | 10 | Liftoff | | gene | 85274017 | | 85336530 | YLPM1 |
| 10 | 85275001 | 85325000 | 10 | Liftoff | | gene | 85274017 | | 85336530 | YLPM1 |
| 10 | 85300001 | 85350000 | 10 | Liftoff | | gene | 85274017 | | 85336530 | YLPM1 |
| 10 | 85325001 | 85375000 | 10 | Liftoff | | gene | 85274017 | | 85336530 | YLPM1 |
| 10 | 85325001 | 85375000 | 10 | Liftoff | | gene | 85361578 | | 85370461 | PROX2 |
| 10 | 91700001 | 91750000 | 10 | Liftoff | | gene | 91558965 | | 91736512 | TSHR |
| 10 | 91750001 | 91800000 | 10 | Liftoff | | gene | 91781228 | | 91820898 | GTF2A1 |
| 10 | 91775001 | 91825000 | 10 | Liftoff | | gene | 91781228 | | 91820898 | GTF2A1 |
| 10 | 91775001 | 91825000 | 10 | Liftoff | | gene | 91807609 | | 91807752 | LOC114115944 |
| 10 | 91775001 | 91825000 | 10 | Liftoff | | gene | 91816763 | | 91864536 | LOC114115657 |
| 10 | 91800001 | 91850000 | 10 | Liftoff | | gene | 91781228 | | 91820898 | GTF2A1 |
| 10 | 91800001 | 91850000 | 10 | Liftoff | | gene | 91807609 | | 91807752 | LOC114115944 |
| 10 | 91800001 | 91850000 | 10 | Liftoff | | gene | 91816763 | | 91864536 | LOC114115657 |
| 1 | 106225001 | 106275000 | 1 | Liftoff | | gene | 106247292 | | 106308573 | KPNA4 |
| 1 | 106250001 | 106300000 | 1 | Liftoff | | gene | 106247292 | | 106308573 | KPNA4 |
| 1 | 106250001 | 106300000 | 1 | Liftoff | | gene | 106292481 | | 106292813 | LOC114111379 |
| 1 | 106275001 | 106325000 | 1 | Liftoff | | gene | 106247292 | | 106308573 | KPNA4 |
| 1 | 106275001 | 106325000 | 1 | Liftoff | | gene | 106292481 | | 106292813 | LOC114111379 |
| 11 | 102550001 | 102600000 | 11 | Liftoff | | gene | 102440166 | | 102595472 | CFAP77 |
| 11 | 103675001 | 103725000 | 11 | Liftoff | | gene | 103684038 | | 103692295 | LOC114113858 |
| 11 | 103675001 | 103725000 | 11 | Liftoff | | gene | 103692334 | | 103728213 | CCDC187 |
| 11 | 103700001 | 103750000 | 11 | Liftoff | | gene | 103692334 | | 103728213 | CCDC187 |
| 11 | 11125001 | 11175000 | 11 | Liftoff | | gene | 11040093 | | 11153010 | TET3 |
| 11 | 11150001 | 11200000 | 11 | Liftoff | | gene | 11173858 | | 11207478 | DGUOK |
| 1 | 111200001 | 111250000 | 1 | Liftoff | | gene | 111226990 | | 111251279 | C1H3orf33 |
| 11 | 11200001 | 11250000 | 11 | Liftoff | | gene | 11173858 | | 11207478 | DGUOK |
| 11 | 11200001 | 11250000 | 11 | Liftoff | | gene | 11213547 | | 11237730 | ACTG2 |
| 11 | 11225001 | 11275000 | 11 | Liftoff | | gene | 11213547 | | 11237730 | ACTG2 |
| 11 | 11225001 | 11275000 | 11 | Liftoff | | gene | 11271308 | | 11294787 | STAMBP |
| 11 | 1725001 | 1775000 | 11 | Liftoff | | gene | 1671349 | | 1740307 | BCL2L11 |
| 11 | 17800001 | 17850000 | 11 | Liftoff | | gene | 17841360 | | 17841421 | LOC114114214 |
| 11 | 21125001 | 21175000 | 11 | Liftoff | | gene | 21126816 | | 21194733 | ATL2 |
| 11 | 25725001 | 25775000 | 11 | Liftoff | | gene | 25769361 | | 25788744 | LOC114113941 |
| 11 | 29250001 | 29300000 | 11 | Liftoff | | gene | 29228377 | | 29258613 | PIGF |
| 11 | 29250001 | 29300000 | 11 | Liftoff | | gene | 29258776 | | 29269781 | CRIPT |
| 1 | 131700001 | 131750000 | 1 | Liftoff | | gene | 131699576 | | 132170341 | STAG1 |
| 1 | 138575001 | 138625000 | 1 | Liftoff | | gene | 138544423 | | 138668161 | BRWD1 |
| 1 | 138600001 | 138650000 | 1 | Liftoff | | gene | 138544423 | | 138668161 | BRWD1 |
| 1 | 138600001 | 138650000 | 1 | Liftoff | | gene | 138643626 | | 138643732 | LOC114110980 |
| 11 | 3975001 | 4025000 | 11 | Liftoff | | gene | 3929445 | | 4115768 | VWA3B |
| 11 | 4000001 | 4050000 | 11 | Liftoff | | gene | 3929445 | | 4115768 | VWA3B |
| 11 | 4025001 | 4075000 | 11 | Liftoff | | gene | 3929445 | | 4115768 | VWA3B |
| 11 | 4050001 | 4100000 | 11 | Liftoff | | gene | 3929445 | | 4115768 | VWA3B |
| 11 | 4075001 | 4125000 | 11 | Liftoff | | gene | 3929445 | | 4115768 | VWA3B |
| 11 | 4100001 | 4150000 | 11 | Liftoff | | gene | 4139749 | | 4178151 | CNGA3 |
| 11 | 4125001 | 4175000 | 11 | Liftoff | | gene | 4139749 | | 4178151 | CNGA3 |
| 11 | 4400001 | 4450000 | 11 | Liftoff | | gene | 4387560 | | 4509156 | MGAT4A |
| 11 | 4400001 | 4450000 | 11 | Liftoff | | gene | 4429576 | | 4431142 | LOC105612510 |
| 11 | 4425001 | 4475000 | 11 | Liftoff | | gene | 4387560 | | 4509156 | MGAT4A |
| 11 | 4425001 | 4475000 | 11 | Liftoff | | gene | 4429576 | | 4431142 | LOC105612510 |
| 11 | 5075001 | 5125000 | 11 | Liftoff | | gene | 5064399 | | 5143958 | REV1 |
| 1 | 151800001 | 151850000 | 1 | Liftoff | | gene | 151747622 | | 151818454 | SH3BP5 |
| 1 | 151800001 | 151850000 | 1 | Liftoff | | gene | 151842252 | | 151844909 | LOC114110645 |
| 11 | 57125001 | 57175000 | 11 | Liftoff | | gene | 57169886 | | 57169994 | LOC114114188 |
| 11 | 6725001 | 6775000 | 11 | Liftoff | | gene | 6710586 | | 6741562 | RNF149 |
| 11 | 68800001 | 68850000 | 11 | Liftoff | | gene | 68766371 | | 68805454 | C3H2orf42 |
| 11 | 68800001 | 68850000 | 11 | Liftoff | | gene | 68790846 | | 68843663 | TIA1 |
| 11 | 68800001 | 68850000 | 11 | Liftoff | | gene | 68843954 | | 68844024 | TRNAG-CCC-27 |
| 11 | 70950001 | 71000000 | 11 | Liftoff | | gene | 70983998 | | 71049627 | CLIP4 |
| 11 | 71650001 | 71700000 | 11 | Liftoff | | gene | 71638890 | | 71662034 | FOSL2 |
| 11 | 71800001 | 71850000 | 11 | Liftoff | | gene | 71714412 | | 72117871 | BABAM2 |
| 11 | 71825001 | 71875000 | 11 | Liftoff | | gene | 71714412 | | 72117871 | BABAM2 |
| 11 | 71850001 | 71900000 | 11 | Liftoff | | gene | 71714412 | | 72117871 | BABAM2 |
| 11 | 94000001 | 94050000 | 11 | Liftoff | | gene | 93995059 | | 94001057 | LOC101114946 |
| 11 | 94000001 | 94050000 | 11 | Liftoff | | gene | 94023006 | | 94023965 | LOC101114434 |
| 11 | 94000001 | 94050000 | 11 | Liftoff | | gene | 94030846 | | 94035919 | LOC101114183 |
| 11 | 94000001 | 94050000 | 11 | Liftoff | | gene | 94043213 | | 94052777 | PDCL |
| 11 | 94025001 | 94075000 | 11 | Liftoff | | gene | 94030846 | | 94035919 | LOC101114183 |
| 11 | 94025001 | 94075000 | 11 | Liftoff | | gene | 94043213 | | 94052777 | PDCL |
| 11 | 94025001 | 94075000 | 11 | Liftoff | | gene | 94052713 | | 94069865 | LOC114113884 |
| 11 | 94025001 | 94075000 | 11 | Liftoff | | gene | 94066983 | | 94119338 | RC3H2 |
| 11 | 950001 | 1000000 | 11 | Liftoff | | gene | 888827 | | 1008462 | MERTK |
| 11 | 950001 | 1000000 | 11 | Liftoff | | gene | 996647 | | 996781 | LOC114114227 |
| 11 | 975001 | 1025000 | 11 | Liftoff | | gene | 888827 | | 1008462 | MERTK |
| 11 | 975001 | 1025000 | 11 | Liftoff | | gene | 996647 | | 996781 | LOC114114227 |
| 11 | 99275001 | 99325000 | 11 | Liftoff | | gene | 99271732 | | 99301282 | ZER1 |
| 11 | 99275001 | 99325000 | 11 | Liftoff | | gene | 99306021 | | 99311854 | LOC114113872 |
| 11 | 99275001 | 99325000 | 11 | Liftoff | | gene | 99312047 | | 99327757 | TBC1D13 |
| 12 | 17200001 | 17250000 | 12 | Liftoff | | gene | 17204378 | | 17425157 | LRCH1 |
| 12 | 19100001 | 19150000 | 12 | Liftoff | | gene | 19126044 | | 19146447 | CYSLTR2 |
| 12 | 28850001 | 28900000 | 12 | Liftoff | | gene | 28873369 | | 29047474 | PDS5B |
| 12 | 28875001 | 28925000 | 12 | Liftoff | | gene | 28873369 | | 29047474 | PDS5B |
| 12 | 28900001 | 28950000 | 12 | Liftoff | | gene | 28873369 | | 29047474 | PDS5B |
| 12 | 28925001 | 28975000 | 12 | Liftoff | | gene | 28873369 | | 29047474 | PDS5B |
| 12 | 28950001 | 29000000 | 12 | Liftoff | | gene | 28873369 | | 29047474 | PDS5B |
| 12 | 28975001 | 29025000 | 12 | Liftoff | | gene | 28873369 | | 29047474 | PDS5B |
| 12 | 29000001 | 29050000 | 12 | Liftoff | | gene | 28873369 | | 29047474 | PDS5B |
| 12 | 36725001 | 36775000 | 12 | Liftoff | | gene | 36741218 | | 36766396 | ZMYM5 |
| 12 | 36750001 | 36800000 | 12 | Liftoff | | gene | 36741218 | | 36766396 | ZMYM5 |
| 12 | 36750001 | 36800000 | 12 | Liftoff | | gene | 36776292 | | 36851396 | PSPC1 |
| 12 | 36775001 | 36825000 | 12 | Liftoff | | gene | 36776292 | | 36851396 | PSPC1 |
| 1 | 2600001 | 2650000 | 1 | Liftoff | | gene | 2499084 | | 2622366 | EVA1C |
| 12 | 76750001 | 76800000 | 12 | Liftoff | | gene | 76755641 | | 77044112 | DOCK9 |
| 12 | 77250001 | 77300000 | 12 | Liftoff | | gene | 77119043 | | 77290370 | UBAC2 |
| 12 | 77500001 | 77550000 | 12 | Liftoff | | gene | 77465533 | | 77704216 | CLYBL |
| 12 | 77525001 | 77575000 | 12 | Liftoff | | gene | 77465533 | | 77704216 | CLYBL |
| 12 | 77550001 | 77600000 | 12 | Liftoff | | gene | 77465533 | | 77704216 | CLYBL |
| 12 | 77575001 | 77625000 | 12 | Liftoff | | gene | 77465533 | | 77704216 | CLYBL |
| 12 | 77600001 | 77650000 | 12 | Liftoff | | gene | 77465533 | | 77704216 | CLYBL |
| 12 | 79950001 | 80000000 | 12 | Liftoff | | gene | 79908762 | | 79967432 | TPP2 |
| 12 | 79950001 | 80000000 | 12 | Liftoff | | gene | 79971864 | | 79982980 | METTL21C |
| 13 | 15750001 | 15800000 | 13 | Liftoff | | gene | 15750959 | | 15755516 | LOC114117552 |
| 13 | 15750001 | 15800000 | 13 | Liftoff | | gene | 15755707 | | 15799541 | PROSER2 |
| 13 | 15850001 | 15900000 | 13 | Liftoff | | gene | 15839849 | | 15934230 | UPF2 |
| 13 | 15875001 | 15925000 | 13 | Liftoff | | gene | 15839849 | | 15934230 | UPF2 |
| 13 | 15900001 | 15950000 | 13 | Liftoff | | gene | 15839849 | | 15934230 | UPF2 |
| 13 | 15925001 | 15975000 | 13 | Liftoff | | gene | 15953835 | | 15991294 | DHTKD1 |
| 13 | 16200001 | 16250000 | 13 | Liftoff | | gene | 16167215 | | 16562464 | CAMK1D |
| 13 | 21975001 | 22025000 | 13 | Liftoff | | gene | 21984299 | | 21999196 | SKIDA1 |
| 13 | 22000001 | 22050000 | 13 | Liftoff | | gene | 22007426 | | 22220479 | MLLT10 |
| 13 | 22025001 | 22075000 | 13 | Liftoff | | gene | 22007426 | | 22220479 | MLLT10 |
| 13 | 22050001 | 22100000 | 13 | Liftoff | | gene | 22007426 | | 22220479 | MLLT10 |
| 13 | 22075001 | 22125000 | 13 | Liftoff | | gene | 22007426 | | 22220479 | MLLT10 |
| 13 | 22100001 | 22150000 | 13 | Liftoff | | gene | 22007426 | | 22220479 | MLLT10 |
| 13 | 22125001 | 22175000 | 13 | Liftoff | | gene | 22007426 | | 22220479 | MLLT10 |
| 13 | 22150001 | 22200000 | 13 | Liftoff | | gene | 22007426 | | 22220479 | MLLT10 |
| 13 | 22175001 | 22225000 | 13 | Liftoff | | gene | 22007426 | | 22220479 | MLLT10 |
| 13 | 22975001 | 23025000 | 13 | Liftoff | | gene | 22891579 | | 23073518 | PIP4K2A |
| 13 | 23000001 | 23050000 | 13 | Liftoff | | gene | 22891579 | | 23073518 | PIP4K2A |
| 13 | 27550001 | 27600000 | 13 | Liftoff | | gene | 27526980 | | 27851497 | FRMD4A |
| 13 | 28725001 | 28775000 | 13 | Liftoff | | gene | 28726190 | | 28764793 | DCLRE1C |
| 13 | 28725001 | 28775000 | 13 | Liftoff | | gene | 28772571 | | 28788623 | MEIG1 |
| 13 | 31225001 | 31275000 | 13 | Liftoff | | gene | 31265740 | | 31285715 | HACD1 |
| 13 | 31225001 | 31275000 | 13 | Liftoff | | gene | 31274870 | | 31275479 | LOC114117564 |
| 13 | 31250001 | 31300000 | 13 | Liftoff | | gene | 31265740 | | 31285715 | HACD1 |
| 13 | 31250001 | 31300000 | 13 | Liftoff | | gene | 31274870 | | 31275479 | LOC114117564 |
| 13 | 31350001 | 31400000 | 13 | Liftoff | | gene | 31310393 | | 31363811 | STAM |
| 13 | 31350001 | 31400000 | 13 | Liftoff | | gene | 31366152 | | 31372362 | LOC114117565 |
| 13 | 31350001 | 31400000 | 13 | Liftoff | | gene | 31388588 | | 31391184 | LOC114117497 |
| 13 | 31350001 | 31400000 | 13 | Liftoff | | gene | 31391434 | | 31430463 | TMEM236 |
| 13 | 31375001 | 31425000 | 13 | Liftoff | | gene | 31388588 | | 31391184 | LOC114117497 |
| 13 | 31375001 | 31425000 | 13 | Liftoff | | gene | 31391434 | | 31430463 | TMEM236 |
| 13 | 31400001 | 31450000 | 13 | Liftoff | | gene | 31391434 | | 31430463 | TMEM236 |
| 13 | 31425001 | 31475000 | 13 | Liftoff | | gene | 31391434 | | 31430463 | TMEM236 |
| 13 | 31425001 | 31475000 | 13 | Liftoff | | gene | 31447510 | | 31558566 | MRC1 |
| 13 | 32350001 | 32400000 | 13 | Liftoff | | gene | 32323448 | | 32420221 | EPC1 |
| 13 | 35200001 | 35250000 | 13 | Liftoff | | gene | 35247913 | | 35252905 | BAMBI |
| 13 | 35225001 | 35275000 | 13 | Liftoff | | gene | 35247913 | | 35252905 | BAMBI |
| 1 | 33600001 | 33650000 | 1 | Liftoff | | gene | 33578037 | | 33739662 | LOC114110024 |
| 13 | 42075001 | 42125000 | 13 | Liftoff | | gene | 42082170 | | 42117177 | PYGB |
| 13 | 42075001 | 42125000 | 13 | Liftoff | | gene | 42119252 | | 42172115 | ABHD12 |
| 13 | 42100001 | 42150000 | 13 | Liftoff | | gene | 42082170 | | 42117177 | PYGB |
| 13 | 42100001 | 42150000 | 13 | Liftoff | | gene | 42119252 | | 42172115 | ABHD12 |
| 13 | 42875001 | 42925000 | 13 | Liftoff | | gene | 42878630 | | 42898479 | LOC106990122 |
| 13 | 42875001 | 42925000 | 13 | Liftoff | | gene | 42923504 | | 42933300 | LOC101109111 |
| 13 | 44425001 | 44475000 | 13 | Liftoff | | gene | 44454416 | | 44480301 | PITRM1 |
| 13 | 45800001 | 45850000 | 13 | Liftoff | | gene | 45788073 | | 45845126 | LARP4B |
| 13 | 46200001 | 46250000 | 13 | Liftoff | | gene | 46208455 | | 46282630 | ZMYND11 |
| 13 | 46225001 | 46275000 | 13 | Liftoff | | gene | 46208455 | | 46282630 | ZMYND11 |
| 13 | 50400001 | 50450000 | 13 | Liftoff | | gene | 50413736 | | 50437778 | ADRA1D |
| 13 | 50425001 | 50475000 | 13 | Liftoff | | gene | 50413736 | | 50437778 | ADRA1D |
| 13 | 50425001 | 50475000 | 13 | Liftoff | | gene | 50452345 | | 50489300 | SMOX |
| 13 | 52125001 | 52175000 | 13 | Liftoff | | gene | 52038761 | | 52138230 | TMC2 |
| 13 | 52125001 | 52175000 | 13 | Liftoff | | gene | 52163497 | | 52172126 | SNRPB |
| 13 | 52650001 | 52700000 | 13 | Liftoff | | gene | 52674400 | | 52678054 | LOC105616720 |
| 13 | 52650001 | 52700000 | 13 | Liftoff | | gene | 52684587 | | 52729301 | SIRPA |
| 13 | 53200001 | 53250000 | 13 | Liftoff | | gene | 53152908 | | 53216076 | MYT1 |
| 13 | 53200001 | 53250000 | 13 | Liftoff | | gene | 53246367 | | 53247377 | NPBWR2 |
| 13 | 53225001 | 53275000 | 13 | Liftoff | | gene | 53246367 | | 53247377 | NPBWR2 |
| 13 | 53225001 | 53275000 | 13 | Liftoff | | gene | 53250973 | | 53269901 | OPRL1 |
| 13 | 53225001 | 53275000 | 13 | Liftoff | | gene | 53265761 | | 53267928 | LKAAEAR1 |
| 13 | 53225001 | 53275000 | 13 | Liftoff | | gene | 53270069 | | 53276184 | RGS19 |
| 13 | 53250001 | 53300000 | 13 | Liftoff | | gene | 53250973 | | 53269901 | OPRL1 |
| 13 | 53250001 | 53300000 | 13 | Liftoff | | gene | 53265761 | | 53267928 | LKAAEAR1 |
| 13 | 53250001 | 53300000 | 13 | Liftoff | | gene | 53270069 | | 53276184 | RGS19 |
| 13 | 53250001 | 53300000 | 13 | Liftoff | | gene | 53277014 | | 53296249 | TCEA2 |
| 13 | 53250001 | 53300000 | 13 | Liftoff | | gene | 53295308 | | 53297176 | SOX18 |
| 13 | 53275001 | 53325000 | 13 | Liftoff | | gene | 53270069 | | 53276184 | RGS19 |
| 13 | 53275001 | 53325000 | 13 | Liftoff | | gene | 53277014 | | 53296249 | TCEA2 |
| 13 | 53275001 | 53325000 | 13 | Liftoff | | gene | 53295308 | | 53297176 | SOX18 |
| 13 | 53275001 | 53325000 | 13 | Liftoff | | gene | 53304130 | | 53305379 | C13H20orf204 |
| 13 | 53275001 | 53325000 | 13 | Liftoff | | gene | 53308630 | | 53344305 | PRPF6 |
| 13 | 53300001 | 53350000 | 13 | Liftoff | | gene | 53304130 | | 53305379 | C13H20orf204 |
| 13 | 53300001 | 53350000 | 13 | Liftoff | | gene | 53308630 | | 53344305 | PRPF6 |
| 13 | 53300001 | 53350000 | 13 | Liftoff | | gene | 53338987 | | 53339093 | LOC114117706 |
| 13 | 53300001 | 53350000 | 13 | Liftoff | | gene | 53345848 | | 53350350 | SAMD10 |
| 13 | 53325001 | 53375000 | 13 | Liftoff | | gene | 53308630 | | 53344305 | PRPF6 |
| 13 | 53325001 | 53375000 | 13 | Liftoff | | gene | 53338987 | | 53339093 | LOC114117706 |
| 13 | 53325001 | 53375000 | 13 | Liftoff | | gene | 53345848 | | 53350350 | SAMD10 |
| 13 | 53325001 | 53375000 | 13 | Liftoff | | gene | 53352577 | | 53363163 | ZNF512B |
| 13 | 53325001 | 53375000 | 13 | Liftoff | | gene | 53362981 | | 53368981 | LOC114117598 |
| 13 | 53325001 | 53375000 | 13 | Liftoff | | gene | 53363402 | | 53374650 | UCKL1 |
| 13 | 53350001 | 53400000 | 13 | Liftoff | | gene | 53352577 | | 53363163 | ZNF512B |
| 13 | 53350001 | 53400000 | 13 | Liftoff | | gene | 53362981 | | 53368981 | LOC114117598 |
| 13 | 53350001 | 53400000 | 13 | Liftoff | | gene | 53363402 | | 53374650 | UCKL1 |
| 13 | 53350001 | 53400000 | 13 | Liftoff | | gene | 53375619 | | 53381070 | LOC114117599 |
| 13 | 53350001 | 53400000 | 13 | Liftoff | | gene | 53380177 | | 53409463 | DNAJC5 |
| 13 | 53375001 | 53425000 | 13 | Liftoff | | gene | 53375619 | | 53381070 | LOC114117599 |
| 13 | 53375001 | 53425000 | 13 | Liftoff | | gene | 53380177 | | 53409463 | DNAJC5 |
| 13 | 53375001 | 53425000 | 13 | Liftoff | | gene | 53413396 | | 53429559 | TPD52L2 |
| 13 | 53400001 | 53450000 | 13 | Liftoff | | gene | 53380177 | | 53409463 | DNAJC5 |
| 13 | 53400001 | 53450000 | 13 | Liftoff | | gene | 53413396 | | 53429559 | TPD52L2 |
| 13 | 53400001 | 53450000 | 13 | Liftoff | | gene | 53429613 | | 53432769 | ABHD16B |
| 13 | 53425001 | 53475000 | 13 | Liftoff | | gene | 53413396 | | 53429559 | TPD52L2 |
| 13 | 53425001 | 53475000 | 13 | Liftoff | | gene | 53429613 | | 53432769 | ABHD16B |
| 13 | 53425001 | 53475000 | 13 | Liftoff | | gene | 53453364 | | 53503904 | ZBTB46 |
| 13 | 56500001 | 56550000 | 13 | Liftoff | | gene | 56534911 | | 56559520 | EDN3 |
| 13 | 56525001 | 56575000 | 13 | Liftoff | | gene | 56534911 | | 56559520 | EDN3 |
| 13 | 56550001 | 56600000 | 13 | Liftoff | | gene | 56534911 | | 56559520 | EDN3 |
| 13 | 56625001 | 56675000 | 13 | Liftoff | | gene | 56657757 | | 56674797 | ZNF831 |
| 13 | 56650001 | 56700000 | 13 | Liftoff | | gene | 56657757 | | 56674797 | ZNF831 |
| 13 | 56950001 | 57000000 | 13 | Liftoff | | gene | 56927758 | | 56994038 | LOC101115640 |
| 13 | 56950001 | 57000000 | 13 | Liftoff | | gene | 56929178 | | 56994476 | LOC101102411 |
| 13 | 56950001 | 57000000 | 13 | Liftoff | | gene | 56981487 | | 57013226 | LOC105606221 |
| 13 | 60175001 | 60225000 | 13 | Liftoff | | gene | 60174647 | | 60197639 | C13H20orf96 |
| 13 | 60175001 | 60225000 | 13 | Liftoff | | gene | 60208121 | | 60209859 | DEFB132 |
| 13 | 60175001 | 60225000 | 13 | Liftoff | | gene | 60224378 | | 60226641 | DEFB129 |
| 13 | 62050001 | 62100000 | 13 | Liftoff | | gene | 62066193 | | 62076626 | LOC101115983 |
| 13 | 62050001 | 62100000 | 13 | Liftoff | | gene | 62077301 | | 62093992 | LOC114117617 |
| 13 | 62050001 | 62100000 | 13 | Liftoff | | gene | 62097876 | | 62100303 | LOC114117615 |
| 13 | 62075001 | 62125000 | 13 | Liftoff | | gene | 62066193 | | 62076626 | LOC101115983 |
| 13 | 62075001 | 62125000 | 13 | Liftoff | | gene | 62077301 | | 62093992 | LOC114117617 |
| 13 | 62075001 | 62125000 | 13 | Liftoff | | gene | 62097876 | | 62100303 | LOC114117615 |
| 13 | 62100001 | 62150000 | 13 | Liftoff | | gene | 62097876 | | 62100303 | LOC114117615 |
| 13 | 62125001 | 62175000 | 13 | Liftoff | | gene | 62162093 | | 62172303 | LOC114117619 |
| 13 | 62150001 | 62200000 | 13 | Liftoff | | gene | 62162093 | | 62172303 | LOC114117619 |
| 13 | 62150001 | 62200000 | 13 | Liftoff | | gene | 62197016 | | 62203650 | LOC114117485 |
| 13 | 62400001 | 62450000 | 13 | Liftoff | | gene | 62405048 | | 62421052 | LOC101116500 |
| 13 | 62400001 | 62450000 | 13 | Liftoff | | gene | 62438337 | | 62448660 | BPIFA3 |
| 13 | 62425001 | 62475000 | 13 | Liftoff | | gene | 62438337 | | 62448660 | BPIFA3 |
| 13 | 62425001 | 62475000 | 13 | Liftoff | | gene | 62456481 | | 62463497 | BPIFA1 |
| 13 | 63900001 | 63950000 | 13 | Liftoff | | gene | 63881309 | | 63905806 | GGT7 |
| 13 | 63900001 | 63950000 | 13 | Liftoff | | gene | 63909357 | | 63954092 | ACSS2 |
| 13 | 64975001 | 65025000 | 13 | Liftoff | | gene | 64958665 | | 65076077 | EPB41L1 |
| 13 | 65300001 | 65350000 | 13 | Liftoff | | gene | 65208974 | | 65349844 | DLGAP4 |
| 13 | 65325001 | 65375000 | 13 | Liftoff | | gene | 65208974 | | 65349844 | DLGAP4 |
| 13 | 65325001 | 65375000 | 13 | Liftoff | | gene | 65361407 | | 65369736 | MYL9 |
| 13 | 65350001 | 65400000 | 13 | Liftoff | | gene | 65361407 | | 65369736 | MYL9 |
| 13 | 65350001 | 65400000 | 13 | Liftoff | | gene | 65390508 | | 65411850 | TGIF2 |
| 13 | 7125001 | 7175000 | 13 | Liftoff | | gene | 7130134 | | 7189000 | SEL1L2 |
| 13 | 72325001 | 72375000 | 13 | Liftoff | | gene | 72269581 | | 72342645 | JPH2 |
| 13 | 72325001 | 72375000 | 13 | Liftoff | | gene | 72351731 | | 72361538 | OSER1 |
| 13 | 72325001 | 72375000 | 13 | Liftoff | | gene | 72361661 | | 72363795 | LOC105610214 |
| 13 | 72325001 | 72375000 | 13 | Liftoff | | gene | 72364158 | | 72364947 | LOC114117632 |
| 13 | 72975001 | 73025000 | 13 | Liftoff | | gene | 72967119 | | 72988985 | YWHAB |
| 13 | 72975001 | 73025000 | 13 | Liftoff | | gene | 72991102 | | 73015231 | PABPC1L |
| 13 | 72975001 | 73025000 | 13 | Liftoff | | gene | 73016495 | | 73037370 | TOMM34 |
| 13 | 73200001 | 73250000 | 13 | Liftoff | | gene | 73214322 | | 73217055 | LOC114117520 |
| 13 | 73200001 | 73250000 | 13 | Liftoff | | gene | 73238402 | | 73239910 | SLPI |
| 13 | 78875001 | 78925000 | 13 | Liftoff | | gene | 78900701 | | 79056572 | NFATC2 |
| 14 | 10500001 | 10550000 | 14 | Liftoff | | gene | 10519965 | | 10521432 | LOC114114077 |
| 14 | 23175001 | 23225000 | 14 | Liftoff | | gene | 23178002 | | 23178639 | LOC114116486 |
| 14 | 23175001 | 23225000 | 14 | Liftoff | | gene | 23178310 | | 23517486 | ASAP1 |
| 14 | 32100001 | 32150000 | 14 | Liftoff | | gene | 31988591 | | 32273004 | SPIDR |
| 14 | 32250001 | 32300000 | 14 | Liftoff | | gene | 32285391 | | 32287991 | LOC101111106 |
| 14 | 32275001 | 32325000 | 14 | Liftoff | | gene | 32285391 | | 32287991 | LOC101111106 |
| 14 | 32275001 | 32325000 | 14 | Liftoff | | gene | 32289513 | | 32423993 | PRKDC |
| 14 | 33400001 | 33450000 | 14 | Liftoff | | gene | 33405207 | | 33517662 | LOC101112575 |
| 14 | 33425001 | 33475000 | 14 | Liftoff | | gene | 33405207 | | 33517662 | LOC101112575 |
| 14 | 58350001 | 58400000 | 14 | Liftoff | | gene | 58259852 | | 58436530 | LOC114116379 |
| 14 | 58375001 | 58425000 | 14 | Liftoff | | gene | 58259852 | | 58436530 | LOC114116379 |
| 14 | 75400001 | 75450000 | 14 | Liftoff | | gene | 75381443 | | 75554555 | GRHL2 |
| 14 | 78300001 | 78350000 | 14 | Liftoff | | gene | 78105933 | | 78416249 | STK3 |
| 14 | 78325001 | 78375000 | 14 | Liftoff | | gene | 78105933 | | 78416249 | STK3 |
| 15 | 14675001 | 14725000 | 15 | Liftoff | | gene | 14623717 | | 14704820 | SESN3 |
| 15 | 14850001 | 14900000 | 15 | Liftoff | | gene | 14854789 | | 14856290 | LOC101103347 |
| 15 | 14850001 | 14900000 | 15 | Liftoff | | gene | 14889716 | | 14898242 | CWC15 |
| 15 | 15900001 | 15950000 | 15 | Liftoff | | gene | 15761286 | | 16221539 | GUCY1A2 |
| 15 | 15925001 | 15975000 | 15 | Liftoff | | gene | 15761286 | | 16221539 | GUCY1A2 |
| 15 | 27550001 | 27600000 | 15 | Liftoff | | gene | 27523995 | | 27888515 | DSCAML1 |
| 15 | 27950001 | 28000000 | 15 | Liftoff | | gene | 27924144 | | 27960808 | FXYD6 |
| 15 | 27975001 | 28025000 | 15 | Liftoff | | gene | 28007746 | | 28040922 | TMPRSS13 |
| 15 | 31075001 | 31125000 | 15 | Liftoff | | gene | 31064043 | | 31136306 | TBCEL |
| 15 | 31100001 | 31150000 | 15 | Liftoff | | gene | 31064043 | | 31136306 | TBCEL |
| 15 | 325001 | 375000 | 15 | Liftoff | | gene | 353228 | | 354160 | LOC101113787 |
| 15 | 44650001 | 44700000 | 15 | Liftoff | | gene | 44603428 | | 44814593 | SYT9 |
| 15 | 47900001 | 47950000 | 15 | Liftoff | | gene | 47882374 | | 47907469 | LOC114118414 |
| 15 | 47900001 | 47950000 | 15 | Liftoff | | gene | 47935716 | | 47945851 | LOC114118421 |
| 15 | 53475001 | 53525000 | 15 | Liftoff | | gene | 53420048 | | 53490090 | XRRA1 |
| 15 | 53475001 | 53525000 | 15 | Liftoff | | gene | 53490245 | | 53516833 | SPCS2 |
| 15 | 53500001 | 53550000 | 15 | Liftoff | | gene | 53490245 | | 53516833 | SPCS2 |
| 15 | 53500001 | 53550000 | 15 | Liftoff | | gene | 53532116 | | 53546689 | NEU3 |
| 15 | 54425001 | 54475000 | 15 | Liftoff | | gene | 54405403 | | 54435898 | LOC101108161 |
| 15 | 54450001 | 54500000 | 15 | Liftoff | | gene | 54495472 | | 54505400 | LOC101110266 |
| 15 | 54550001 | 54600000 | 15 | Liftoff | | gene | 54540522 | | 54573556 | DGAT2 |
| 15 | 61525001 | 61575000 | 15 | Liftoff | | gene | 61528626 | | 61605431 | DNAJC24 |
| 15 | 61550001 | 61600000 | 15 | Liftoff | | gene | 61528626 | | 61605431 | DNAJC24 |
| 15 | 62725001 | 62775000 | 15 | Liftoff | | gene | 62683365 | | 62833809 | CCDC73 |
| 15 | 62750001 | 62800000 | 15 | Liftoff | | gene | 62683365 | | 62833809 | CCDC73 |
| 15 | 62775001 | 62825000 | 15 | Liftoff | | gene | 62683365 | | 62833809 | CCDC73 |
| 15 | 63650001 | 63700000 | 15 | Liftoff | | gene | 63684779 | | 63686680 | C15H11orf91 |
| 15 | 64225001 | 64275000 | 15 | Liftoff | | gene | 64105373 | | 64287960 | ABTB2 |
| 15 | 64350001 | 64400000 | 15 | Liftoff | | gene | 64329032 | | 64365350 | CAT |
| 15 | 64350001 | 64400000 | 15 | Liftoff | | gene | 64373727 | | 64406684 | ELF5 |
| 15 | 64375001 | 64425000 | 15 | Liftoff | | gene | 64373727 | | 64406684 | ELF5 |
| 15 | 80300001 | 80350000 | 15 | Liftoff | | gene | 80296703 | | 80306114 | TMX2 |
| 15 | 80300001 | 80350000 | 15 | Liftoff | | gene | 80307165 | | 80308845 | SELENOH |
| 15 | 80300001 | 80350000 | 15 | Liftoff | | gene | 80309459 | | 80315573 | BTBD18 |
| 15 | 80300001 | 80350000 | 15 | Liftoff | | gene | 80323159 | | 80368249 | CTNND1 |
| 16 | 1025001 | 1075000 | 16 | Liftoff | | gene | 1028960 | | 1030021 | LOC106990697 |
| 16 | 1875001 | 1925000 | 16 | Liftoff | | gene | 1910610 | | 1914724 | LOC114117315 |
| 16 | 1900001 | 1950000 | 16 | Liftoff | | gene | 1910610 | | 1914724 | LOC114117315 |
| 16 | 1925001 | 1975000 | 16 | Liftoff | | gene | 1955157 | | 1959308 | LOC114117324 |
| 16 | 36275001 | 36325000 | 16 | Liftoff | | gene | 36242439 | | 36279158 | DPT |
| 16 | 49925001 | 49975000 | 16 | Liftoff | | gene | 49936068 | | 49939084 | LOC105616533 |
| 16 | 49950001 | 50000000 | 16 | Liftoff | | gene | 49976715 | | 49977848 | ACTRT2 |
| 1 | 6500001 | 6550000 | 1 | Liftoff | | gene | 6518980 | | 6528779 | LOC105610396 |
| 1 | 6500001 | 6550000 | 1 | Liftoff | | gene | 6528960 | | 6542543 | CCT8 |
| 1 | 6500001 | 6550000 | 1 | Liftoff | | gene | 6543706 | | 6569951 | USP16 |
| 16 | 50425001 | 50475000 | 16 | Liftoff | | gene | 50392909 | | 50443606 | MORN1 |
| 16 | 50425001 | 50475000 | 16 | Liftoff | | gene | 50454663 | | 50508835 | SKI |
| 1 | 6525001 | 6575000 | 1 | Liftoff | | gene | 6518980 | | 6528779 | LOC105610396 |
| 1 | 6525001 | 6575000 | 1 | Liftoff | | gene | 6528960 | | 6542543 | CCT8 |
| 1 | 6525001 | 6575000 | 1 | Liftoff | | gene | 6543706 | | 6569951 | USP16 |
| 1 | 6525001 | 6575000 | 1 | Liftoff | | gene | 6572920 | | 6586523 | RWDD2B |
| 16 | 55000001 | 55050000 | 16 | Liftoff | | gene | 54941604 | | 55035613 | SLC9C2 |
| 16 | 55000001 | 55050000 | 16 | Liftoff | | gene | 55043854 | | 55089474 | ANKRD45 |
| 16 | 64100001 | 64150000 | 16 | Liftoff | | gene | 64101594 | | 64114242 | LOC114117300 |
| 16 | 64100001 | 64150000 | 16 | Liftoff | | gene | 64128900 | | 64250642 | LAMC1 |
| 16 | 67750001 | 67800000 | 16 | Liftoff | | gene | 67790031 | | 67797663 | PTGS2 |
| 16 | 79725001 | 79775000 | 16 | Liftoff | | gene | 79701260 | | 79731200 | IGFN1 |
| 16 | 79725001 | 79775000 | 16 | Liftoff | | gene | 79770447 | | 79811435 | PKP1 |
| 17 | 10775001 | 10825000 | 17 | Liftoff | | gene | 10810346 | | 10885209 | EDNRA |
| 17 | 12225001 | 12275000 | 17 | Liftoff | | gene | 12230277 | | 12248239 | REELD1 |
| 17 | 40000001 | 40050000 | 17 | Liftoff | | gene | 39884925 | | 40148161 | RAPGEF2 |
| 17 | 40200001 | 40250000 | 17 | Liftoff | | gene | 40228047 | | 40346435 | C17H4orf45 |
| 17 | 44500001 | 44550000 | 17 | Liftoff | | gene | 44481291 | | 44527945 | GOLGA3 |
| 17 | 44500001 | 44550000 | 17 | Liftoff | | gene | 44534812 | | 44559143 | ANKLE2 |
| 17 | 44650001 | 44700000 | 17 | Liftoff | | gene | 44652789 | | 44652895 | LOC114118784 |
| 17 | 44650001 | 44700000 | 17 | Liftoff | | gene | 44654273 | | 44664802 | LRCOL1 |
| 17 | 44650001 | 44700000 | 17 | Liftoff | | gene | 44673337 | | 44757317 | FBRSL1 |
| 17 | 44925001 | 44975000 | 17 | Liftoff | | gene | 44904407 | | 45025472 | GALNT9 |
| 17 | 46100001 | 46150000 | 17 | Liftoff | | gene | 46111856 | | 46288055 | ADGRD1 |
| 17 | 46125001 | 46175000 | 17 | Liftoff | | gene | 46111856 | | 46288055 | ADGRD1 |
| 17 | 52775001 | 52825000 | 17 | Liftoff | | gene | 52741064 | | 52782254 | CCDC62 |
| 17 | 52775001 | 52825000 | 17 | Liftoff | | gene | 52785334 | | 52798528 | DENR |
| 17 | 52775001 | 52825000 | 17 | Liftoff | | gene | 52788995 | | 52789124 | LOC114108881 |
| 17 | 52775001 | 52825000 | 17 | Liftoff | | gene | 52824003 | | 52828005 | HCAR1 |
| 17 | 52800001 | 52850000 | 17 | Liftoff | | gene | 52824003 | | 52828005 | HCAR1 |
| 17 | 52800001 | 52850000 | 17 | Liftoff | | gene | 52840874 | | 52843606 | HCAR2 |
| 17 | 55325001 | 55375000 | 17 | Liftoff | | gene | 55330098 | | 55361087 | BRAP |
| 17 | 55325001 | 55375000 | 17 | Liftoff | | gene | 55361188 | | 55399375 | LOC101105533 |
| 17 | 55325001 | 55375000 | 17 | Liftoff | | gene | 55364431 | | 55375318 | LOC105602881 |
| 17 | 61175001 | 61225000 | 17 | Liftoff | | gene | 61176478 | | 61214695 | DTX1 |
| 17 | 61175001 | 61225000 | 17 | Liftoff | | gene | 61215682 | | 61247955 | RASAL1 |
| 17 | 61175001 | 61225000 | 17 | Liftoff | | gene | 61219750 | | 61219820 | TRNAG-GCC-25 |
| 17 | 62500001 | 62550000 | 17 | Liftoff | | gene | 62434875 | | 62533111 | LOC105602913 |
| 17 | 62500001 | 62550000 | 17 | Liftoff | | gene | 62534025 | | 62613614 | BICDL1 |
| 17 | 62525001 | 62575000 | 17 | Liftoff | | gene | 62534025 | | 62613614 | BICDL1 |
| 17 | 68675001 | 68725000 | 17 | Liftoff | | gene | 68694904 | | 68701998 | NEFH |
| 17 | 68675001 | 68725000 | 17 | Liftoff | | gene | 68710763 | | 68740688 | THOC5 |
| 17 | 70400001 | 70450000 | 17 | Liftoff | | gene | 70398417 | | 70408118 | YWHAH |
| 17 | 70425001 | 70475000 | 17 | Liftoff | | gene | 70458981 | | 70459087 | LOC114108917 |
| 1 | 80350001 | 80400000 | 1 | Liftoff | | gene | 80364552 | | 80589677 | DGKG |
| 18 | 13425001 | 13475000 | 18 | Liftoff | | gene | 13429594 | | 13434802 | CDT1 |
| 18 | 13425001 | 13475000 | 18 | Liftoff | | gene | 13434960 | | 13437588 | APRT |
| 18 | 13425001 | 13475000 | 18 | Liftoff | | gene | 13438220 | | 13454382 | GALNS |
| 18 | 13425001 | 13475000 | 18 | Liftoff | | gene | 13454385 | | 13459486 | TRAPPC2L |
| 18 | 13425001 | 13475000 | 18 | Liftoff | | gene | 13461887 | | 13465289 | PABPN1L |
| 18 | 13450001 | 13500000 | 18 | Liftoff | | gene | 13438220 | | 13454382 | GALNS |
| 18 | 13450001 | 13500000 | 18 | Liftoff | | gene | 13454385 | | 13459486 | TRAPPC2L |
| 18 | 13450001 | 13500000 | 18 | Liftoff | | gene | 13461887 | | 13465289 | PABPN1L |
| 18 | 13450001 | 13500000 | 18 | Liftoff | | gene | 13467566 | | 13548761 | CBFA2T3 |
| 18 | 13475001 | 13525000 | 18 | Liftoff | | gene | 13467566 | | 13548761 | CBFA2T3 |
| 18 | 1550001 | 1600000 | 18 | Liftoff | | gene | 1555108 | | 1609890 | FA2H |
| 18 | 17850001 | 17900000 | 18 | Liftoff | | gene | 17860178 | | 17896389 | HEATR3 |
| 18 | 24400001 | 24450000 | 18 | Liftoff | | gene | 24390494 | | 24481894 | NLRC5 |
| 1 | 82575001 | 82625000 | 1 | Liftoff | | gene | 82602072 | | 82620527 | LOC101108715 |
| 1 | 82600001 | 82650000 | 1 | Liftoff | | gene | 82602072 | | 82620527 | LOC101108715 |
| 1 | 82600001 | 82650000 | 1 | Liftoff | | gene | 82620362 | | 82703053 | ABCC5 |
| 18 | 2800001 | 2850000 | 18 | Liftoff | | gene | 2805557 | | 3086222 | CNTNAP4 |
| 18 | 2825001 | 2875000 | 18 | Liftoff | | gene | 2805557 | | 3086222 | CNTNAP4 |
| 18 | 34475001 | 34525000 | 18 | Liftoff | | gene | 34482459 | | 34494395 | CARMIL2 |
| 18 | 34475001 | 34525000 | 18 | Liftoff | | gene | 34494358 | | 34500590 | ACD |
| 18 | 34475001 | 34525000 | 18 | Liftoff | | gene | 34501022 | | 34503192 | PARD6A |
| 18 | 34475001 | 34525000 | 18 | Liftoff | | gene | 34503379 | | 34506849 | ENKD1 |
| 18 | 34475001 | 34525000 | 18 | Liftoff | | gene | 34506983 | | 34509002 | C14H16orf86 |
| 18 | 34475001 | 34525000 | 18 | Liftoff | | gene | 34511532 | | 34553372 | GFOD2 |
| 18 | 34500001 | 34550000 | 18 | Liftoff | | gene | 34501022 | | 34503192 | PARD6A |
| 18 | 34500001 | 34550000 | 18 | Liftoff | | gene | 34503379 | | 34506849 | ENKD1 |
| 18 | 34500001 | 34550000 | 18 | Liftoff | | gene | 34506983 | | 34509002 | C14H16orf86 |
| 18 | 34500001 | 34550000 | 18 | Liftoff | | gene | 34511532 | | 34553372 | GFOD2 |
| 1 | 83500001 | 83550000 | 1 | Liftoff | | gene | 83482478 | | 83535741 | MCCC1 |
| 18 | 42800001 | 42850000 | 18 | Liftoff | | gene | 42807818 | | 42807891 | TRNAT-AGU-6 |
| 18 | 42800001 | 42850000 | 18 | Liftoff | | gene | 42839205 | | 42851015 | LRP3 |
| 18 | 49200001 | 49250000 | 18 | Liftoff | | gene | 49206344 | | 49208268 | LOC114117815 |
| 18 | 49225001 | 49275000 | 18 | Liftoff | | gene | 49249431 | | 49278686 | LOC105608805 |
| 18 | 49250001 | 49300000 | 18 | Liftoff | | gene | 49249431 | | 49278686 | LOC105608805 |
| 18 | 49275001 | 49325000 | 18 | Liftoff | | gene | 49249431 | | 49278686 | LOC105608805 |
| 18 | 50200001 | 50250000 | 18 | Liftoff | | gene | 50180380 | | 50221688 | MEGF8 |
| 18 | 50200001 | 50250000 | 18 | Liftoff | | gene | 50222101 | | 50232020 | TMEM145 |
| 18 | 50200001 | 50250000 | 18 | Liftoff | | gene | 50233756 | | 50236791 | PRR19 |
| 18 | 50200001 | 50250000 | 18 | Liftoff | | gene | 50238332 | | 50241322 | PAFAH1B3 |
| 18 | 50200001 | 50250000 | 18 | Liftoff | | gene | 50242120 | | 50269855 | CIC |
| 18 | 50225001 | 50275000 | 18 | Liftoff | | gene | 50222101 | | 50232020 | TMEM145 |
| 18 | 50225001 | 50275000 | 18 | Liftoff | | gene | 50233756 | | 50236791 | PRR19 |
| 18 | 50225001 | 50275000 | 18 | Liftoff | | gene | 50238332 | | 50241322 | PAFAH1B3 |
| 18 | 50225001 | 50275000 | 18 | Liftoff | | gene | 50242120 | | 50269855 | CIC |
| 18 | 50250001 | 50300000 | 18 | Liftoff | | gene | 50242120 | | 50269855 | CIC |
| 18 | 50250001 | 50300000 | 18 | Liftoff | | gene | 50281706 | | 50289290 | ERF |
| 18 | 50250001 | 50300000 | 18 | Liftoff | | gene | 50293284 | | 50302514 | GSK3A |
| 18 | 50350001 | 50400000 | 18 | Liftoff | | gene | 50394605 | | 50430169 | POU2F2 |
| 18 | 50375001 | 50425000 | 18 | Liftoff | | gene | 50394605 | | 50430169 | POU2F2 |
| 18 | 52425001 | 52475000 | 18 | Liftoff | | gene | 52426783 | | 52442549 | PPP1R13L |
| 18 | 52425001 | 52475000 | 18 | Liftoff | | gene | 52442720 | | 52445468 | CD3EAP |
| 18 | 52425001 | 52475000 | 18 | Liftoff | | gene | 52445298 | | 52459940 | ERCC1 |
| 18 | 52425001 | 52475000 | 18 | Liftoff | | gene | 52465929 | | 52467357 | LOC105608454 |
| 18 | 52425001 | 52475000 | 18 | Liftoff | | gene | 52465969 | | 52499168 | LOC105616901 |
| 18 | 60175001 | 60225000 | 18 | Liftoff | | gene | 60184480 | | 60217995 | LOC114118036 |
| 18 | 60175001 | 60225000 | 18 | Liftoff | | gene | 60190365 | | 60190471 | LOC114118142 |
| 18 | 60200001 | 60250000 | 18 | Liftoff | | gene | 60184480 | | 60217995 | LOC114118036 |
| 1 | 86900001 | 86950000 | 1 | Liftoff | | gene | 86815652 | | 86951136 | USP13 |
| 18 | 7125001 | 7175000 | 18 | Liftoff | | gene | 7124481 | | 7135083 | C14H16orf46 |
| 18 | 7125001 | 7175000 | 18 | Liftoff | | gene | 7136609 | | 7136712 | LOC114118160 |
| 18 | 7125001 | 7175000 | 18 | Liftoff | | gene | 7139993 | | 7152029 | GCSH |
| 18 | 7125001 | 7175000 | 18 | Liftoff | | gene | 7151649 | | 7176495 | LOC114108669 |
| 18 | 7125001 | 7175000 | 18 | Liftoff | | gene | 7156316 | | 7257222 | PKD1L2 |
| 18 | 8075001 | 8125000 | 18 | Liftoff | | gene | 8107699 | | 8118310 | MPHOSPH6 |
| 18 | 8100001 | 8150000 | 18 | Liftoff | | gene | 8107699 | | 8118310 | MPHOSPH6 |
| 19 | 11925001 | 11975000 | 19 | Liftoff | | gene | 11922615 | | 11982626 | PPM1D |
| 19 | 11950001 | 12000000 | 19 | Liftoff | | gene | 11922615 | | 11982626 | PPM1D |
| 19 | 12325001 | 12375000 | 19 | Liftoff | | gene | 12199757 | | 12397026 | USP32 |
| 19 | 12350001 | 12400000 | 19 | Liftoff | | gene | 12199757 | | 12397026 | USP32 |
| 19 | 21875001 | 21925000 | 19 | Liftoff | | gene | 21823122 | | 21982691 | NXN |
| 19 | 22850001 | 22900000 | 19 | Liftoff | | gene | 22823144 | | 22866564 | RPA1 |
| 19 | 24100001 | 24150000 | 19 | Liftoff | | gene | 24115180 | | 24116124 | LOC101120929 |
| 19 | 24100001 | 24150000 | 19 | Liftoff | | gene | 24123380 | | 24136488 | SPATA22 |
| 19 | 24100001 | 24150000 | 19 | Liftoff | | gene | 24139579 | | 24161887 | ASPA |
| 19 | 26000001 | 26050000 | 19 | Liftoff | | gene | 26004466 | | 26006310 | LOC105616457 |
| 19 | 26000001 | 26050000 | 19 | Liftoff | | gene | 26007559 | | 26012601 | MIS12 |
| 19 | 26000001 | 26050000 | 19 | Liftoff | | gene | 26012792 | | 26021379 | DERL2 |
| 19 | 26000001 | 26050000 | 19 | Liftoff | | gene | 26029638 | | 26051938 | DHX33 |
| 19 | 34850001 | 34900000 | 19 | Liftoff | | gene | 34873935 | | 34874069 | LOC114109487 |
| 19 | 34850001 | 34900000 | 19 | Liftoff | | gene | 34887582 | | 34910492 | TNFRSF13B |
| 19 | 48875001 | 48925000 | 19 | Liftoff | | gene | 48893611 | | 48913121 | NOL11 |
| 19 | 48875001 | 48925000 | 19 | Liftoff | | gene | 48913864 | | 48913945 | TRNAS-AGA-6 |
| 19 | 48900001 | 48950000 | 19 | Liftoff | | gene | 48893611 | | 48913121 | NOL11 |
| 19 | 48900001 | 48950000 | 19 | Liftoff | | gene | 48913864 | | 48913945 | TRNAS-AGA-6 |
| 19 | 51350001 | 51400000 | 19 | Liftoff | | gene | 51185792 | | 51510466 | RPTOR |
| 19 | 51975001 | 52025000 | 19 | Liftoff | | gene | 51977355 | | 51989190 | EIF4A3 |
| 19 | 51975001 | 52025000 | 19 | Liftoff | | gene | 51991606 | | 52007651 | GAA |
| 19 | 51975001 | 52025000 | 19 | Liftoff | | gene | 52008863 | | 52056273 | CCDC40 |
| 19 | 52000001 | 52050000 | 19 | Liftoff | | gene | 51991606 | | 52007651 | GAA |
| 19 | 52000001 | 52050000 | 19 | Liftoff | | gene | 52008863 | | 52056273 | CCDC40 |
| 19 | 52250001 | 52300000 | 19 | Liftoff | | gene | 52254023 | | 52258919 | CBX8 |
| 19 | 52250001 | 52300000 | 19 | Liftoff | | gene | 52262417 | | 52271751 | CBX2 |
| 19 | 53400001 | 53450000 | 19 | Liftoff | | gene | 53401980 | | 53406449 | LOC105606280 |
| 19 | 53400001 | 53450000 | 19 | Liftoff | | gene | 53415236 | | 53421183 | LOC101113775 |
| 19 | 53400001 | 53450000 | 19 | Liftoff | | gene | 53424074 | | 53430478 | TMEM235 |
| 19 | 53400001 | 53450000 | 19 | Liftoff | | gene | 53435026 | | 53443123 | BIRC5 |
| 19 | 53400001 | 53450000 | 19 | Liftoff | | gene | 53447279 | | 53469445 | AFMID |
| 19 | 53575001 | 53625000 | 19 | Liftoff | | gene | 53542318 | | 53615343 | TNRC6C |
| 19 | 53600001 | 53650000 | 19 | Liftoff | | gene | 53542318 | | 53615343 | TNRC6C |
| 1 | 96700001 | 96750000 | 1 | Liftoff | | gene | 96724684 | | 96758087 | SEC62 |
| 20 | 14700001 | 14750000 | 20 | Liftoff | | gene | 14501476 | | 14786505 | ADAMTS6 |
| 20 | 14900001 | 14950000 | 20 | Liftoff | | gene | 14904591 | | 14911277 | LOC114118733 |
| 20 | 14900001 | 14950000 | 20 | Liftoff | | gene | 14918775 | | 15173813 | CWC27 |
| 20 | 15650001 | 15700000 | 20 | Liftoff | | gene | 15608229 | | 15799551 | RNF180 |
| 20 | 15675001 | 15725000 | 20 | Liftoff | | gene | 15608229 | | 15799551 | RNF180 |
| 20 | 16775001 | 16825000 | 20 | Liftoff | | gene | 16751394 | | 17026051 | LOC106991659 |
| 20 | 17250001 | 17300000 | 20 | Liftoff | | gene | 17251264 | | 17443505 | IPO11 |
| 20 | 17250001 | 17300000 | 20 | Liftoff | | gene | 17293976 | | 17297014 | LRRC70 |
| 20 | 17275001 | 17325000 | 20 | Liftoff | | gene | 17251264 | | 17443505 | IPO11 |
| 20 | 17275001 | 17325000 | 20 | Liftoff | | gene | 17293976 | | 17297014 | LRRC70 |
| 20 | 19000001 | 19050000 | 20 | Liftoff | | gene | 19027515 | | 19038319 | LOC114118678 |
| 20 | 19025001 | 19075000 | 20 | Liftoff | | gene | 19027515 | | 19038319 | LOC114118678 |
| 20 | 19075001 | 19125000 | 20 | Liftoff | | gene | 19117288 | | 19129048 | LOC114118679 |
| 20 | 22750001 | 22800000 | 20 | Liftoff | | gene | 22773715 | | 22802366 | MIER3 |
| 20 | 22775001 | 22825000 | 20 | Liftoff | | gene | 22773715 | | 22802366 | MIER3 |
| 20 | 22775001 | 22825000 | 20 | Liftoff | | gene | 22807788 | | 22815884 | SETD9 |
| 20 | 22850001 | 22900000 | 20 | Liftoff | | gene | 22832313 | | 22906777 | MAP3K1 |
| 20 | 29775001 | 29825000 | 20 | Liftoff | | gene | 29553067 | | 30003897 | HCN1 |
| 20 | 32475001 | 32525000 | 20 | Liftoff | | gene | 32306971 | | 32602644 | GHR |
| 20 | 32475001 | 32525000 | 20 | Liftoff | | gene | 32489925 | | 32519587 | LOC114118741 |
| 20 | 3975001 | 4025000 | 20 | Liftoff | | gene | 3959775 | | 4132728 | SMIM23 |
| 20 | 41275001 | 41325000 | 20 | Liftoff | | gene | 41277058 | | 41355607 | NPR3 |
| 20 | 41300001 | 41350000 | 20 | Liftoff | | gene | 41277058 | | 41355607 | NPR3 |
| 20 | 56550001 | 56600000 | 20 | Liftoff | | gene | 56560971 | | 56821095 | MYO10 |
| 20 | 56575001 | 56625000 | 20 | Liftoff | | gene | 56560971 | | 56821095 | MYO10 |
| 20 | 57025001 | 57075000 | 20 | Liftoff | | gene | 57016691 | | 57030505 | ZNF622 |
| 20 | 57325001 | 57375000 | 20 | Liftoff | | gene | 57282938 | | 57399276 | 11-Mar |
| 20 | 57900001 | 57950000 | 20 | Liftoff | | gene | 57549977 | | 58001895 | FBXL7 |
| 20 | 57925001 | 57975000 | 20 | Liftoff | | gene | 57549977 | | 58001895 | FBXL7 |
| 20 | 63050001 | 63100000 | 20 | Liftoff | | gene | 63041931 | | 63060396 | ROPN1L |
| 20 | 63050001 | 63100000 | 20 | Liftoff | | gene | 63065093 | | 63139792 | 6-Mar |
| 20 | 63100001 | 63150000 | 20 | Liftoff | | gene | 63065093 | | 63139792 | 6-Mar |
| 20 | 825001 | 875000 | 20 | Liftoff | | gene | 829478 | | 852730 | PANK3 |
| 2 | 113450001 | 113500000 | 2 | Liftoff | | gene | 113428089 | | 113535333 | CUL3 |
| 21 | 19200001 | 19250000 | 21 | Liftoff | | gene | 19215136 | | 19219780 | LOC101118056 |
| 21 | 19200001 | 19250000 | 21 | Liftoff | | gene | 19226177 | | 19234541 | LOC105603085 |
| 21 | 19625001 | 19675000 | 21 | Liftoff | | gene | 19650494 | | 19653241 | LOC114109097 |
| 21 | 19625001 | 19675000 | 21 | Liftoff | | gene | 19655881 | | 19658695 | LOC101122633 |
| 2 | 119925001 | 119975000 | 2 | Liftoff | | gene | 119837258 | | 120019982 | ARMC9 |
| 21 | 22275001 | 22325000 | 21 | Liftoff | | gene | 22230598 | | 22340262 | CPEB1 |
| 21 | 22300001 | 22350000 | 21 | Liftoff | | gene | 22230598 | | 22340262 | CPEB1 |
| 21 | 24225001 | 24275000 | 21 | Liftoff | | gene | 24270631 | | 24297861 | BNC1 |
| 21 | 25275001 | 25325000 | 21 | Liftoff | | gene | 25273304 | | 25295079 | CTSH |
| 21 | 25275001 | 25325000 | 21 | Liftoff | | gene | 25313216 | | 25415246 | RASGRF1 |
| 2 | 126125001 | 126175000 | 2 | Liftoff | | gene | 126150428 | | 126168664 | PPP1R8 |
| 2 | 126125001 | 126175000 | 2 | Liftoff | | gene | 126164827 | | 126164992 | LOC114113410 |
| 2 | 126200001 | 126250000 | 2 | Liftoff | | gene | 126172407 | | 126210226 | STX12 |
| 2 | 126200001 | 126250000 | 2 | Liftoff | | gene | 126230282 | | 126265014 | FAM76A |
| 21 | 28475001 | 28525000 | 21 | Liftoff | | gene | 28505520 | | 28617872 | TJP1 |
| 21 | 28500001 | 28550000 | 21 | Liftoff | | gene | 28505520 | | 28617872 | TJP1 |
| 2 | 128575001 | 128625000 | 2 | Liftoff | | gene | 128598513 | | 128615889 | LOC114112907 |
| 21 | 28575001 | 28625000 | 21 | Liftoff | | gene | 28505520 | | 28617872 | TJP1 |
| 2 | 128600001 | 128650000 | 2 | Liftoff | | gene | 128598513 | | 128615889 | LOC114112907 |
| 2 | 128600001 | 128650000 | 2 | Liftoff | | gene | 128642273 | | 128709673 | RUNX3 |
| 2 | 128825001 | 128875000 | 2 | Liftoff | | gene | 128758857 | | 128840018 | CLIC4 |
| 2 | 128825001 | 128875000 | 2 | Liftoff | | gene | 128859695 | | 128861430 | LOC114112908 |
| 2 | 134325001 | 134375000 | 2 | Liftoff | | gene | 134285296 | | 134336564 | IFFO2 |
| 2 | 134325001 | 134375000 | 2 | Liftoff | | gene | 134337939 | | 134370183 | ALDH4A1 |
| 21 | 34450001 | 34500000 | 21 | Liftoff | | gene | 34481582 | | 34489985 | ISLR2 |
| 21 | 35125001 | 35175000 | 21 | Liftoff | | gene | 35143645 | | 35413907 | STXBP6 |
| 21 | 35175001 | 35225000 | 21 | Liftoff | | gene | 35143645 | | 35413907 | STXBP6 |
| 21 | 43025001 | 43075000 | 21 | Liftoff | | gene | 42893374 | | 43289633 | AKAP6 |
| 21 | 44600001 | 44650000 | 21 | Liftoff | | gene | 44565272 | | 44920830 | LOC105603181 |
| 21 | 46550001 | 46600000 | 21 | Liftoff | | gene | 46467305 | | 46561136 | LOC106991743 |
| 21 | 46550001 | 46600000 | 21 | Liftoff | | gene | 46562190 | | 46581359 | MBIP |
| 21 | 48000001 | 48050000 | 21 | Liftoff | | gene | 47873084 | | 48075377 | TTC6 |
| 21 | 48025001 | 48075000 | 21 | Liftoff | | gene | 47873084 | | 48075377 | TTC6 |
| 21 | 54850001 | 54900000 | 21 | Liftoff | | gene | 54850621 | | 54884037 | KLHL28 |
| 21 | 54850001 | 54900000 | 21 | Liftoff | | gene | 54884290 | | 54960301 | TOGARAM1 |
| 21 | 58225001 | 58275000 | 21 | Liftoff | | gene | 58206189 | | 58422166 | UNC79 |
| 21 | 58250001 | 58300000 | 21 | Liftoff | | gene | 58206189 | | 58422166 | UNC79 |
| 21 | 58275001 | 58325000 | 21 | Liftoff | | gene | 58206189 | | 58422166 | UNC79 |
| 21 | 58300001 | 58350000 | 21 | Liftoff | | gene | 58206189 | | 58422166 | UNC79 |
| 21 | 67675001 | 67725000 | 21 | Liftoff | | gene | 67683921 | | 67686528 | LOC105603273 |
| 21 | 67675001 | 67725000 | 21 | Liftoff | | gene | 67694422 | | 67697354 | LBHD2 |
| 21 | 67675001 | 67725000 | 21 | Liftoff | | gene | 67707132 | | 67717847 | EXOC3L4 |
| 21 | 67675001 | 67725000 | 21 | Liftoff | | gene | 67722457 | | 67733425 | LOC105605780 |
| 21 | 67725001 | 67775000 | 21 | Liftoff | | gene | 67722457 | | 67733425 | LOC105605780 |
| 21 | 67725001 | 67775000 | 21 | Liftoff | | gene | 67747243 | | 67778232 | LOC114109056 |
| 21 | 67750001 | 67800000 | 21 | Liftoff | | gene | 67747243 | | 67778232 | LOC114109056 |
| 21 | 67750001 | 67800000 | 21 | Liftoff | | gene | 67786953 | | 67800609 | LOC105605761 |
| 21 | 67775001 | 67825000 | 21 | Liftoff | | gene | 67747243 | | 67778232 | LOC114109056 |
| 21 | 67775001 | 67825000 | 21 | Liftoff | | gene | 67786953 | | 67800609 | LOC105605761 |
| 21 | 67925001 | 67975000 | 21 | Liftoff | | gene | 67933036 | | 67936431 | LOC114109084 |
| 21 | 67925001 | 67975000 | 21 | Liftoff | | gene | 67936702 | | 67938348 | LOC101104530 |
| 21 | 67925001 | 67975000 | 21 | Liftoff | | gene | 67956757 | | 67959276 | LOC105603310 |
| 21 | 67925001 | 67975000 | 21 | Liftoff | | gene | 67959323 | | 67961468 | LOC114109058 |
| 21 | 67925001 | 67975000 | 21 | Liftoff | | gene | 67963824 | | 67965269 | LOC105603275 |
| 21 | 67950001 | 68000000 | 21 | Liftoff | | gene | 67956757 | | 67959276 | LOC105603310 |
| 21 | 67950001 | 68000000 | 21 | Liftoff | | gene | 67959323 | | 67961468 | LOC114109058 |
| 21 | 67950001 | 68000000 | 21 | Liftoff | | gene | 67963824 | | 67965269 | LOC105603275 |
| 22 | 1000001 | 1050000 | 22 | Liftoff | | gene | 920225 | | 1132838 | EGFR |
| 22 | 1025001 | 1075000 | 22 | Liftoff | | gene | 920225 | | 1132838 | EGFR |
| 22 | 10600001 | 10650000 | 22 | Liftoff | | gene | 10607262 | | 10615012 | EPM2AIP1 |
| 22 | 11450001 | 11500000 | 22 | Liftoff | | gene | 11366159 | | 11490247 | CTDSPL |
| 22 | 11450001 | 11500000 | 22 | Liftoff | | gene | 11459372 | | 11462487 | LOC105603371 |
| 22 | 11450001 | 11500000 | 22 | Liftoff | | gene | 11495619 | | 11515898 | VILL |
| 22 | 1850001 | 1900000 | 22 | Liftoff | | gene | 1808236 | | 1932921 | SLC4A7 |
| 22 | 21575001 | 21625000 | 22 | Liftoff | | gene | 21528054 | | 21879057 | ITPR1 |
| 22 | 21600001 | 21650000 | 22 | Liftoff | | gene | 21528054 | | 21879057 | ITPR1 |
| 22 | 23200001 | 23250000 | 22 | Liftoff | | gene | 23204775 | | 23242105 | IL5RA |
| 22 | 23225001 | 23275000 | 22 | Liftoff | | gene | 23204775 | | 23242105 | IL5RA |
| 22 | 23225001 | 23275000 | 22 | Liftoff | | gene | 23254949 | | 23354521 | CNTN4 |
| 22 | 2700001 | 2750000 | 22 | Liftoff | | gene | 2730503 | | 2812689 | CMC1 |
| 22 | 2725001 | 2775000 | 22 | Liftoff | | gene | 2730503 | | 2812689 | CMC1 |
| 22 | 2750001 | 2800000 | 22 | Liftoff | | gene | 2730503 | | 2812689 | CMC1 |
| 22 | 2775001 | 2825000 | 22 | Liftoff | | gene | 2730503 | | 2812689 | CMC1 |
| 22 | 2775001 | 2825000 | 22 | Liftoff | | gene | 2817276 | | 2852514 | AZI2 |
| 22 | 35200001 | 35250000 | 22 | Liftoff | | gene | 35179254 | | 35320734 | SLC25A26 |
| 22 | 35225001 | 35275000 | 22 | Liftoff | | gene | 35179254 | | 35320734 | SLC25A26 |
| 2 | 24750001 | 24800000 | 2 | Liftoff | | gene | 24786446 | | 24788514 | DLX2 |
| 2 | 24775001 | 24825000 | 2 | Liftoff | | gene | 24786446 | | 24788514 | DLX2 |
| 2 | 24775001 | 24825000 | 2 | Liftoff | | gene | 24800967 | | 24803522 | DLX1 |
| 2 | 24775001 | 24825000 | 2 | Liftoff | | gene | 24808290 | | 24881878 | METAP1D |
| 22 | 48100001 | 48150000 | 22 | Liftoff | | gene | 48101186 | | 48115559 | ITIH1 |
| 22 | 48100001 | 48150000 | 22 | Liftoff | | gene | 48127239 | | 48156903 | NEK4 |
| 22 | 48100001 | 48150000 | 22 | Liftoff | | gene | 48135766 | | 48135891 | LOC114109447 |
| 22 | 48125001 | 48175000 | 22 | Liftoff | | gene | 48127239 | | 48156903 | NEK4 |
| 22 | 48125001 | 48175000 | 22 | Liftoff | | gene | 48135766 | | 48135891 | LOC114109447 |
| 22 | 48125001 | 48175000 | 22 | Liftoff | | gene | 48158708 | | 48160647 | SPCS1 |
| 22 | 48125001 | 48175000 | 22 | Liftoff | | gene | 48163610 | | 48170790 | GLT8D1 |
| 22 | 48125001 | 48175000 | 22 | Liftoff | | gene | 48170866 | | 48177550 | GNL3 |
| 22 | 48125001 | 48175000 | 22 | Liftoff | | gene | 48172491 | | 48172567 | LOC114109492 |
| 22 | 48125001 | 48175000 | 22 | Liftoff | | gene | 48173555 | | 48173636 | LOC114109448 |
| 22 | 48125001 | 48175000 | 22 | Liftoff | | gene | 48174373 | | 48174449 | LOC114109491 |
| 22 | 52625001 | 52675000 | 22 | Liftoff | | gene | 52631963 | | 52636373 | LOC101102519 |
| 22 | 52625001 | 52675000 | 22 | Liftoff | | gene | 52643117 | | 52650956 | LOC101115913 |
| 22 | 52625001 | 52675000 | 22 | Liftoff | | gene | 52661326 | | 52682167 | LOC101102765 |
| 2 | 26825001 | 26875000 | 2 | Liftoff | | gene | 26652485 | | 26851286 | UBR3 |
| 2 | 26825001 | 26875000 | 2 | Liftoff | | gene | 26865242 | | 26874493 | METTL5 |
| 2 | 26825001 | 26875000 | 2 | Liftoff | | gene | 26873222 | | 26882432 | SSB |
| 2 | 26850001 | 26900000 | 2 | Liftoff | | gene | 26865242 | | 26874493 | METTL5 |
| 2 | 26850001 | 26900000 | 2 | Liftoff | | gene | 26873222 | | 26882432 | SSB |
| 2 | 26850001 | 26900000 | 2 | Liftoff | | gene | 26882501 | | 26884699 | LOC105607489 |
| 23 | 17325001 | 17375000 | 23 | Liftoff | | gene | 17346873 | | 17364960 | VEGFA |
| 23 | 17750001 | 17800000 | 23 | Liftoff | | gene | 17747205 | | 17759759 | CAPN11 |
| 23 | 17750001 | 17800000 | 23 | Liftoff | | gene | 17778907 | | 17782852 | MYMX |
| 23 | 17750001 | 17800000 | 23 | Liftoff | | gene | 17785103 | | 17794626 | SLC29A1 |
| 23 | 17775001 | 17825000 | 23 | Liftoff | | gene | 17778907 | | 17782852 | MYMX |
| 23 | 17775001 | 17825000 | 23 | Liftoff | | gene | 17785103 | | 17794626 | SLC29A1 |
| 23 | 17775001 | 17825000 | 23 | Liftoff | | gene | 17800902 | | 17804691 | LOC105603715 |
| 23 | 17775001 | 17825000 | 23 | Liftoff | | gene | 17805435 | | 17811134 | HSP90AB1 |
| 23 | 17775001 | 17825000 | 23 | Liftoff | | gene | 17812944 | | 17816339 | SLC35B2 |
| 23 | 17775001 | 17825000 | 23 | Liftoff | | gene | 17817254 | | 17824754 | NFKBIE |
| 23 | 18375001 | 18425000 | 23 | Liftoff | | gene | 18282494 | | 18679943 | SUPT3H |
| 23 | 18400001 | 18450000 | 23 | Liftoff | | gene | 18282494 | | 18679943 | SUPT3H |
| 23 | 50625001 | 50675000 | 23 | Liftoff | | gene | 50623709 | | 50643818 | BPHL |
| 23 | 50625001 | 50675000 | 23 | Liftoff | | gene | 50646518 | | 50674053 | RIPK1 |
| 2 | 35625001 | 35675000 | 2 | Liftoff | | gene | 35594148 | | 35692769 | TANK |
| 24 | 23550001 | 23600000 | 24 | Liftoff | | gene | 23404540 | | 23751684 | LOC101112868 |
| 24 | 24575001 | 24625000 | 24 | Liftoff | | gene | 24367665 | | 24746263 | CCDC178 |
| 24 | 25725001 | 25775000 | 24 | Liftoff | | gene | 25696585 | | 25740784 | RNF125 |
| 24 | 26325001 | 26375000 | 24 | Liftoff | | gene | 26333551 | | 26375058 | DSG1 |
| 24 | 30500001 | 30550000 | 24 | Liftoff | | gene | 30291619 | | 30527301 | CHST9 |
| 24 | 30500001 | 30550000 | 24 | Liftoff | | gene | 30514967 | | 30515796 | LOC105604441 |
| 24 | 30525001 | 30575000 | 24 | Liftoff | | gene | 30572210 | | 30582330 | AQP4 |
| 24 | 30550001 | 30600000 | 24 | Liftoff | | gene | 30572210 | | 30582330 | AQP4 |
| 24 | 30575001 | 30625000 | 24 | Liftoff | | gene | 30572210 | | 30582330 | AQP4 |
| 24 | 33375001 | 33425000 | 24 | Liftoff | | gene | 33410648 | | 33495338 | LOC114110447 |
| 24 | 33900001 | 33950000 | 24 | Liftoff | | gene | 33815076 | | 33930433 | TMEM241 |
| 2 | 4425001 | 4475000 | 2 | Liftoff | | gene | 4428864 | | 4537694 | UGGT1 |
| 24 | 43350001 | 43400000 | 24 | Liftoff | | gene | 43361426 | | 43408555 | GNAL |
| 24 | 43350001 | 43400000 | 24 | Liftoff | | gene | 43395593 | | 43398626 | CHMP1B |
| 24 | 43375001 | 43425000 | 24 | Liftoff | | gene | 43361426 | | 43408555 | GNAL |
| 24 | 43375001 | 43425000 | 24 | Liftoff | | gene | 43395593 | | 43398626 | CHMP1B |
| 24 | 43375001 | 43425000 | 24 | Liftoff | | gene | 43409258 | | 43423609 | MPPE1 |
| 24 | 46550001 | 46600000 | 24 | Liftoff | | gene | 46483780 | | 46565894 | C23H18orf25 |
| 24 | 59000001 | 59050000 | 24 | Liftoff | | gene | 58879168 | | 59109743 | CCBE1 |
| 24 | 61475001 | 61525000 | 24 | Liftoff | | gene | 61501631 | | 61506951 | LOC114110432 |
| 24 | 61500001 | 61550000 | 24 | Liftoff | | gene | 61501631 | | 61506951 | LOC114110432 |
| 24 | 61500001 | 61550000 | 24 | Liftoff | | gene | 61507528 | | 61739023 | PHLPP1 |
| 24 | 61750001 | 61800000 | 24 | Liftoff | | gene | 61799333 | | 61801732 | LOC105604596 |
| 24 | 61775001 | 61825000 | 24 | Liftoff | | gene | 61799333 | | 61801732 | LOC105604596 |
| 24 | 62300001 | 62350000 | 24 | Liftoff | | gene | 62304484 | | 62312169 | LOC101103612 |
| 24 | 62300001 | 62350000 | 24 | Liftoff | | gene | 62333660 | | 62340969 | LOC101103862 |
| 24 | 62325001 | 62375000 | 24 | Liftoff | | gene | 62333660 | | 62340969 | LOC101103862 |
| 24 | 62325001 | 62375000 | 24 | Liftoff | | gene | 62351365 | | 62381717 | LOC101104114 |
| 25 | 11050001 | 11100000 | 25 | Liftoff | | gene | 11076743 | | 11097690 | LOC105604659 |
| 25 | 11075001 | 11125000 | 25 | Liftoff | | gene | 11076743 | | 11097690 | LOC105604659 |
| 25 | 15600001 | 15650000 | 25 | Liftoff | | gene | 15574411 | | 15630926 | LOC101111247 |
| 25 | 1850001 | 1900000 | 25 | Liftoff | | gene | 1817916 | | 1869307 | LMF1 |
| 25 | 1850001 | 1900000 | 25 | Liftoff | | gene | 1880872 | | 1885672 | SOX8 |
| 25 | 18975001 | 19025000 | 25 | Liftoff | | gene | 18961470 | | 18980524 | GP2 |
| 25 | 18975001 | 19025000 | 25 | Liftoff | | gene | 18983832 | | 19000052 | UMOD |
| 25 | 18975001 | 19025000 | 25 | Liftoff | | gene | 19003928 | | 19051158 | PDILT |
| 25 | 19175001 | 19225000 | 25 | Liftoff | | gene | 19143908 | | 19189819 | LOC101120131 |
| 25 | 19175001 | 19225000 | 25 | Liftoff | | gene | 19214495 | | 19278541 | ACSM1 |
| 25 | 19475001 | 19525000 | 25 | Liftoff | | gene | 19502739 | | 19564129 | ACSM3 |
| 25 | 19500001 | 19550000 | 25 | Liftoff | | gene | 19502739 | | 19564129 | ACSM3 |
| 25 | 19525001 | 19575000 | 25 | Liftoff | | gene | 19502739 | | 19564129 | ACSM3 |
| 25 | 19525001 | 19575000 | 25 | Liftoff | | gene | 19563993 | | 19578663 | ERI2 |
| 25 | 19650001 | 19700000 | 25 | Liftoff | | gene | 19642133 | | 19698124 | DCUN1D3 |
| 25 | 19675001 | 19725000 | 25 | Liftoff | | gene | 19642133 | | 19698124 | DCUN1D3 |
| 25 | 19675001 | 19725000 | 25 | Liftoff | | gene | 19707117 | | 19732958 | LOC114110607 |
| 25 | 21550001 | 21600000 | 25 | Liftoff | | gene | 21546759 | | 21658055 | HS3ST2 |
| 25 | 22250001 | 22300000 | 25 | Liftoff | | gene | 22262134 | | 22290604 | EARS2 |
| 25 | 22250001 | 22300000 | 25 | Liftoff | | gene | 22290778 | | 22306077 | UBFD1 |
| 25 | 22400001 | 22450000 | 25 | Liftoff | | gene | 22396065 | | 22410693 | PLK1 |
| 25 | 22400001 | 22450000 | 25 | Liftoff | | gene | 22410627 | | 22434920 | ERN2 |
| 25 | 24525001 | 24575000 | 25 | Liftoff | | gene | 24340939 | | 24820503 | HS3ST4 |
| 25 | 3625001 | 3675000 | 25 | Liftoff | | gene | 3630821 | | 3636991 | ZNF213 |
| 25 | 3625001 | 3675000 | 25 | Liftoff | | gene | 3640001 | | 3644070 | LOC101120797 |
| 25 | 3625001 | 3675000 | 25 | Liftoff | | gene | 3643964 | | 3644036 | TRNAR-CCG-6 |
| 25 | 3625001 | 3675000 | 25 | Liftoff | | gene | 3646283 | | 3646355 | TRNAR-CCU-19 |
| 25 | 3625001 | 3675000 | 25 | Liftoff | | gene | 3652176 | | 3652248 | TRNAK-CUU-20 |
| 25 | 3625001 | 3675000 | 25 | Liftoff | | gene | 3653196 | | 3653267 | TRNAP-UGG-4 |
| 25 | 3625001 | 3675000 | 25 | Liftoff | | gene | 3656294 | | 3656365 | TRNAP-AGG-8 |
| 25 | 3625001 | 3675000 | 25 | Liftoff | | gene | 3656883 | | 3656955 | TRNAK-CUU-21 |
| 25 | 3625001 | 3675000 | 25 | Liftoff | | gene | 3660151 | | 3660222 | TRNAP-AGG-9 |
| 25 | 3625001 | 3675000 | 25 | Liftoff | | gene | 3663777 | | 3663849 | TRNAK-CUU-22 |
| 25 | 3625001 | 3675000 | 25 | Liftoff | | gene | 3670124 | | 3670196 | TRNAK-CUU-23 |
| 25 | 3625001 | 3675000 | 25 | Liftoff | | gene | 3671388 | | 3671459 | TRNAP-CGG-4 |
| 25 | 3625001 | 3675000 | 25 | Liftoff | | gene | 3673479 | | 3673551 | TRNAR-CCU-20 |
| 25 | 3625001 | 3675000 | 25 | Liftoff | | gene | 3674737 | | 3674809 | TRNAK-CUU-24 |
| 25 | 39600001 | 39650000 | 25 | Liftoff | | gene | 39520859 | | 39641798 | RNF216 |
| 25 | 42025001 | 42075000 | 25 | Liftoff | | gene | 41845697 | | 42091568 | MAD1L1 |
| 25 | 42175001 | 42225000 | 25 | Liftoff | | gene | 42160079 | | 42185617 | LOC114110603 |
| 25 | 42200001 | 42250000 | 25 | Liftoff | | gene | 42247853 | | 42249526 | PSMG3 |
| 25 | 8775001 | 8825000 | 25 | Liftoff | | gene | 8767639 | | 8822615 | USP7 |
| 25 | 8800001 | 8850000 | 25 | Liftoff | | gene | 8767639 | | 8822615 | USP7 |
| 26 | 13625001 | 13675000 | 26 | Liftoff | | gene | 13597192 | | 13651599 | 5-Mar |
| 26 | 30225001 | 30275000 | 26 | Liftoff | | gene | 30159970 | | 30289770 | ADD3 |
| 26 | 34275001 | 34325000 | 26 | Liftoff | | gene | 34231261 | | 34292852 | TDRD1 |
| 26 | 34275001 | 34325000 | 26 | Liftoff | | gene | 34303214 | | 34351407 | VWA2 |
| 26 | 43025001 | 43075000 | 26 | Liftoff | | gene | 43013611 | | 43150811 | CPXM2 |
| 26 | 50675001 | 50725000 | 26 | Liftoff | | gene | 50616691 | | 50767870 | INPP5A |
| 26 | 50700001 | 50750000 | 26 | Liftoff | | gene | 50616691 | | 50767870 | INPP5A |
| 2 | 6525001 | 6575000 | 2 | Liftoff | | gene | 6512853 | | 6590720 | LOC114113051 |
| 2 | 6600001 | 6650000 | 2 | Liftoff | | gene | 6601670 | | 6712314 | PMS1 |
| 2 | 6625001 | 6675000 | 2 | Liftoff | | gene | 6601670 | | 6712314 | PMS1 |
| 2 | 6700001 | 6750000 | 2 | Liftoff | | gene | 6601670 | | 6712314 | PMS1 |
| 2 | 6700001 | 6750000 | 2 | Liftoff | | gene | 6712437 | | 6723812 | ORMDL1 |
| 2 | 6700001 | 6750000 | 2 | Liftoff | | gene | 6726654 | | 6728551 | LOC114113053 |
| 2 | 6700001 | 6750000 | 2 | Liftoff | | gene | 6729355 | | 6731189 | LOC114113052 |
| 2 | 6700001 | 6750000 | 2 | Liftoff | | gene | 6731267 | | 6743312 | OSGEPL1 |
| 2 | 6700001 | 6750000 | 2 | Liftoff | | gene | 6744302 | | 6796969 | ANKAR |
| 27 | 32775001 | 32825000 | 27 | Liftoff | | gene | 32751928 | | 32855531 | NSD3 |
| 27 | 37200001 | 37250000 | 27 | Liftoff | | gene | 37179539 | | 37211885 | HGSNAT |
| 27 | 37200001 | 37250000 | 27 | Liftoff | | gene | 37218697 | | 37249921 | INTS10 |
| 27 | 37225001 | 37275000 | 27 | Liftoff | | gene | 37218697 | | 37249921 | INTS10 |
| 28 | 12225001 | 12275000 | 28 | Liftoff | | gene | 12246462 | | 12271687 | LOC101119014 |
| 2 | 81250001 | 81300000 | 2 | Liftoff | | gene | 81210204 | | 81495624 | TMEFF2 |
| 28 | 125001 | 175000 | 28 | Liftoff | | gene | 166513 | | 176102 | RHOU |
| 28 | 12700001 | 12750000 | 28 | Liftoff | | gene | 12714942 | | 12776597 | CSGALNACT2 |
| 28 | 12725001 | 12775000 | 28 | Liftoff | | gene | 12714942 | | 12776597 | CSGALNACT2 |
| 28 | 12750001 | 12800000 | 28 | Liftoff | | gene | 12714942 | | 12776597 | CSGALNACT2 |
| 28 | 12750001 | 12800000 | 28 | Liftoff | | gene | 12772897 | | 12813433 | RASGEF1A |
| 28 | 15050001 | 15100000 | 28 | Liftoff | | gene | 14873647 | | 15251504 | ANK3 |
| 28 | 15050001 | 15100000 | 28 | Liftoff | | gene | 15078462 | | 15078778 | LOC101120721 |
| 28 | 15075001 | 15125000 | 28 | Liftoff | | gene | 14873647 | | 15251504 | ANK3 |
| 28 | 15075001 | 15125000 | 28 | Liftoff | | gene | 15078462 | | 15078778 | LOC101120721 |
| 28 | 16700001 | 16750000 | 28 | Liftoff | | gene | 16711652 | | 16882243 | CABCOCO1 |
| 28 | 2225001 | 2275000 | 28 | Liftoff | | gene | 2225023 | | 2225141 | LOC114110900 |
| 28 | 2225001 | 2275000 | 28 | Liftoff | | gene | 2227067 | | 2227185 | LOC114110873 |
| 28 | 2225001 | 2275000 | 28 | Liftoff | | gene | 2272128 | | 2272246 | LOC114113267 |
| 28 | 23925001 | 23975000 | 28 | Liftoff | | gene | 23920876 | | 23949699 | SLC25A16 |
| 28 | 23950001 | 24000000 | 28 | Liftoff | | gene | 23975373 | | 24115681 | TET1 |
| 28 | 34800001 | 34850000 | 28 | Liftoff | | gene | 34796886 | | 34873569 | SH2D4B |
| 28 | 34825001 | 34875000 | 28 | Liftoff | | gene | 34796886 | | 34873569 | SH2D4B |
| 28 | 4050001 | 4100000 | 28 | Liftoff | | gene | 3806089 | | 4250865 | DISC1 |
| 28 | 4075001 | 4125000 | 28 | Liftoff | | gene | 3806089 | | 4250865 | DISC1 |
| 28 | 41750001 | 41800000 | 28 | Liftoff | | gene | 41627914 | | 41810755 | ARHGAP22 |
| 28 | 41775001 | 41825000 | 28 | Liftoff | | gene | 41627914 | | 41810755 | ARHGAP22 |
| 28 | 7100001 | 7150000 | 28 | Liftoff | | gene | 7104963 | | 7114216 | LOC105604916 |
| 28 | 8050001 | 8100000 | 28 | Liftoff | | gene | 8030749 | | 8130056 | NID1 |
| 28 | 8075001 | 8125000 | 28 | Liftoff | | gene | 8030749 | | 8130056 | NID1 |
| 29 | 11975001 | 12025000 | 29 | Liftoff | | gene | 11963988 | | 11987888 | PCF11 |
| 29 | 12000001 | 12050000 | 29 | Liftoff | | gene | 12046038 | | 12046705 | LOC101106977 |
| 29 | 17725001 | 17775000 | 29 | Liftoff | | gene | 17647933 | | 17800022 | RSF1 |
| 29 | 2000001 | 2050000 | 29 | Liftoff | | gene | 1936392 | | 2159008 | LOC114110152 |
| 29 | 21800001 | 21850000 | 29 | Liftoff | | gene | 21841392 | | 21882355 | SLC17A6 |
| 29 | 49850001 | 49900000 | 29 | Liftoff | | gene | 49818824 | | 49855752 | LSP1 |
| 29 | 49850001 | 49900000 | 29 | Liftoff | | gene | 49864499 | | 49867041 | TNNI2 |
| 29 | 49850001 | 49900000 | 29 | Liftoff | | gene | 49867758 | | 49870821 | SYT8 |
| 29 | 50975001 | 51025000 | 29 | Liftoff | | gene | 50978719 | | 50993920 | RNH1 |
| 29 | 50975001 | 51025000 | 29 | Liftoff | | gene | 50995042 | | 51020274 | PTDSS2 |
| 29 | 51000001 | 51050000 | 29 | Liftoff | | gene | 50995042 | | 51020274 | PTDSS2 |
| 29 | 51000001 | 51050000 | 29 | Liftoff | | gene | 51035452 | | 51057690 | ANO9 |
| 29 | 51025001 | 51075000 | 29 | Liftoff | | gene | 51035452 | | 51057690 | ANO9 |
| 29 | 51025001 | 51075000 | 29 | Liftoff | | gene | 51061425 | | 51067459 | SIGIRR |
| 29 | 51025001 | 51075000 | 29 | Liftoff | | gene | 51068469 | | 51076296 | PKP3 |
| 29 | 51050001 | 51100000 | 29 | Liftoff | | gene | 51035452 | | 51057690 | ANO9 |
| 29 | 51050001 | 51100000 | 29 | Liftoff | | gene | 51061425 | | 51067459 | SIGIRR |
| 29 | 51050001 | 51100000 | 29 | Liftoff | | gene | 51068469 | | 51076296 | PKP3 |
| 29 | 51050001 | 51100000 | 29 | Liftoff | | gene | 51087775 | | 51100714 | B4GALNT4 |
| 29 | 9125001 | 9175000 | 29 | Liftoff | | gene | 9084960 | | 9138901 | CCDC83 |
| 3 | 11500001 | 11550000 | 3 | Liftoff | | gene | 11529971 | | 11530924 | LOC101116954 |
| 3 | 11525001 | 11575000 | 3 | Liftoff | | gene | 11529971 | | 11530924 | LOC101116954 |
| 3 | 11525001 | 11575000 | 3 | Liftoff | | gene | 11550777 | | 11551718 | LOC101111923 |
| 3 | 11525001 | 11575000 | 3 | Liftoff | | gene | 11572780 | | 11573718 | LOC101116699 |
| 3 | 120800001 | 120850000 | 3 | Liftoff | | gene | 120798268 | | 120806863 | RNPEPL1 |
| 3 | 120800001 | 120850000 | 3 | Liftoff | | gene | 120808502 | | 120812942 | LOC114113946 |
| 3 | 120800001 | 120850000 | 3 | Liftoff | | gene | 120813107 | | 120823908 | CAPN10 |
| 3 | 120800001 | 120850000 | 3 | Liftoff | | gene | 120841730 | | 120849776 | GPR35 |
| 3 | 120825001 | 120875000 | 3 | Liftoff | | gene | 120841730 | | 120849776 | GPR35 |
| 3 | 13200001 | 13250000 | 3 | Liftoff | | gene | 13196521 | | 13213053 | LOC101111069 |
| 3 | 21200001 | 21250000 | 3 | Liftoff | | gene | 21198391 | | 21205260 | LOC105612463 |
| 3 | 21200001 | 21250000 | 3 | Liftoff | | gene | 21214698 | | 21214767 | TRNAQ-CUG-7 |
| 3 | 21200001 | 21250000 | 3 | Liftoff | | gene | 21217062 | | 21217133 | TRNAN-GUU-4 |
| 3 | 21200001 | 21250000 | 3 | Liftoff | | gene | 21217715 | | 21217784 | TRNAV-CAC-3 |
| 3 | 21200001 | 21250000 | 3 | Liftoff | | gene | 21226897 | | 21226969 | TRNAV-CAC-6 |
| 3 | 21200001 | 21250000 | 3 | Liftoff | | gene | 21230634 | | 21230707 | TRNAN-GUU-24 |
| 3 | 21200001 | 21250000 | 3 | Liftoff | | gene | 21231312 | | 21231384 | TRNAV-CAC-2 |
| 3 | 21225001 | 21275000 | 3 | Liftoff | | gene | 21226897 | | 21226969 | TRNAV-CAC-6 |
| 3 | 21225001 | 21275000 | 3 | Liftoff | | gene | 21230634 | | 21230707 | TRNAN-GUU-24 |
| 3 | 21225001 | 21275000 | 3 | Liftoff | | gene | 21231312 | | 21231384 | TRNAV-CAC-2 |
| 3 | 21225001 | 21275000 | 3 | Liftoff | | gene | 21253537 | | 21253608 | TRNAQ-CUG-10 |
| 3 | 21225001 | 21275000 | 3 | Liftoff | | gene | 21255972 | | 21256045 | TRNAN-GUU-20 |
| 3 | 21225001 | 21275000 | 3 | Liftoff | | gene | 21256650 | | 21256722 | TRNAV-CAC-11 |
| 3 | 21225001 | 21275000 | 3 | Liftoff | | gene | 21257220 | | 21257291 | TRNASTOP-UCA |
| 3 | 21225001 | 21275000 | 3 | Liftoff | | gene | 21258055 | | 21258125 | TRNAG-CCC-15 |
| 3 | 21225001 | 21275000 | 3 | Liftoff | | gene | 21265958 | | 21266030 | TRNAV-AAC-3 |
| 3 | 21225001 | 21275000 | 3 | Liftoff | | gene | 21272275 | | 21272347 | TRNAV-AAC-5 |
| 3 | 21275001 | 21325000 | 3 | Liftoff | | gene | 21278854 | | 21278925 | TRNAH-GUG-9 |
| 3 | 21275001 | 21325000 | 3 | Liftoff | | gene | 21286177 | | 21286247 | TRNAG-CCC-16 |
| 3 | 21275001 | 21325000 | 3 | Liftoff | | gene | 21287008 | | 21287079 | TRNAE-UUC-17 |
| 3 | 21275001 | 21325000 | 3 | Liftoff | | gene | 21287576 | | 21287648 | TRNAV-CAC-10 |
| 3 | 21275001 | 21325000 | 3 | Liftoff | | gene | 21288253 | | 21288326 | TRNAN-GUU-25 |
| 3 | 21275001 | 21325000 | 3 | Liftoff | | gene | 21295987 | | 21296058 | TRNAQ-CUG-11 |
| 3 | 21275001 | 21325000 | 3 | Liftoff | | gene | 21309001 | | 21309164 | LOC114111403 |
| 3 | 21275001 | 21325000 | 3 | Liftoff | | gene | 21316624 | | 21316695 | TRNAQ-CUG-12 |
| 3 | 21275001 | 21325000 | 3 | Liftoff | | gene | 21324369 | | 21324442 | TRNAN-GUU-22 |
| 3 | 21300001 | 21350000 | 3 | Liftoff | | gene | 21309001 | | 21309164 | LOC114111403 |
| 3 | 21300001 | 21350000 | 3 | Liftoff | | gene | 21316624 | | 21316695 | TRNAQ-CUG-12 |
| 3 | 21300001 | 21350000 | 3 | Liftoff | | gene | 21324369 | | 21324442 | TRNAN-GUU-22 |
| 3 | 21300001 | 21350000 | 3 | Liftoff | | gene | 21325047 | | 21325119 | TRNAV-CAC-12 |
| 3 | 21300001 | 21350000 | 3 | Liftoff | | gene | 21325617 | | 21325688 | TRNAE-UUC-14 |
| 3 | 21300001 | 21350000 | 3 | Liftoff | | gene | 21326452 | | 21326522 | TRNAG-CCC-14 |
| 3 | 21300001 | 21350000 | 3 | Liftoff | | gene | 21333773 | | 21333844 | TRNAH-GUG-7 |
| 3 | 21300001 | 21350000 | 3 | Liftoff | | gene | 21340352 | | 21340424 | TRNAV-AAC-6 |
| 3 | 21300001 | 21350000 | 3 | Liftoff | | gene | 21346673 | | 21346745 | TRNAV-AAC-7 |
| 3 | 21325001 | 21375000 | 3 | Liftoff | | gene | 21325047 | | 21325119 | TRNAV-CAC-12 |
| 3 | 21325001 | 21375000 | 3 | Liftoff | | gene | 21325617 | | 21325688 | TRNAE-UUC-14 |
| 3 | 21325001 | 21375000 | 3 | Liftoff | | gene | 21326452 | | 21326522 | TRNAG-CCC-14 |
| 3 | 21325001 | 21375000 | 3 | Liftoff | | gene | 21333773 | | 21333844 | TRNAH-GUG-7 |
| 3 | 21325001 | 21375000 | 3 | Liftoff | | gene | 21340352 | | 21340424 | TRNAV-AAC-6 |
| 3 | 21325001 | 21375000 | 3 | Liftoff | | gene | 21346673 | | 21346745 | TRNAV-AAC-7 |
| 3 | 21325001 | 21375000 | 3 | Liftoff | | gene | 21354675 | | 21354745 | TRNAG-CCC-13 |
| 3 | 21325001 | 21375000 | 3 | Liftoff | | gene | 21355509 | | 21355580 | TRNAE-UUC-15 |
| 3 | 21325001 | 21375000 | 3 | Liftoff | | gene | 21356078 | | 21356150 | TRNAV-CAC-13 |
| 3 | 21325001 | 21375000 | 3 | Liftoff | | gene | 21356754 | | 21356827 | TRNAN-GUU-23 |
| 3 | 21325001 | 21375000 | 3 | Liftoff | | gene | 21359183 | | 21359254 | TRNAQ-CUG-13 |
| 3 | 26625001 | 26675000 | 3 | Liftoff | | gene | 26597484 | | 26648127 | TTF2 |
| 3 | 28700001 | 28750000 | 3 | Liftoff | | gene | 28679256 | | 28719030 | TSPAN2 |
| 3 | 28700001 | 28750000 | 3 | Liftoff | | gene | 28731940 | | 28737122 | TSHB |
| 3 | 28725001 | 28775000 | 3 | Liftoff | | gene | 28731940 | | 28737122 | TSHB |
| 3 | 28725001 | 28775000 | 3 | Liftoff | | gene | 28758353 | | 28892520 | SYCP1 |
| 3 | 28925001 | 28975000 | 3 | Liftoff | | gene | 28961394 | | 28967104 | SIKE1 |
| 3 | 28950001 | 29000000 | 3 | Liftoff | | gene | 28961394 | | 28967104 | SIKE1 |
| 3 | 28950001 | 29000000 | 3 | Liftoff | | gene | 28983985 | | 29019251 | CSDE1 |
| 3 | 28975001 | 29025000 | 3 | Liftoff | | gene | 28983985 | | 29019251 | CSDE1 |
| 3 | 28975001 | 29025000 | 3 | Liftoff | | gene | 29019373 | | 29029607 | NRAS |
| 3 | 29000001 | 29050000 | 3 | Liftoff | | gene | 28983985 | | 29019251 | CSDE1 |
| 3 | 29000001 | 29050000 | 3 | Liftoff | | gene | 29019373 | | 29029607 | NRAS |
| 3 | 29000001 | 29050000 | 3 | Liftoff | | gene | 29040770 | | 29063435 | AMPD1 |
| 3 | 29025001 | 29075000 | 3 | Liftoff | | gene | 29019373 | | 29029607 | NRAS |
| 3 | 29025001 | 29075000 | 3 | Liftoff | | gene | 29040770 | | 29063435 | AMPD1 |
| 3 | 29050001 | 29100000 | 3 | Liftoff | | gene | 29040770 | | 29063435 | AMPD1 |
| 3 | 29075001 | 29125000 | 3 | Liftoff | | gene | 29110561 | | 29155904 | DENND2C |
| 3 | 29350001 | 29400000 | 3 | Liftoff | | gene | 29236687 | | 29382898 | TRIM33 |
| 3 | 35450001 | 35500000 | 3 | Liftoff | | gene | 35446930 | | 35505496 | SLC25A24 |
| 3 | 35875001 | 35925000 | 3 | Liftoff | | gene | 35734749 | | 36163862 | VAV3 |
| 3 | 50325001 | 50375000 | 3 | Liftoff | | gene | 50343701 | | 50453695 | FNBP1L |
| 3 | 50350001 | 50400000 | 3 | Liftoff | | gene | 50343701 | | 50453695 | FNBP1L |
| 3 | 50375001 | 50425000 | 3 | Liftoff | | gene | 50343701 | | 50453695 | FNBP1L |
| 3 | 50400001 | 50450000 | 3 | Liftoff | | gene | 50343701 | | 50453695 | FNBP1L |
| 3 | 50425001 | 50475000 | 3 | Liftoff | | gene | 50343701 | | 50453695 | FNBP1L |
| 3 | 52200001 | 52250000 | 3 | Liftoff | | gene | 52228189 | | 52254408 | CDC7 |
| 3 | 52225001 | 52275000 | 3 | Liftoff | | gene | 52228189 | | 52254408 | CDC7 |
| 3 | 59550001 | 59600000 | 3 | Liftoff | | gene | 59550314 | | 59593215 | SYDE2 |
| 3 | 59550001 | 59600000 | 3 | Liftoff | | gene | 59596062 | | 59613271 | LOC114115574 |
| 3 | 59575001 | 59625000 | 3 | Liftoff | | gene | 59550314 | | 59593215 | SYDE2 |
| 3 | 59575001 | 59625000 | 3 | Liftoff | | gene | 59596062 | | 59613271 | LOC114115574 |
| 3 | 81450001 | 81500000 | 3 | Liftoff | | gene | 81354335 | | 81488037 | JAK1 |
| 3 | 82125001 | 82175000 | 3 | Liftoff | | gene | 82162202 | | 82240521 | UBE2U |
| 3 | 87725001 | 87775000 | 3 | Liftoff | | gene | 87765406 | | 87803079 | LOC105608331 |
| 3 | 87750001 | 87800000 | 3 | Liftoff | | gene | 87765406 | | 87803079 | LOC105608331 |
| 3 | 88550001 | 88600000 | 3 | Liftoff | | gene | 88530372 | | 88593160 | OMA1 |
| 3 | 88575001 | 88625000 | 3 | Liftoff | | gene | 88530372 | | 88593160 | OMA1 |
| 3 | 90375001 | 90425000 | 3 | Liftoff | | gene | 90381742 | | 90532717 | FYB2 |
| 4 | 103900001 | 103950000 | 4 | Liftoff | | gene | 103892890 | | 103901195 | LOC105609822 |
| 4 | 103900001 | 103950000 | 4 | Liftoff | | gene | 103902396 | | 103916303 | KLRG2 |
| 4 | 103900001 | 103950000 | 4 | Liftoff | | gene | 103945836 | | 103967816 | CLEC2L |
| 4 | 106200001 | 106250000 | 4 | Liftoff | | gene | 106159611 | | 106242993 | MGAM |
| 4 | 106875001 | 106925000 | 4 | Liftoff | | gene | 106908335 | | 106915575 | EPHB6 |
| 4 | 106875001 | 106925000 | 4 | Liftoff | | gene | 106915662 | | 106932027 | TRPV6 |
| 4 | 113700001 | 113750000 | 4 | Liftoff | | gene | 113740492 | | 113742338 | LOC101114852 |
| 4 | 116400001 | 116450000 | 4 | Liftoff | | gene | 116447219 | | 116471369 | XRCC2 |
| 4 | 116425001 | 116475000 | 4 | Liftoff | | gene | 116447219 | | 116471369 | XRCC2 |
| 4 | 118550001 | 118600000 | 4 | Liftoff | | gene | 118555972 | | 118566561 | LOC114114628 |
| 4 | 34350001 | 34400000 | 4 | Liftoff | | gene | 34324461 | | 34538156 | KIAA1324L |
| 4 | 44500001 | 44550000 | 4 | Liftoff | | gene | 44441171 | | 44577434 | PHTF2 |
| 4 | 44525001 | 44575000 | 4 | Liftoff | | gene | 44441171 | | 44577434 | PHTF2 |
| 4 | 72700001 | 72750000 | 4 | Liftoff | | gene | 72735874 | | 72742681 | NPY |
| 4 | 72725001 | 72775000 | 4 | Liftoff | | gene | 72735874 | | 72742681 | NPY |
| 4 | 800001 | 850000 | 4 | Liftoff | | gene | 841827 | | 847659 | LOC114114034 |
| 4 | 94975001 | 95025000 | 4 | Liftoff | | gene | 95012063 | | 95027925 | ZC3HC1 |
| 5 | 15575001 | 15625000 | 5 | Liftoff | | gene | 15568591 | | 15583408 | MGAT4C |
| 5 | 18025001 | 18075000 | 5 | Liftoff | | gene | 18047410 | | 18177663 | KITLG |
| 5 | 18050001 | 18100000 | 5 | Liftoff | | gene | 18047410 | | 18177663 | KITLG |
| 5 | 24550001 | 24600000 | 5 | Liftoff | | gene | 24551096 | | 24658773 | FGD6 |
| 5 | 24700001 | 24750000 | 5 | Liftoff | | gene | 24659162 | | 24745231 | VEZT |
| 5 | 24725001 | 24775000 | 5 | Liftoff | | gene | 24659162 | | 24745231 | VEZT |
| 5 | 24850001 | 24900000 | 5 | Liftoff | | gene | 24887726 | | 24916994 | METAP2 |
| 5 | 24875001 | 24925000 | 5 | Liftoff | | gene | 24887726 | | 24916994 | METAP2 |
| 5 | 24875001 | 24925000 | 5 | Liftoff | | gene | 24916740 | | 24931494 | USP44 |
| 5 | 24900001 | 24950000 | 5 | Liftoff | | gene | 24887726 | | 24916994 | METAP2 |
| 5 | 24900001 | 24950000 | 5 | Liftoff | | gene | 24916740 | | 24931494 | USP44 |
| 5 | 25200001 | 25250000 | 5 | Liftoff | | gene | 25230505 | | 25233231 | GLYCAM1 |
| 5 | 25225001 | 25275000 | 5 | Liftoff | | gene | 25230505 | | 25233231 | GLYCAM1 |
| 5 | 25225001 | 25275000 | 5 | Liftoff | | gene | 25258910 | | 25266938 | PPP1R1A |
| 5 | 25225001 | 25275000 | 5 | Liftoff | | gene | 25267770 | | 25294469 | PDE1B |
| 5 | 25275001 | 25325000 | 5 | Liftoff | | gene | 25267770 | | 25294469 | PDE1B |
| 5 | 25275001 | 25325000 | 5 | Liftoff | | gene | 25299471 | | 25343691 | NCKAP1L |
| 5 | 25300001 | 25350000 | 5 | Liftoff | | gene | 25299471 | | 25343691 | NCKAP1L |
| 5 | 25450001 | 25500000 | 5 | Liftoff | | gene | 25440466 | | 25461524 | ZNF385A |
| 5 | 25450001 | 25500000 | 5 | Liftoff | | gene | 25463244 | | 25465207 | GPR84 |
| 5 | 25450001 | 25500000 | 5 | Liftoff | | gene | 25475823 | | 25495717 | COPZ1 |
| 5 | 25450001 | 25500000 | 5 | Liftoff | | gene | 25479547 | | 25481126 | LOC114113969 |
| 5 | 25475001 | 25525000 | 5 | Liftoff | | gene | 25475823 | | 25495717 | COPZ1 |
| 5 | 25475001 | 25525000 | 5 | Liftoff | | gene | 25479547 | | 25481126 | LOC114113969 |
| 5 | 25475001 | 25525000 | 5 | Liftoff | | gene | 25522129 | | 25529365 | NFE2 |
| 5 | 25875001 | 25925000 | 5 | Liftoff | | gene | 25923110 | | 25923211 | LOC114114198 |
| 5 | 26150001 | 26200000 | 5 | Liftoff | | gene | 26173268 | | 26270260 | ATF7 |
| 5 | 26175001 | 26225000 | 5 | Liftoff | | gene | 26173268 | | 26270260 | ATF7 |
| 5 | 26200001 | 26250000 | 5 | Liftoff | | gene | 26173268 | | 26270260 | ATF7 |
| 5 | 26375001 | 26425000 | 5 | Liftoff | | gene | 26357849 | | 26396553 | SP1 |
| 5 | 26400001 | 26450000 | 5 | Liftoff | | gene | 26436422 | | 26444746 | SP7 |
| 5 | 26425001 | 26475000 | 5 | Liftoff | | gene | 26436422 | | 26444746 | SP7 |
| 5 | 26425001 | 26475000 | 5 | Liftoff | | gene | 26448991 | | 26463051 | AAAS |
| 5 | 26425001 | 26475000 | 5 | Liftoff | | gene | 26463245 | | 26470174 | C3H12orf10 |
| 5 | 26425001 | 26475000 | 5 | Liftoff | | gene | 26470768 | | 26474270 | PFDN5 |
| 5 | 26450001 | 26500000 | 5 | Liftoff | | gene | 26448991 | | 26463051 | AAAS |
| 5 | 26450001 | 26500000 | 5 | Liftoff | | gene | 26463245 | | 26470174 | C3H12orf10 |
| 5 | 26450001 | 26500000 | 5 | Liftoff | | gene | 26470768 | | 26474270 | PFDN5 |
| 5 | 26450001 | 26500000 | 5 | Liftoff | | gene | 26475723 | | 26497410 | ESPL1 |
| 5 | 26475001 | 26525000 | 5 | Liftoff | | gene | 26475723 | | 26497410 | ESPL1 |
| 5 | 26475001 | 26525000 | 5 | Liftoff | | gene | 26503693 | | 26506037 | MFSD5 |
| 5 | 26475001 | 26525000 | 5 | Liftoff | | gene | 26508819 | | 26519924 | LOC105614722 |
| 5 | 26475001 | 26525000 | 5 | Liftoff | | gene | 26519448 | | 26540804 | RARG |
| 5 | 26500001 | 26550000 | 5 | Liftoff | | gene | 26503693 | | 26506037 | MFSD5 |
| 5 | 26500001 | 26550000 | 5 | Liftoff | | gene | 26508819 | | 26519924 | LOC105614722 |
| 5 | 26500001 | 26550000 | 5 | Liftoff | | gene | 26519448 | | 26540804 | RARG |
| 5 | 26500001 | 26550000 | 5 | Liftoff | | gene | 26540900 | | 26557463 | ITGB7 |
| 5 | 26550001 | 26600000 | 5 | Liftoff | | gene | 26540900 | | 26557463 | ITGB7 |
| 5 | 26550001 | 26600000 | 5 | Liftoff | | gene | 26557930 | | 26567862 | ZNF740 |
| 5 | 26550001 | 26600000 | 5 | Liftoff | | gene | 26567273 | | 26592349 | CSAD |
| 5 | 26575001 | 26625000 | 5 | Liftoff | | gene | 26567273 | | 26592349 | CSAD |
| 5 | 26575001 | 26625000 | 5 | Liftoff | | gene | 26611130 | | 26621179 | SOAT2 |
| 5 | 26575001 | 26625000 | 5 | Liftoff | | gene | 26622543 | | 26626082 | IGFBP6 |
| 5 | 26600001 | 26650000 | 5 | Liftoff | | gene | 26611130 | | 26621179 | SOAT2 |
| 5 | 26600001 | 26650000 | 5 | Liftoff | | gene | 26622543 | | 26626082 | IGFBP6 |
| 5 | 26600001 | 26650000 | 5 | Liftoff | | gene | 26632492 | | 26646555 | SPRYD3 |
| 5 | 26600001 | 26650000 | 5 | Liftoff | | gene | 26646576 | | 26662364 | TNS2 |
| 5 | 26625001 | 26675000 | 5 | Liftoff | | gene | 26622543 | | 26626082 | IGFBP6 |
| 5 | 26625001 | 26675000 | 5 | Liftoff | | gene | 26632492 | | 26646555 | SPRYD3 |
| 5 | 26625001 | 26675000 | 5 | Liftoff | | gene | 26646576 | | 26662364 | TNS2 |
| 5 | 26625001 | 26675000 | 5 | Liftoff | | gene | 26666778 | | 26692660 | EIF4B |
| 5 | 26650001 | 26700000 | 5 | Liftoff | | gene | 26646576 | | 26662364 | TNS2 |
| 5 | 26650001 | 26700000 | 5 | Liftoff | | gene | 26666778 | | 26692660 | EIF4B |
| 5 | 29875001 | 29925000 | 5 | Liftoff | | gene | 29833605 | | 29885931 | FMNL3 |
| 5 | 29875001 | 29925000 | 5 | Liftoff | | gene | 29886436 | | 29906879 | PRPF40B |
| 5 | 29875001 | 29925000 | 5 | Liftoff | | gene | 29907819 | | 29931884 | FAM186B |
| 5 | 32975001 | 33025000 | 5 | Liftoff | | gene | 32982080 | | 33016810 | SLC38A4 |
| 5 | 33000001 | 33050000 | 5 | Liftoff | | gene | 32982080 | | 33016810 | SLC38A4 |
| 5 | 37675001 | 37725000 | 5 | Liftoff | | gene | 37685592 | | 37853513 | PPHLN1 |
| 5 | 37700001 | 37750000 | 5 | Liftoff | | gene | 37685592 | | 37853513 | PPHLN1 |
| 5 | 37725001 | 37775000 | 5 | Liftoff | | gene | 37685592 | | 37853513 | PPHLN1 |
| 5 | 37750001 | 37800000 | 5 | Liftoff | | gene | 37685592 | | 37853513 | PPHLN1 |
| 5 | 37825001 | 37875000 | 5 | Liftoff | | gene | 37685592 | | 37853513 | PPHLN1 |
| 5 | 37825001 | 37875000 | 5 | Liftoff | | gene | 37853547 | | 37871135 | ZCRB1 |
| 5 | 46800001 | 46850000 | 5 | Liftoff | | gene | 46782141 | | 46819041 | HELB |
| 5 | 46825001 | 46875000 | 5 | Liftoff | | gene | 46866298 | | 46922509 | IRAK3 |
| 5 | 59525001 | 59575000 | 5 | Liftoff | | gene | 59543606 | | 59543677 | TRNAG-ACC-2 |
| 5 | 63475001 | 63525000 | 5 | Liftoff | | gene | 63434059 | | 63789400 | ANO4 |
| 5 | 64025001 | 64075000 | 5 | Liftoff | | gene | 64032386 | | 64042921 | ARL1 |
| 5 | 68825001 | 68875000 | 5 | Liftoff | | gene | 68678199 | | 68850189 | RFX4 |
| 5 | 68825001 | 68875000 | 5 | Liftoff | | gene | 68828043 | | 68833418 | LOC114114025 |
| 5 | 68825001 | 68875000 | 5 | Liftoff | | gene | 68861918 | | 68966275 | RIC8B |
| 5 | 68875001 | 68925000 | 5 | Liftoff | | gene | 68861918 | | 68966275 | RIC8B |
| 5 | 68900001 | 68950000 | 5 | Liftoff | | gene | 68861918 | | 68966275 | RIC8B |
| 5 | 68925001 | 68975000 | 5 | Liftoff | | gene | 68861918 | | 68966275 | RIC8B |
| 5 | 69150001 | 69200000 | 5 | Liftoff | | gene | 69073790 | | 69164782 | CRY1 |
| 5 | 69650001 | 69700000 | 5 | Liftoff | | gene | 69377789 | | 69698091 | BTBD11 |
| 5 | 69775001 | 69825000 | 5 | Liftoff | | gene | 69759546 | | 69783532 | PRDM4 |
| 5 | 69775001 | 69825000 | 5 | Liftoff | | gene | 69798786 | | 69799319 | ASCL4 |
| 5 | 69775001 | 69825000 | 5 | Liftoff | | gene | 69809657 | | 69831856 | RTCB |
| 5 | 69800001 | 69850000 | 5 | Liftoff | | gene | 69809657 | | 69831856 | RTCB |
| 5 | 69800001 | 69850000 | 5 | Liftoff | | gene | 69834449 | | 69872054 | BPIFC |
| 5 | 70150001 | 70200000 | 5 | Liftoff | | gene | 70184668 | | 70239515 | TIMP3 |
| 5 | 70175001 | 70225000 | 5 | Liftoff | | gene | 70184668 | | 70239515 | TIMP3 |
| 5 | 70200001 | 70250000 | 5 | Liftoff | | gene | 70184668 | | 70239515 | TIMP3 |
| 5 | 70275001 | 70325000 | 5 | Liftoff | | gene | 70244080 | | 70391426 | LOC101118937 |
| 5 | 72400001 | 72450000 | 5 | Liftoff | | gene | 72399050 | | 72407581 | HMOX1 |
| 5 | 72400001 | 72450000 | 5 | Liftoff | | gene | 72409951 | | 72428074 | MCM5 |
| 5 | 72600001 | 72650000 | 5 | Liftoff | | gene | 72586629 | | 72602993 | MB |
| 5 | 72600001 | 72650000 | 5 | Liftoff | | gene | 72644480 | | 72653315 | LOC101104568 |
| 5 | 76950001 | 77000000 | 5 | Liftoff | | gene | 76952407 | | 76963013 | ETFBKMT |
| 5 | 76975001 | 77025000 | 5 | Liftoff | | gene | 77018371 | | 77018476 | LOC114114180 |
| 5 | 77525001 | 77575000 | 5 | Liftoff | | gene | 77507910 | | 77576435 | IPO8 |
| 5 | 77550001 | 77600000 | 5 | Liftoff | | gene | 77507910 | | 77576435 | IPO8 |
| 5 | 79075001 | 79125000 | 5 | Liftoff | | gene | 78946404 | | 79119937 | FAR2 |
| 5 | 97350001 | 97400000 | 5 | Liftoff | | gene | 97352486 | | 97353423 | LOC101120486 |
| 5 | 97350001 | 97400000 | 5 | Liftoff | | gene | 97360008 | | 97360946 | LOC101120742 |
| 5 | 97350001 | 97400000 | 5 | Liftoff | | gene | 97370011 | | 97372383 | SMIM10L1 |
| 5 | 97350001 | 97400000 | 5 | Liftoff | | gene | 97399664 | | 97400580 | LOC101121003 |
| 5 | 99000001 | 99050000 | 5 | Liftoff | | gene | 99039255 | | 99059016 | LOC101122014 |
| 6 | 103975001 | 104025000 | 6 | Liftoff | | gene | 103934122 | | 104028755 | EVC |
| 6 | 104000001 | 104050000 | 6 | Liftoff | | gene | 103934122 | | 104028755 | EVC |
| 6 | 104000001 | 104050000 | 6 | Liftoff | | gene | 104031984 | | 104189350 | EVC2 |
| 6 | 104075001 | 104125000 | 6 | Liftoff | | gene | 104031984 | | 104189350 | EVC2 |
| 6 | 104100001 | 104150000 | 6 | Liftoff | | gene | 104031984 | | 104189350 | EVC2 |
| 6 | 105175001 | 105225000 | 6 | Liftoff | | gene | 105092153 | | 105211005 | STX18 |
| 6 | 105175001 | 105225000 | 6 | Liftoff | | gene | 105210652 | | 105244336 | NSG1 |
| 6 | 115800001 | 115850000 | 6 | Liftoff | | gene | 115813077 | | 115841076 | DOK7 |
| 6 | 115800001 | 115850000 | 6 | Liftoff | | gene | 115848793 | | 115857395 | HGFAC |
| 6 | 115825001 | 115875000 | 6 | Liftoff | | gene | 115813077 | | 115841076 | DOK7 |
| 6 | 115825001 | 115875000 | 6 | Liftoff | | gene | 115848793 | | 115857395 | HGFAC |
| 6 | 115825001 | 115875000 | 6 | Liftoff | | gene | 115863035 | | 115950251 | RGS12 |
| 6 | 115850001 | 115900000 | 6 | Liftoff | | gene | 115848793 | | 115857395 | HGFAC |
| 6 | 115850001 | 115900000 | 6 | Liftoff | | gene | 115863035 | | 115950251 | RGS12 |
| 6 | 116075001 | 116125000 | 6 | Liftoff | | gene | 116030591 | | 116154665 | HTT |
| 6 | 116100001 | 116150000 | 6 | Liftoff | | gene | 116030591 | | 116154665 | HTT |
| 6 | 16250001 | 16300000 | 6 | Liftoff | | gene | 16236386 | | 16315888 | SEC24B |
| 6 | 23200001 | 23250000 | 6 | Liftoff | | gene | 23036460 | | 23366156 | BANK1 |
| 6 | 23225001 | 23275000 | 6 | Liftoff | | gene | 23036460 | | 23366156 | BANK1 |
| 6 | 25250001 | 25300000 | 6 | Liftoff | | gene | 25297169 | | 25316043 | C6H4orf54 |
| 6 | 38000001 | 38050000 | 6 | Liftoff | | gene | 38003204 | | 38171099 | LCORL |
| 6 | 38025001 | 38075000 | 6 | Liftoff | | gene | 38003204 | | 38171099 | LCORL |
| 6 | 38050001 | 38100000 | 6 | Liftoff | | gene | 38003204 | | 38171099 | LCORL |
| 6 | 38075001 | 38125000 | 6 | Liftoff | | gene | 38003204 | | 38171099 | LCORL |
| 6 | 38100001 | 38150000 | 6 | Liftoff | | gene | 38003204 | | 38171099 | LCORL |
| 6 | 38125001 | 38175000 | 6 | Liftoff | | gene | 38003204 | | 38171099 | LCORL |
| 6 | 450001 | 500000 | 6 | Liftoff | | gene | 435564 | | 457592 | APELA |
| 6 | 45325001 | 45375000 | 6 | Liftoff | | gene | 45320815 | | 45356018 | PI4K2B |
| 6 | 45325001 | 45375000 | 6 | Liftoff | | gene | 45338512 | | 45353674 | LOC114115296 |
| 6 | 45350001 | 45400000 | 6 | Liftoff | | gene | 45320815 | | 45356018 | PI4K2B |
| 6 | 45350001 | 45400000 | 6 | Liftoff | | gene | 45338512 | | 45353674 | LOC114115296 |
| 6 | 45350001 | 45400000 | 6 | Liftoff | | gene | 45379781 | | 45439384 | ZCCHC4 |
| 6 | 45375001 | 45425000 | 6 | Liftoff | | gene | 45379781 | | 45439384 | ZCCHC4 |
| 6 | 45400001 | 45450000 | 6 | Liftoff | | gene | 45379781 | | 45439384 | ZCCHC4 |
| 6 | 45400001 | 45450000 | 6 | Liftoff | | gene | 45445812 | | 45480180 | ANAPC4 |
| 6 | 57600001 | 57650000 | 6 | Liftoff | | gene | 57582668 | | 57604234 | C6H4orf19 |
| 6 | 57600001 | 57650000 | 6 | Liftoff | | gene | 57619654 | | 57695866 | RELL1 |
| 6 | 57650001 | 57700000 | 6 | Liftoff | | gene | 57619654 | | 57695866 | RELL1 |
| 6 | 57675001 | 57725000 | 6 | Liftoff | | gene | 57619654 | | 57695866 | RELL1 |
| 6 | 58800001 | 58850000 | 6 | Liftoff | | gene | 58772493 | | 58842806 | FAM114A1 |
| 6 | 59575001 | 59625000 | 6 | Liftoff | | gene | 59576555 | | 59704739 | PDS5A |
| 6 | 59600001 | 59650000 | 6 | Liftoff | | gene | 59576555 | | 59704739 | PDS5A |
| 6 | 59800001 | 59850000 | 6 | Liftoff | | gene | 59783258 | | 59856566 | N4BP2 |
| 6 | 59825001 | 59875000 | 6 | Liftoff | | gene | 59783258 | | 59856566 | N4BP2 |
| 6 | 59900001 | 59950000 | 6 | Liftoff | | gene | 59896215 | | 59945491 | RHOH |
| 6 | 60325001 | 60375000 | 6 | Liftoff | | gene | 60342267 | | 60390217 | NSUN7 |
| 6 | 60350001 | 60400000 | 6 | Liftoff | | gene | 60342267 | | 60390217 | NSUN7 |
| 6 | 60500001 | 60550000 | 6 | Liftoff | | gene | 60392030 | | 60769416 | APBB2 |
| 6 | 60525001 | 60575000 | 6 | Liftoff | | gene | 60392030 | | 60769416 | APBB2 |
| 6 | 60550001 | 60600000 | 6 | Liftoff | | gene | 60392030 | | 60769416 | APBB2 |
| 6 | 61125001 | 61175000 | 6 | Liftoff | | gene | 60897899 | | 61257758 | LIMCH1 |
| 6 | 61150001 | 61200000 | 6 | Liftoff | | gene | 60897899 | | 61257758 | LIMCH1 |
| 6 | 61175001 | 61225000 | 6 | Liftoff | | gene | 60897899 | | 61257758 | LIMCH1 |
| 6 | 63175001 | 63225000 | 6 | Liftoff | | gene | 63177948 | | 63179990 | LOC101113221 |
| 6 | 68375001 | 68425000 | 6 | Liftoff | | gene | 68323831 | | 68400305 | DCUN1D4 |
| 6 | 68900001 | 68950000 | 6 | Liftoff | | gene | 68902369 | | 68976543 | USP46 |
| 6 | 69350001 | 69400000 | 6 | Liftoff | | gene | 69136261 | | 69536688 | SCFD2 |
| 6 | 69375001 | 69425000 | 6 | Liftoff | | gene | 69136261 | | 69536688 | SCFD2 |
| 6 | 69675001 | 69725000 | 6 | Liftoff | | gene | 69614256 | | 69805063 | LNX1 |
| 6 | 71600001 | 71650000 | 6 | Liftoff | | gene | 71619972 | | 71656458 | PDCL2 |
| 6 | 81775001 | 81825000 | 6 | Liftoff | | gene | 81668535 | | 81839085 | LOC114115236 |
| 6 | 81800001 | 81850000 | 6 | Liftoff | | gene | 81668535 | | 81839085 | LOC114115236 |
| 6 | 84900001 | 84950000 | 6 | Liftoff | | gene | 84904267 | | 84920152 | UGT2B7 |
| 6 | 84925001 | 84975000 | 6 | Liftoff | | gene | 84969748 | | 84990960 | LOC114115343 |
| 6 | 91575001 | 91625000 | 6 | Liftoff | | gene | 91567485 | | 91599806 | ART3 |
| 6 | 91575001 | 91625000 | 6 | Liftoff | | gene | 91602058 | | 91633825 | NUP54 |
| 6 | 93050001 | 93100000 | 6 | Liftoff | | gene | 92973562 | | 93090355 | CNOT6L |
| 6 | 94075001 | 94125000 | 6 | Liftoff | | gene | 94085263 | | 94206236 | BMP2K |
| 6 | 95975001 | 96025000 | 6 | Liftoff | | gene | 95866663 | | 96294573 | CFAP299 |
| 7 | 100075001 | 100125000 | 7 | Liftoff | | gene | 99957765 | | 100132178 | PAM |
| 7 | 16250001 | 16300000 | 7 | Liftoff | | gene | 16257623 | | 16267277 | DENND1C |
| 7 | 16250001 | 16300000 | 7 | Liftoff | | gene | 16267280 | | 16270323 | CRB3 |
| 7 | 16250001 | 16300000 | 7 | Liftoff | | gene | 16271704 | | 16272556 | LOC114114835 |
| 7 | 16250001 | 16300000 | 7 | Liftoff | | gene | 16272676 | | 16286393 | SLC25A23 |
| 7 | 16250001 | 16300000 | 7 | Liftoff | | gene | 16286501 | | 16296441 | SLC25A41 |
| 7 | 16250001 | 16300000 | 7 | Liftoff | | gene | 16292910 | | 16297522 | LOC105609861 |
| 7 | 16250001 | 16300000 | 7 | Liftoff | | gene | 16297727 | | 16309256 | KHSRP |
| 7 | 16275001 | 16325000 | 7 | Liftoff | | gene | 16272676 | | 16286393 | SLC25A23 |
| 7 | 16275001 | 16325000 | 7 | Liftoff | | gene | 16286501 | | 16296441 | SLC25A41 |
| 7 | 16275001 | 16325000 | 7 | Liftoff | | gene | 16292910 | | 16297522 | LOC105609861 |
| 7 | 16275001 | 16325000 | 7 | Liftoff | | gene | 16297727 | | 16309256 | KHSRP |
| 7 | 16275001 | 16325000 | 7 | Liftoff | | gene | 16311458 | | 16322833 | LOC114114997 |
| 7 | 16300001 | 16350000 | 7 | Liftoff | | gene | 16297727 | | 16309256 | KHSRP |
| 7 | 16300001 | 16350000 | 7 | Liftoff | | gene | 16311458 | | 16322833 | LOC114114997 |
| 7 | 16300001 | 16350000 | 7 | Liftoff | | gene | 16324449 | | 16330594 | GTF2F1 |
| 7 | 16300001 | 16350000 | 7 | Liftoff | | gene | 16334142 | | 16334687 | PSPN |
| 7 | 16300001 | 16350000 | 7 | Liftoff | | gene | 16334963 | | 16336604 | ALKBH7 |
| 7 | 16300001 | 16350000 | 7 | Liftoff | | gene | 16340578 | | 16345841 | CLPP |
| 7 | 16325001 | 16375000 | 7 | Liftoff | | gene | 16324449 | | 16330594 | GTF2F1 |
| 7 | 16325001 | 16375000 | 7 | Liftoff | | gene | 16334142 | | 16334687 | PSPN |
| 7 | 16325001 | 16375000 | 7 | Liftoff | | gene | 16334963 | | 16336604 | ALKBH7 |
| 7 | 16325001 | 16375000 | 7 | Liftoff | | gene | 16340578 | | 16345841 | CLPP |
| 7 | 16325001 | 16375000 | 7 | Liftoff | | gene | 16367288 | | 16391387 | ACER1 |
| 7 | 16450001 | 16500000 | 7 | Liftoff | | gene | 16403784 | | 16468595 | MLLT1 |
| 7 | 16450001 | 16500000 | 7 | Liftoff | | gene | 16480368 | | 16518055 | ACSBG2 |
| 7 | 16475001 | 16525000 | 7 | Liftoff | | gene | 16480368 | | 16518055 | ACSBG2 |
| 7 | 17775001 | 17825000 | 7 | Liftoff | | gene | 17778227 | | 17791555 | SEMA6B |
| 7 | 17775001 | 17825000 | 7 | Liftoff | | gene | 17803821 | | 17807074 | LRG1 |
| 7 | 17775001 | 17825000 | 7 | Liftoff | | gene | 17808044 | | 17819903 | PLIN5 |
| 7 | 17775001 | 17825000 | 7 | Liftoff | | gene | 17822749 | | 17833874 | PLIN4 |
| 7 | 17800001 | 17850000 | 7 | Liftoff | | gene | 17803821 | | 17807074 | LRG1 |
| 7 | 17800001 | 17850000 | 7 | Liftoff | | gene | 17808044 | | 17819903 | PLIN5 |
| 7 | 17800001 | 17850000 | 7 | Liftoff | | gene | 17822749 | | 17833874 | PLIN4 |
| 7 | 17800001 | 17850000 | 7 | Liftoff | | gene | 17836384 | | 17852603 | HDGFL2 |
| 7 | 18150001 | 18200000 | 7 | Liftoff | | gene | 18154632 | | 18175280 | ZBTB7A |
| 7 | 18150001 | 18200000 | 7 | Liftoff | | gene | 18177351 | | 18201033 | PIAS4 |
| 7 | 19500001 | 19550000 | 7 | Liftoff | | gene | 19517096 | | 19568929 | DOT1L |
| 7 | 19525001 | 19575000 | 7 | Liftoff | | gene | 19517096 | | 19568929 | DOT1L |
| 7 | 19525001 | 19575000 | 7 | Liftoff | | gene | 19569486 | | 19572193 | PLEKHJ1 |
| 7 | 19525001 | 19575000 | 7 | Liftoff | | gene | 19572248 | | 19580969 | SF3A2 |
| 7 | 19575001 | 19625000 | 7 | Liftoff | | gene | 19572248 | | 19580969 | SF3A2 |
| 7 | 19575001 | 19625000 | 7 | Liftoff | | gene | 19581627 | | 19584482 | AMH |
| 7 | 19575001 | 19625000 | 7 | Liftoff | | gene | 19584681 | | 19588553 | JSRP1 |
| 7 | 19575001 | 19625000 | 7 | Liftoff | | gene | 19593893 | | 19627242 | AP3D1 |
| 7 | 19600001 | 19650000 | 7 | Liftoff | | gene | 19593893 | | 19627242 | AP3D1 |
| 7 | 19600001 | 19650000 | 7 | Liftoff | | gene | 19628705 | | 19631825 | IZUMO4 |
| 7 | 19600001 | 19650000 | 7 | Liftoff | | gene | 19632081 | | 19651118 | MOB3A |
| 7 | 19750001 | 19800000 | 7 | Liftoff | | gene | 19744286 | | 19771728 | 8-Sep |
| 7 | 19750001 | 19800000 | 7 | Liftoff | | gene | 19771426 | | 19775103 | CCNI2 |
| 7 | 19750001 | 19800000 | 7 | Liftoff | | gene | 19780271 | | 19799322 | LOC114114998 |
| 7 | 19775001 | 19825000 | 7 | Liftoff | | gene | 19780271 | | 19799322 | LOC114114998 |
| 7 | 2175001 | 2225000 | 7 | Liftoff | | gene | 2183741 | | 2231761 | LOC101123551 |
| 7 | 23975001 | 24025000 | 7 | Liftoff | | gene | 23909774 | | 24005477 | SLC12A2 |
| 7 | 24250001 | 24300000 | 7 | Liftoff | | gene | 24155640 | | 24368141 | CCDC192 |
| 7 | 24825001 | 24875000 | 7 | Liftoff | | gene | 24859700 | | 24924924 | LOC114114854 |
| 7 | 28750001 | 28800000 | 7 | Liftoff | | gene | 28700306 | | 28814667 | PRDM6 |
| 7 | 28775001 | 28825000 | 7 | Liftoff | | gene | 28700306 | | 28814667 | PRDM6 |
| 7 | 28775001 | 28825000 | 7 | Liftoff | | gene | 28815186 | | 28817597 | LOC114114999 |
| 7 | 40600001 | 40650000 | 7 | Liftoff | | gene | 40617678 | | 40618612 | LOC101103645 |
| 7 | 40600001 | 40650000 | 7 | Liftoff | | gene | 40638169 | | 40648812 | LOC101103895 |
| 7 | 43900001 | 43950000 | 7 | Liftoff | | gene | 43910341 | | 43942534 | TCF7 |
| 7 | 43900001 | 43950000 | 7 | Liftoff | | gene | 43945792 | | 43958055 | SKP1 |
| 7 | 55525001 | 55575000 | 7 | Liftoff | | gene | 55514217 | | 55583272 | LARS |
| 7 | 55550001 | 55600000 | 7 | Liftoff | | gene | 55514217 | | 55583272 | LARS |
| 7 | 57175001 | 57225000 | 7 | Liftoff | | gene | 57221225 | | 57227922 | SPINK1 |
| 7 | 57825001 | 57875000 | 7 | Liftoff | | gene | 57869416 | | 57872967 | LOC101116828 |
| 7 | 58050001 | 58100000 | 7 | Liftoff | | gene | 58019271 | | 58086142 | FBXO38 |
| 7 | 58050001 | 58100000 | 7 | Liftoff | | gene | 58094436 | | 58113495 | LOC114114893 |
| 7 | 59425001 | 59475000 | 7 | Liftoff | | gene | 59421245 | | 59544345 | PDE6A |
| 7 | 59450001 | 59500000 | 7 | Liftoff | | gene | 59421245 | | 59544345 | PDE6A |
| 7 | 59475001 | 59525000 | 7 | Liftoff | | gene | 59421245 | | 59544345 | PDE6A |
| 7 | 59475001 | 59525000 | 7 | Liftoff | | gene | 59509907 | | 59532198 | SLC26A2 |
| 7 | 59575001 | 59625000 | 7 | Liftoff | | gene | 59544520 | | 59606425 | HMGXB3 |
| 7 | 59575001 | 59625000 | 7 | Liftoff | | gene | 59606554 | | 59638217 | CSF1R |
| 7 | 63300001 | 63350000 | 7 | Liftoff | | gene | 63025770 | | 63382413 | GRIA1 |
| 7 | 63525001 | 63575000 | 7 | Liftoff | | gene | 63557522 | | 63594436 | FAM114A2 |
| 7 | 79425001 | 79475000 | 7 | Liftoff | | gene | 79399534 | | 79454961 | LOC106990145 |
| 7 | 79425001 | 79475000 | 7 | Liftoff | | gene | 79401169 | | 79443487 | ACOT12 |
| 7 | 81225001 | 81275000 | 7 | Liftoff | | gene | 81007159 | | 81294608 | XRCC4 |
| 7 | 86050001 | 86100000 | 7 | Liftoff | | gene | 86024426 | | 86064644 | LOC114114910 |
| 7 | 86350001 | 86400000 | 7 | Liftoff | | gene | 86338531 | | 86516489 | MEF2C |
| 7 | 8750001 | 8800000 | 7 | Liftoff | | gene | 8771010 | | 8771993 | LOC101111963 |
| 7 | 8750001 | 8800000 | 7 | Liftoff | | gene | 8792277 | | 8799505 | LOC101112479 |
| 8 | 101025001 | 101075000 | 8 | Liftoff | | gene | 101072407 | | 101084303 | TXN |
| 8 | 104975001 | 105025000 | 8 | Liftoff | | gene | 105003993 | | 105013070 | ATP6V1G1 |
| 8 | 105000001 | 105050000 | 8 | Liftoff | | gene | 105003993 | | 105013070 | ATP6V1G1 |
| 8 | 105000001 | 105050000 | 8 | Liftoff | | gene | 105024990 | | 105050189 | TMEM268 |
| 8 | 11575001 | 11625000 | 8 | Liftoff | | gene | 11608530 | | 11609996 | LOC114113729 |
| 8 | 11600001 | 11650000 | 8 | Liftoff | | gene | 11608530 | | 11609996 | LOC114113729 |
| 8 | 25400001 | 25450000 | 8 | Liftoff | | gene | 25329223 | | 25825414 | ADAMTSL1 |
| 8 | 25425001 | 25475000 | 8 | Liftoff | | gene | 25329223 | | 25825414 | ADAMTSL1 |
| 8 | 27800001 | 27850000 | 8 | Liftoff | | gene | 27476025 | | 27960542 | BNC2 |
| 8 | 44300001 | 44350000 | 8 | Liftoff | | gene | 44299572 | | 44504138 | PGM5 |
| 8 | 60475001 | 60525000 | 8 | Liftoff | | gene | 60429273 | | 60557297 | RNF38 |
| 8 | 60475001 | 60525000 | 8 | Liftoff | | gene | 60485144 | | 60556945 | LOC114113068 |
| 8 | 60500001 | 60550000 | 8 | Liftoff | | gene | 60429273 | | 60557297 | RNF38 |
| 8 | 60500001 | 60550000 | 8 | Liftoff | | gene | 60485144 | | 60556945 | LOC114113068 |
| 8 | 66900001 | 66950000 | 8 | Liftoff | | gene | 66903015 | | 66903150 | LOC114113463 |
| 8 | 69050001 | 69100000 | 8 | Liftoff | | gene | 69062428 | | 69098088 | XPO7 |
| 8 | 69075001 | 69125000 | 8 | Liftoff | | gene | 69062428 | | 69098088 | XPO7 |
| 8 | 69075001 | 69125000 | 8 | Liftoff | | gene | 69107630 | | 69120563 | NPM2 |
| 8 | 69650001 | 69700000 | 8 | Liftoff | | gene | 69645896 | | 69652489 | C2H8orf58 |
| 8 | 69650001 | 69700000 | 8 | Liftoff | | gene | 69651070 | | 69668119 | CCAR2 |
| 8 | 69650001 | 69700000 | 8 | Liftoff | | gene | 69668401 | | 69718913 | BIN3 |
| 8 | 70725001 | 70775000 | 8 | Liftoff | | gene | 70722491 | | 70726903 | NKX2-6 |
| 8 | 74200001 | 74250000 | 8 | Liftoff | | gene | 74206928 | | 74230906 | STMN4 |
| 8 | 74525001 | 74575000 | 8 | Liftoff | | gene | 74462369 | | 74537358 | EPHX2 |
| 8 | 74525001 | 74575000 | 8 | Liftoff | | gene | 74544416 | | 74569271 | LOC101106395 |
| 8 | 74725001 | 74775000 | 8 | Liftoff | | gene | 74740210 | | 74790515 | B4GALT1 |
| 8 | 74750001 | 74800000 | 8 | Liftoff | | gene | 74740210 | | 74790515 | B4GALT1 |
| 8 | 74750001 | 74800000 | 8 | Liftoff | | gene | 74790911 | | 74822028 | LOC105607270 |
| 8 | 74800001 | 74850000 | 8 | Liftoff | | gene | 74790911 | | 74822028 | LOC105607270 |
| 8 | 74850001 | 74900000 | 8 | Liftoff | | gene | 74878353 | | 74885879 | SPINK4 |
| 8 | 74850001 | 74900000 | 8 | Liftoff | | gene | 74892920 | | 74904885 | BAG1 |
| 8 | 74875001 | 74925000 | 8 | Liftoff | | gene | 74878353 | | 74885879 | SPINK4 |
| 8 | 74875001 | 74925000 | 8 | Liftoff | | gene | 74892920 | | 74904885 | BAG1 |
| 8 | 74875001 | 74925000 | 8 | Liftoff | | gene | 74905179 | | 74918770 | CHMP5 |
| 8 | 74875001 | 74925000 | 8 | Liftoff | | gene | 74914552 | | 74916880 | LOC105607262 |
| 8 | 74900001 | 74950000 | 8 | Liftoff | | gene | 74892920 | | 74904885 | BAG1 |
| 8 | 74900001 | 74950000 | 8 | Liftoff | | gene | 74905179 | | 74918770 | CHMP5 |
| 8 | 74900001 | 74950000 | 8 | Liftoff | | gene | 74914552 | | 74916880 | LOC105607262 |
| 8 | 74900001 | 74950000 | 8 | Liftoff | | gene | 74929122 | | 74992860 | NFX1 |
| 8 | 74925001 | 74975000 | 8 | Liftoff | | gene | 74929122 | | 74992860 | NFX1 |
| 8 | 74950001 | 75000000 | 8 | Liftoff | | gene | 74929122 | | 74992860 | NFX1 |
| 8 | 75000001 | 75050000 | 8 | Liftoff | | gene | 75003270 | | 75021445 | AQP7 |
| 8 | 75025001 | 75075000 | 8 | Liftoff | | gene | 75060114 | | 75066023 | AQP3 |
| 8 | 75050001 | 75100000 | 8 | Liftoff | | gene | 75060114 | | 75066023 | AQP3 |
| 8 | 75050001 | 75100000 | 8 | Liftoff | | gene | 75075058 | | 75087220 | NOL6 |
| 8 | 75075001 | 75125000 | 8 | Liftoff | | gene | 75075058 | | 75087220 | NOL6 |
| 8 | 75075001 | 75125000 | 8 | Liftoff | | gene | 75101845 | | 75201817 | UBE2R2 |
| 8 | 75100001 | 75150000 | 8 | Liftoff | | gene | 75101845 | | 75201817 | UBE2R2 |
| 8 | 75125001 | 75175000 | 8 | Liftoff | | gene | 75101845 | | 75201817 | UBE2R2 |
| 8 | 75150001 | 75200000 | 8 | Liftoff | | gene | 75101845 | | 75201817 | UBE2R2 |
| 8 | 75175001 | 75225000 | 8 | Liftoff | | gene | 75101845 | | 75201817 | UBE2R2 |
| 8 | 75175001 | 75225000 | 8 | Liftoff | | gene | 75203021 | | 75317452 | UBAP2 |
| 8 | 75175001 | 75225000 | 8 | Liftoff | | gene | 75215063 | | 75215145 | LOC114113432 |
| 8 | 75200001 | 75250000 | 8 | Liftoff | | gene | 75203021 | | 75317452 | UBAP2 |
| 8 | 75200001 | 75250000 | 8 | Liftoff | | gene | 75215063 | | 75215145 | LOC114113432 |
| 8 | 75200001 | 75250000 | 8 | Liftoff | | gene | 75234070 | | 75234150 | LOC114113431 |
| 8 | 75225001 | 75275000 | 8 | Liftoff | | gene | 75203021 | | 75317452 | UBAP2 |
| 8 | 75225001 | 75275000 | 8 | Liftoff | | gene | 75234070 | | 75234150 | LOC114113431 |
| 8 | 75275001 | 75325000 | 8 | Liftoff | | gene | 75203021 | | 75317452 | UBAP2 |
| 8 | 75300001 | 75350000 | 8 | Liftoff | | gene | 75203021 | | 75317452 | UBAP2 |
| 8 | 75325001 | 75375000 | 8 | Liftoff | | gene | 75357968 | | 75393783 | DCAF12 |
| 8 | 75350001 | 75400000 | 8 | Liftoff | | gene | 75357968 | | 75393783 | DCAF12 |
| 8 | 75375001 | 75425000 | 8 | Liftoff | | gene | 75357968 | | 75393783 | DCAF12 |
| 8 | 75375001 | 75425000 | 8 | Liftoff | | gene | 75413767 | | 75413873 | LOC114111088 |
| 8 | 8775001 | 8825000 | 8 | Liftoff | | gene | 8615896 | | 8995707 | MSRA |
| 8 | 9750001 | 9800000 | 8 | Liftoff | | gene | 9671014 | | 9804621 | INTS9 |
| 8 | 9775001 | 9825000 | 8 | Liftoff | | gene | 9671014 | | 9804621 | INTS9 |
| 8 | 9775001 | 9825000 | 8 | Liftoff | | gene | 9802535 | | 9804331 | LOC114113028 |
| 8 | 9800001 | 9850000 | 8 | Liftoff | | gene | 9802535 | | 9804331 | LOC114113028 |
| 8 | 9800001 | 9850000 | 8 | Liftoff | | gene | 9814769 | | 9947370 | EXTL3 |
| 9 | 10125001 | 10175000 | 9 | Liftoff | | gene | 10106756 | | 10266478 | UBE3D |
| 9 | 10150001 | 10200000 | 9 | Liftoff | | gene | 10106756 | | 10266478 | UBE3D |
| 9 | 11250001 | 11300000 | 9 | Liftoff | | gene | 11239666 | | 11296327 | ECHDC1 |
| 9 | 11250001 | 11300000 | 9 | Liftoff | | gene | 11289054 | | 11289133 | TRNAS-AGA-2 |
| 9 | 11250001 | 11300000 | 9 | Liftoff | | gene | 11296788 | | 11319211 | RNF146 |
| 9 | 21450001 | 21500000 | 9 | Liftoff | | gene | 21465380 | | 21467869 | LOC114116187 |
| 9 | 21450001 | 21500000 | 9 | Liftoff | | gene | 21497718 | | 21500403 | LOC101111266 |
| 9 | 21450001 | 21500000 | 9 | Liftoff | | gene | 21499041 | | 21502995 | LOC114116186 |
| 9 | 21475001 | 21525000 | 9 | Liftoff | | gene | 21497718 | | 21500403 | LOC101111266 |
| 9 | 21475001 | 21525000 | 9 | Liftoff | | gene | 21499041 | | 21502995 | LOC114116186 |
| 9 | 25850001 | 25900000 | 9 | Liftoff | | gene | 25867395 | | 26085226 | FYN |
| 9 | 27475001 | 27525000 | 9 | Liftoff | | gene | 27502875 | | 27504770 | GPR6 |
| 9 | 27500001 | 27550000 | 9 | Liftoff | | gene | 27502875 | | 27504770 | GPR6 |
| 9 | 30675001 | 30725000 | 9 | Liftoff | | gene | 30630628 | | 30858022 | CRYBG1 |
| 9 | 36425001 | 36475000 | 9 | Liftoff | | gene | 36317838 | | 36662634 | ASCC3 |
| 9 | 41500001 | 41550000 | 9 | Liftoff | | gene | 41506365 | | 41587111 | MANEA |
| 9 | 41525001 | 41575000 | 9 | Liftoff | | gene | 41506365 | | 41587111 | MANEA |
| 9 | 41550001 | 41600000 | 9 | Liftoff | | gene | 41506365 | | 41587111 | MANEA |
| 9 | 41575001 | 41625000 | 9 | Liftoff | | gene | 41506365 | | 41587111 | MANEA |
| 9 | 49500001 | 49550000 | 9 | Liftoff | | gene | 49506440 | | 49529260 | AKIRIN2 |
| 9 | 49500001 | 49550000 | 9 | Liftoff | | gene | 49534201 | | 49607026 | ORC3 |
| 9 | 49525001 | 49575000 | 9 | Liftoff | | gene | 49506440 | | 49529260 | AKIRIN2 |
| 9 | 49525001 | 49575000 | 9 | Liftoff | | gene | 49534201 | | 49607026 | ORC3 |
| 9 | 49625001 | 49675000 | 9 | Liftoff | | gene | 49607137 | | 49680258 | RARS2 |
| 9 | 50000001 | 50050000 | 9 | Liftoff | | gene | 50028748 | | 50047140 | CGA |
| 9 | 63325001 | 63375000 | 9 | Liftoff | | gene | 63316032 | | 63390758 | NHSL1 |
| 9 | 7000001 | 7050000 | 9 | Liftoff | | gene | 7042851 | | 7085292 | TTK |
| 9 | 70450001 | 70500000 | 9 | Liftoff | | gene | 70317871 | | 70759403 | GRM1 |
| 9 | 70475001 | 70525000 | 9 | Liftoff | | gene | 70317871 | | 70759403 | GRM1 |
| 9 | 71650001 | 71700000 | 9 | Liftoff | | gene | 71545213 | | 71716135 | STXBP5 |
| 9 | 71650001 | 71700000 | 9 | Liftoff | | gene | 71681609 | | 71684634 | LOC114116111 |
| 9 | 79725001 | 79775000 | 9 | Liftoff | | gene | 79723560 | | 79939218 | SCAF8 |
| 9 | 84525001 | 84575000 | 9 | Liftoff | | gene | 84516410 | | 84619971 | IGF2R |
| 9 | 84550001 | 84600000 | 9 | Liftoff | | gene | 84516410 | | 84619971 | IGF2R |
| 9 | 90150001 | 90200000 | 9 | Liftoff | | gene | 90179884 | | 90182219 | CCR6 |
| 9 | 90150001 | 90200000 | 9 | Liftoff | | gene | 90195675 | | 90196625 | GPR31 |
| 9 | 90175001 | 90225000 | 9 | Liftoff | | gene | 90179884 | | 90182219 | CCR6 |
| 9 | 90175001 | 90225000 | 9 | Liftoff | | gene | 90195675 | | 90196625 | GPR31 |
| 9 | 90175001 | 90225000 | 9 | Liftoff | | gene | 90208263 | | 90226861 | LOC105612542 |
| 9 | 92200001 | 92250000 | 9 | Liftoff | | gene | 92189260 | | 92256673 | FAM120B |
| 9 | 92225001 | 92275000 | 9 | Liftoff | | gene | 92189260 | | 92256673 | FAM120B |
| 9 | 92275001 | 92325000 | 9 | Liftoff | | gene | 92315071 | | 92336595 | PSMB1 |
| 9 | 92300001 | 92350000 | 9 | Liftoff | | gene | 92315071 | | 92336595 | PSMB1 |
| 9 | 92300001 | 92350000 | 9 | Liftoff | | gene | 92341208 | | 92353918 | TBP |

**Table S6.** Significantly enriched GO entries (*P*< 0.05)

| ID | Descrption | class | qvalue | num |
| --- | --- | --- | --- | --- |
| GO:0043231 | intracellular membrane-bounded organelle | Cellular Component | 5.78539E-07 | 298 |
| GO:0043227 | membrane-bounded organelle | Cellular Component | 5.78539E-07 | 299 |
| GO:0005622 | intracellular | Cellular Component | 8.68208E-06 | 368 |
| GO:0044424 | intracellular part | Cellular Component | 8.68208E-06 | 368 |
| GO:0005634 | nucleus | Cellular Component | 7.61326E-05 | 198 |
| GO:0043226 | organelle | Cellular Component | 0.000764821 | 327 |
| GO:0043229 | intracellular organelle | Cellular Component | 0.000764821 | 322 |
| GO:0005488 | binding | Molecular Function | 0.001312506 | 375 |
| GO:0044428 | nuclear part | Cellular Component | 0.005042536 | 136 |
| GO:0043233 | organelle lumen | Cellular Component | 0.007501292 | 132 |
| GO:0031981 | nuclear lumen | Cellular Component | 0.00845387 | 124 |
| GO:0070013 | intracellular organelle lumen | Cellular Component | 0.00845387 | 131 |
| GO:0031974 | membrane-enclosed lumen | Cellular Component | 0.00845387 | 133 |
| GO:0005654 | nucleoplasm | Cellular Component | 0.01501534 | 112 |
| GO:0044446 | intracellular organelle part | Cellular Component | 0.032180235 | 217 |
| GO:0044422 | organelle part | Cellular Component | 0.033944727 | 222 |
| GO:0048523 | negative regulation of cellular process | Biological Process | 0.040632137 | 138 |
| GO:0048518 | positive regulation of biological process | Biological Process | 0.040632137 | 170 |
| GO:1901575 | organic substance catabolic process | Biological Process | 0.040632137 | 65 |
| GO:0006511 | ubiquitin-dependent protein catabolic process | Biological Process | 0.040632137 | 28 |
| GO:0044265 | cellular macromolecule catabolic process | Biological Process | 0.040632137 | 39 |
| GO:0048522 | positive regulation of cellular process | Biological Process | 0.040632137 | 151 |
| GO:0009893 | positive regulation of metabolic process | Biological Process | 0.040632137 | 115 |
| GO:0019941 | modification-dependent protein catabolic process | Biological Process | 0.040632137 | 28 |
| GO:0009057 | macromolecule catabolic process | Biological Process | 0.040632137 | 45 |
| GO:0043632 | modification-dependent macromolecule catabolic process | Biological Process | 0.043045982 | 28 |
| GO:0051603 | proteolysis involved in cellular protein catabolic process | Biological Process | 0.043306495 | 31 |
| GO:0034502 | protein localization to chromosome | Biological Process | 0.044478328 | 9 |
| GO:0048519 | negative regulation of biological process | Biological Process | 0.044478328 | 142 |
| GO:0010604 | positive regulation of macromolecule metabolic process | Biological Process | 0.044478328 | 101 |
| GO:0044257 | cellular protein catabolic process | Biological Process | 0.044478328 | 32 |
| GO:0070647 | protein modification by small protein conjugation or removal | Biological Process | 0.047158353 | 41 |
| GO:0070419 | nonhomologous end joining complex | Cellular Component | 0.047843987 | 3 |
| GO:0005737 | cytoplasm | Cellular Component | 0.047843987 | 263 |
| GO:0030057 | desmosome | Cellular Component | 0.047843987 | 4 |
| GO:0030163 | protein catabolic process | Biological Process | 0.048373713 | 37 |
| GO:0031325 | positive regulation of cellular metabolic process | Biological Process | 0.048373713 | 100 |
| GO:0019222 | regulation of metabolic process | Biological Process | 0.048717658 | 186 |
| GO:0080090 | regulation of primary metabolic process | Biological Process | 0.048717658 | 164 |
| GO:0042177 | negative regulation of protein catabolic process | Biological Process | 0.049069901 | 12 |

**Table S7.** Significantly enriched KEGG pathways (*P*< 0.05)

| ID | Descrption | class | pvalue | fg_num | bg_num |
| --- | --- | --- | --- | --- | --- |
| ko04110 | Cell cycle | Cellular Processes | 4.18E-05 | 14 | 140 |
| ko04114 | Oocyte meiosis | Cellular Processes | 0.003859526 | 10 | 130 |
| ko03450 | Non-homologous end-joining | Genetic Information Processing | 0.005253959 | 3 | 13 |
| ko04923 | Regulation of lipolysis in adipocytes | Organismal Systems | 0.00535991 | 6 | 57 |
| ko04024 | cAMP signaling pathway | Environmental Information Processing | 0.007108369 | 14 | 235 |
| ko00630 | Glyoxylate and dicarboxylate metabolism | Metabolism | 0.02404695 | 4 | 39 |
| ko05017 | Spinocerebellar ataxia | Human Diseases | 0.0271372 | 7 | 103 |
| ko04622 | RIG-I-like receptor signaling pathway | Organismal Systems | 0.03040822 | 6 | 83 |
| ko04929 | GnRH secretion | Organismal Systems | 0.03100786 | 5 | 62 |
| ko04720 | Long-term potentiation | Organismal Systems | 0.03292937 | 5 | 63 |
| ko00513 | Various types of N-glycan biosynthesis | Metabolism | 0.03306146 | 4 | 43 |
| ko00430 | Taurine and hypotaurine metabolism | Metabolism | 0.03737537 | 2 | 11 |
| ko03060 | Protein export | Genetic Information Processing | 0.04406491 | 3 | 28 |
| ko04340 | Hedgehog signaling pathway | Environmental Information Processing | 0.04675996 | 4 | 48 |
| ko04141 | Protein processing in endoplasmic reticulum | Genetic Information Processing | 0.04851335 | 10 | 193 |


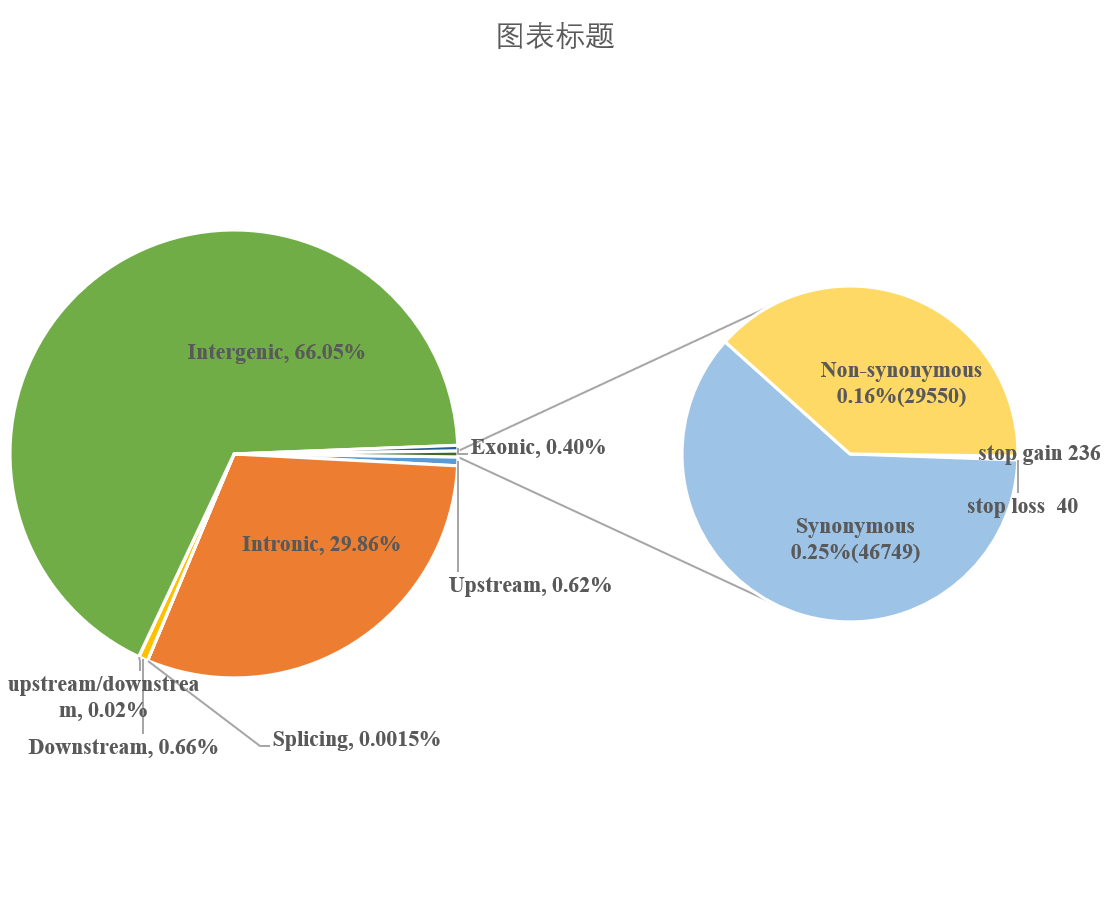


**Figure S1** Functional annotation of the identified SNPs in goat breeds


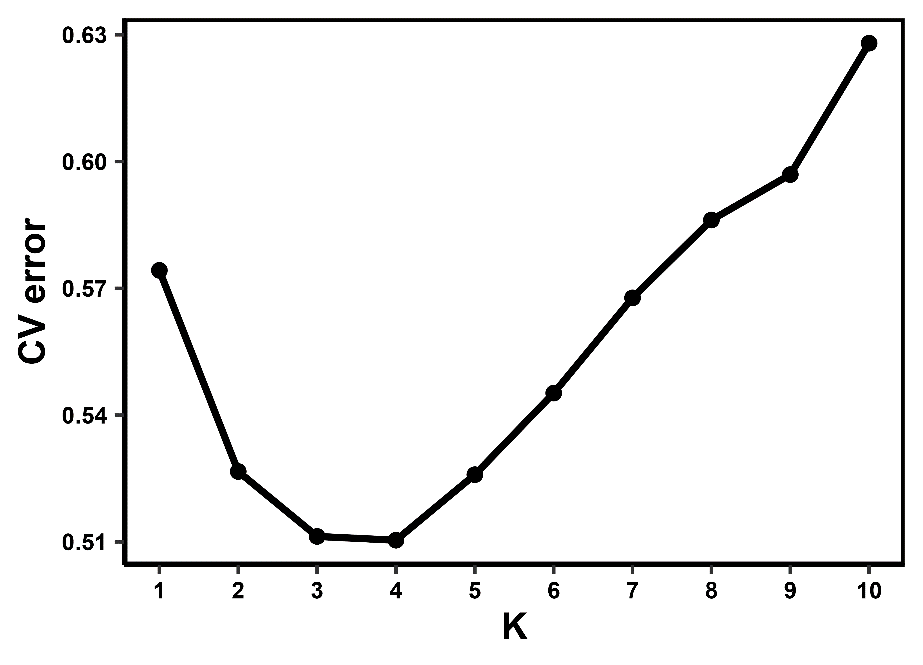


**Figure S2** The Cross-validation plot for admixture analysis.


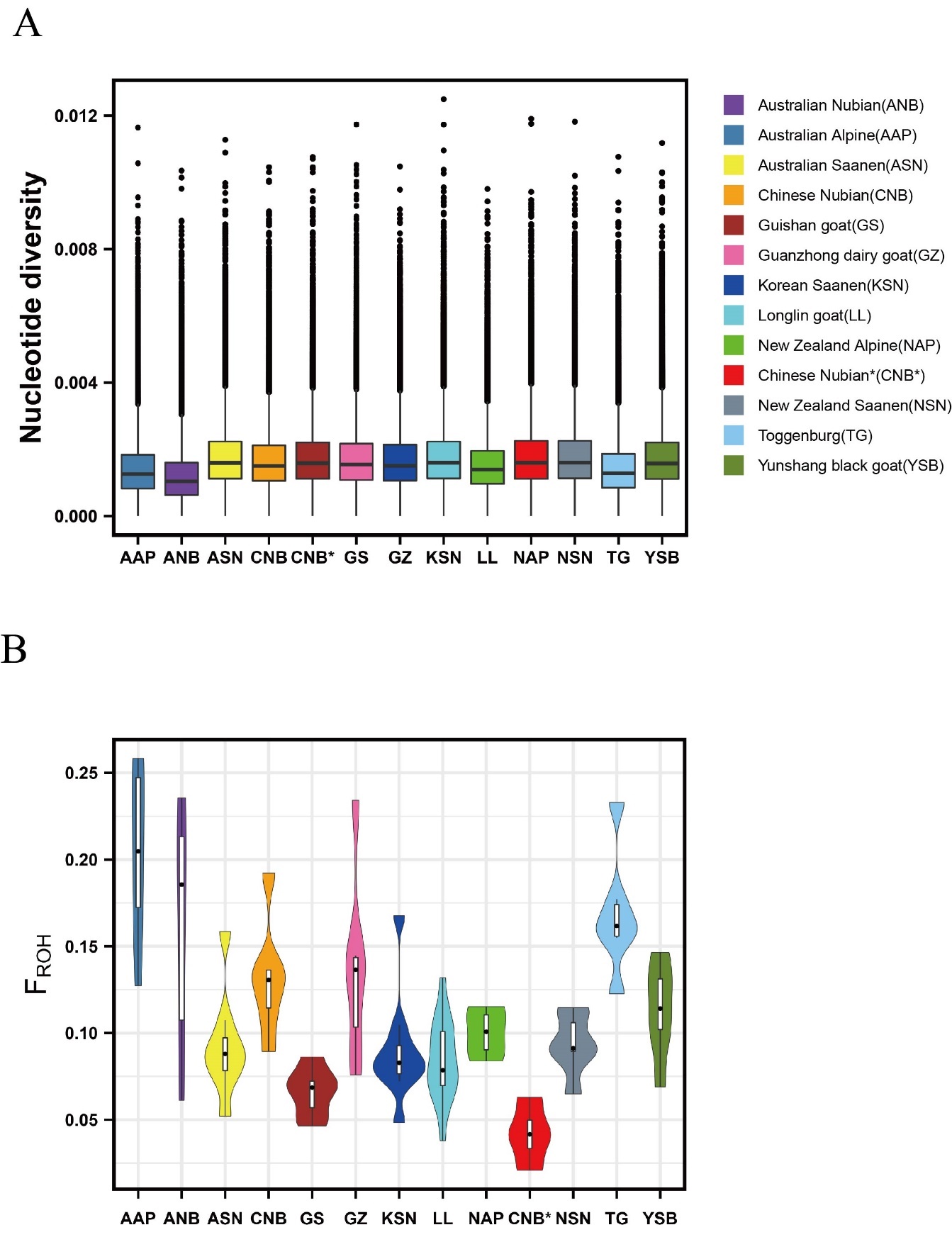


**Figure S3** (A) The boxplot indicates the distribution of nucleotide diversity of each breed in 50-Kb windows with 25-Kb steps. (B) Inbreeding coefficient for each breed


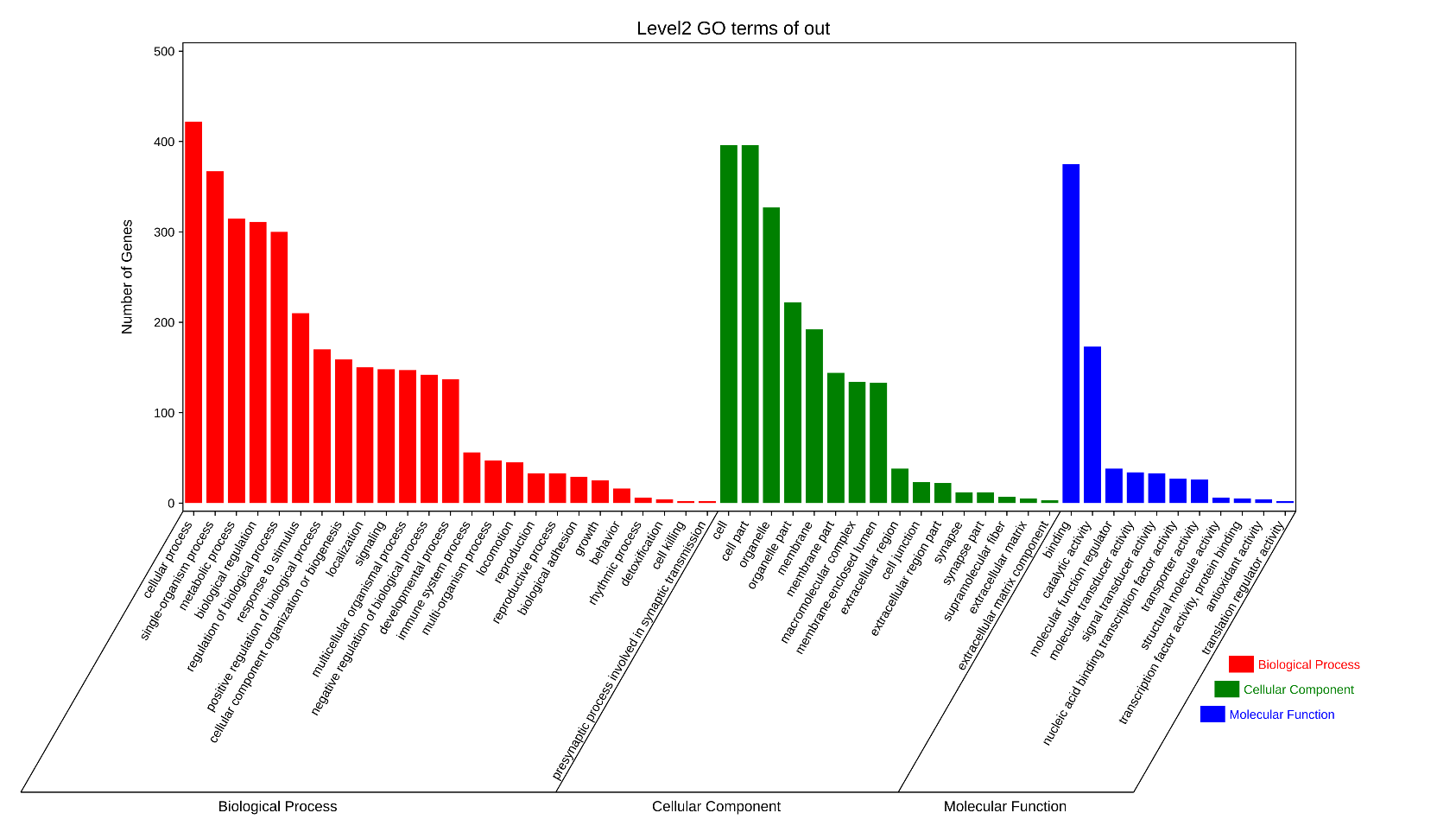


**Figure S4** Gene Ontology (GO) entries


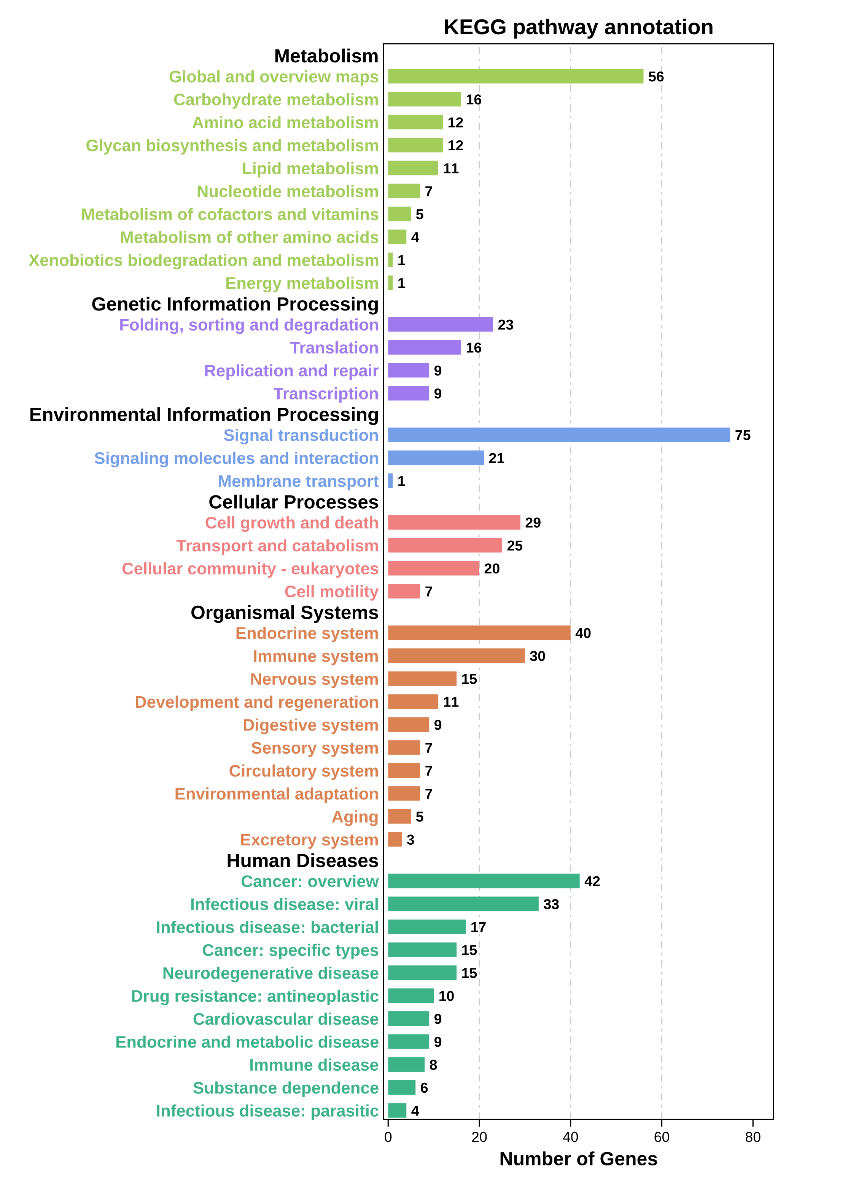

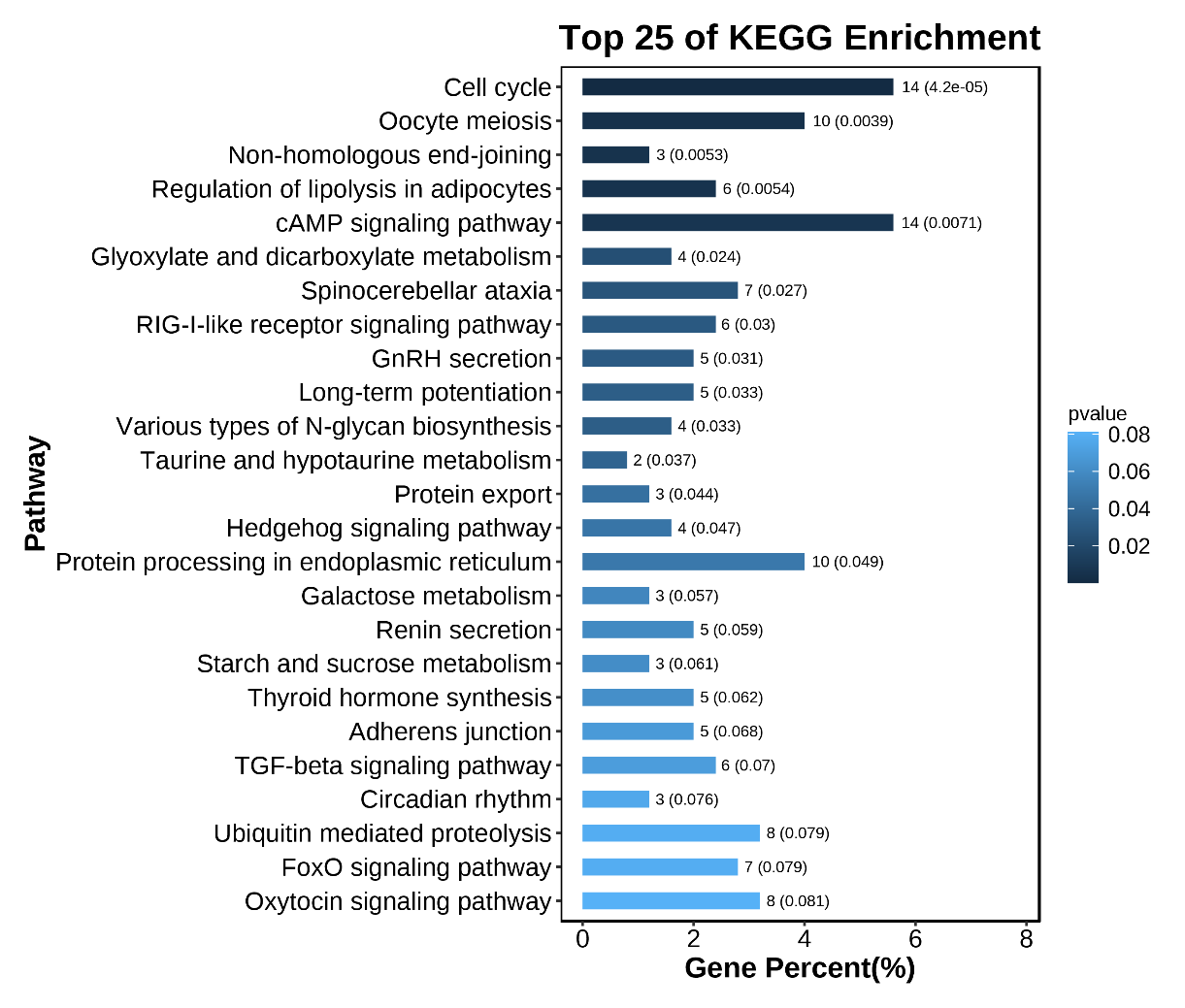


**Figure S5** KEGG pathway **Figure S6** Significantly enriched KEGG pathways
